# Integrated study of rhizosphere microbiome and metabolome profiles across tropical plantations in the Western Ghat regions of Kerala and Tamil Nadu, India

**DOI:** 10.3389/fmicb.2026.1840024

**Published:** 2026-05-13

**Authors:** Illathu Kandy Nidhin, Indranil Chattopadhyay

**Affiliations:** Department of Biotechnology, Central University of Tamil Nadu, Thiruvarur, Tamil Nadu, India

**Keywords:** metabolites, rhizosphere, soil bacteria, tropical forest, Western Ghats

## Abstract

**Background:**

Soil physicochemical properties, plantation types, plant taxa, and geographical locations are the major factors influencing the microbial composition and diversity of the rhizosphere soils.

**Methods and results:**

The present study aimed to investigate the variations in microbiome and metabolome composition across rhizosphere soils of plantations in the Western Ghats, based on ‘plantation types’ (mixed-species and mono-species), ‘sampling locations’ (Kerala and Coonoor), ‘plant types’ (shrubs and trees) and ‘plant genera’ (Eucalyptus, Pine, Silver oak, Tea, Rubber and Coffee); using 16S rRNA sequencing and GC–MS analysis. The mono-species samples had higher concentrations of K and heavy metals, such as Pb, Cr, Ni, and Cu than the mixed-species samples. The soil dehydrogenase activity (DHA) was highest in the mono-species tea and coffee samples from Kerala. However, the mixed-species pine and eucalyptus had comparatively higher DHA values than the mono-species pine and eucalyptus. *Paludisphaera borealis*, *Candidatus Solibacterusitatus*, *Candidatus Xiphinematobacter* sp. Idaho Grape, *Gemmatimonas phototrophica*, and *Conexibacter woesei* had significantly higher abundance in mixed-species samples based on the LEfSe analysis (LDA score>3 and FDR-adjusted *p*-value < 0.05). Whereas, *Chthoniobacter flavus*, *Tepidisphaera mucosa*, *Acidibacter ferrireducens*, *Paludibaculum fermentans*, and *Gaiella occulta* had significantly higher abundance in mono-species samples. The strong positive correlations (r values >0.9 and <−0.9, with *p*-value < 0.05) of bacterial taxa with plant metabolites such as phytane, Friedelan-3-one, gamma-gurjunene, eucalyptol, (−)-globulol and epiglobulol, indicate that these plant metabolites may play crucial roles in the differential bacteriome compositions of these rhizospheres.

**Conclusion:**

This study provides new insights into the alterations in the rhizosphere bacteriome across various plantations in the Western Ghats region based on plantation type, plant genera, metabolites, and other soil physiochemical properties.

**Impact statement:**

Plantation type and sampling location were the factors that significantly influenced the rhizosphere metabolome and microbiome profiles, followed by plant genera and plant type.

## Graphical abstract

**Figure:**
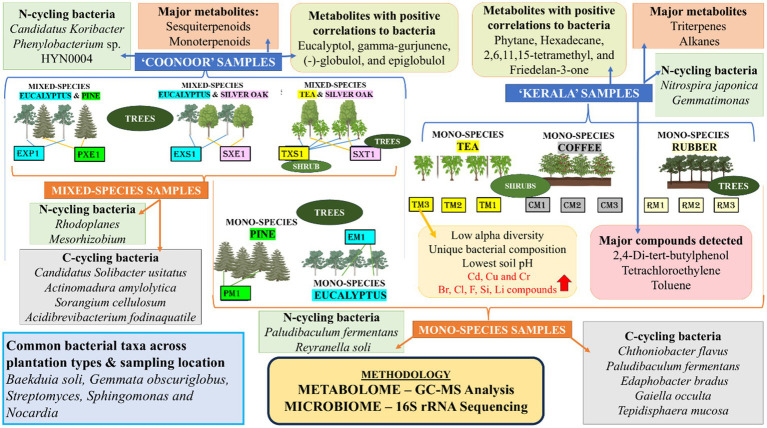

## 1 Introduction

The soil physiochemical properties, vegetation type and geographic parameters of the sampling locations including altitude, were the major factors affecting the soil bacteriome composition and diversity across the forest soils collected from the Western Ghats regions of Maharashtra (Ghare et al., 2023). Various climate change events have differential impacts on soil bacterial compositions (Lladó et al., 2017) and the soil microorganisms play crucial roles in nutrient cycling and ecosystem functions (Xia et al., 2016), particularly in the C, N, and P cycles, as well as in the fate of organic pollutants and heavy metals in the soil (Song et al., 2024). The soil zones surrounding the plant roots, which have a direct influence on plant root exudates, are termed the rhizosphere. The rhizosphere microbiomes are regarded as the “second genome” of plants (Sharma et al., 2020), because they contribute to the nutrition, defence, growth, and stress tolerance of plants (Fourneau et al., 2024). The rhizosphere is a site of active carbon cycle processes, as well as a site of carbon-rich root exudation by the plants (Nuccio et al., 2020) and harbours microbes enriched with carbohydrate-active enzyme (CAZy) genes (Pausch and Kuzyakov, 2018). Hence, the fate of carbon and other nutrients in the rhizosphere may be governed by plant-root exudate-microbe interactions (Wu et al., 2023). The root exudates from plant roots, such as, amino acids, organic acids, carbohydrates, and metabolites; acts as chemo-attractants and nutrient source for soil microbes, thereby play crucial roles in root colonisation and plant–microbe interactions (Chagas et al., 2018; Rolfe et al., 2019; Fourneau et al., 2024). In addition to root exudates from plants, microbial metabolites also contribute substantially to the soil metabolome (Song et al., 2024). Microbes modulate root exudates via “systemically induced root exudation of metabolites (SIREM),” which are composed of fatty acids, aliphatic and aromatic alcohols, and organic acid derivatives (Korenblum et al., 2020; Korenblum et al., 2022). Phytochemical niche partitioning amongst plants and phytochemical compositions are strongly influenced by biotic and abiotic pressures (Oppenheimer-Shaanan et al., 2022; Zhuang et al., 2024). Litterfall also serves as a major source of plant metabolites in the soil layer (Chomel et al., 2016). Abiotic pressures such as drought exposure increase the number of amino acids, sugar alcohols, fatty acids, and phenolic glycosides. Rhizosphere microbial diversity is positively correlated with several metabolites (Veach et al., 2020). Stressed plants tend to recruit beneficial microbes to their rhizosphere by releasing specialised compounds through root exudates, thereby enhancing growth-promoting traits in the rhizosphere to ensure the fitness and survival of beneficial microbes, allowing them to thrive in adverse conditions as holobionts; this phenomenon is known as the ‘cry-for-help’ effect (Rolfe et al., 2019; Rolli et al., 2021). However, the ‘cry-for-help’ phenomenon is less effective in mono-species fields or plantations because of the long-term existence of a single plant genus in those habitats (Zitzelsberger and Buchbauer, 2015).

Forests are recognised as efficient carbon sinks and key contributors to primary productivity owing to their relatively high biomass and high diversity of flora (Bonan, 2008). Compared with plants with lower biomass and shorter lifespans, forest soil is more heavily influenced by trees. Moreover, the root exudates, canopy, biomass, water, and oxygen uptake of trees significantly influence the soil chemistry, moisture, temperature, and porosity (Augusto et al., 2015; Dukunde et al., 2019). Tree genera and plantation type have crucial impacts on soil bacterial composition and diversity in the temperate deciduous forests of Hainic National Park located, Thuringia, Germany (Dukunde et al., 2019). Moreover, the mixed-species plantation soils had higher bacterial diversity than the mono-species plantation soils from South China (Zheng et al., 2024). A comparative study of the rhizosphere microbiomes of spatially similar mixed and mono stands has not been conducted (Uroz et al., 2016). There is a lack of research regarding the impact of tree species and plantation type on rhizosphere soil bacterial populations in tropical trees in the Western Ghat region of India.

The Nilgiris, situated in the Western Ghats, is India’s first UNESCO Biosphere Reserve and the Nilgiris Plateau was covered by grasslands and shola forests during the nineteenth century (Satish et al., 2014). The Nilgiris mountain regions have been subjected to plantations of various non-native and alien tree species since the British colonisation period (mainly from 1850 to 1945), and these tree plantations persist in these regions. *Eucalyptus*, *Pinus*, *Acacia*, *Cinchona*, and *Cyprus* are the major tree species used for these plantation processes (Jeevith and Manjunath, 2023). Eucalyptus is a widely planted, fast-growing tree genus of the Myrtaceae plant family that can thrive in any habitat (Nasir Shah et al., 2023). Silver oak trees are used as shade trees in tea and coffee agroforestry and forest plantations. Moreover, they are fast-growing trees that can even thrive in poor soil conditions (Musongora et al., 2023). Silver oaks are the most widely planted tree species in the urban regions of India (H. Singh et al., 2020).

The present study aimed to elucidate the bacteriome, functional gene, and metabolite- profiles of the rhizospheric soils of pine, eucalyptus, silver oak, tea, coffee, and rubber trees via culture-independent 16S rRNA gene amplicon sequencing and non-targeted GC–MS analysis. We hypothesised that the bacterial and metabolite compositions and diversities across the tropical vegetation in the Western Ghats region of India may be greatly determined by plantation types, sampling locations, plant genera, and plant type. Our study aims to address the following questions: which parameters leads to higher variations in the soil microbial diversity and composition across the samples? What are the effects of soil physicochemical properties and heavy metal concentrations on the rhizosphere across the samples? Which tree/plantation types best suit the Western Ghat regions to sustain rich microbial diversity? The results of this study could help improve the sustainable development of tropical vegetation and plantations in the Western Ghats region.

## 2 Methodology

### 2.1 Sample site description

Rhizosphere soil samples were collected from the Coonoor and Kerala regions (Wayanad) of the Western Ghats. Coonoor is a hill town and urban municipality in the Nilgiris district of Tamil Nadu, situated at an altitude of 6,050 feet (1,850 metres) above sea level, with an area of 15.05 square kilometres and a population of 45,494.^1^ According to Indian Meteorological Department data from 1991 to 2020, Coonoor experienced mean annual temperatures of 13.7 °C (minimum) and 22.1 °C (maximum). The mean annual precipitation received in Coonoor is 1667.7 mm, and 80 days of the mean number of rainy days per year are reported in the Coonoor region.^2^ Wayanad is located in the Western Ghats region of northern Kerala at an altitude of 2,500–6,000 feet (700–2,000 metres) above sea level.^3^ Both Wayanad and Coonoor are the regions of the Western Ghats with several commercial and non-commercial plantations and have similar reddish-brown lateritic soil type. Both Coonoor and Wayanad had the lowest rainfall and lowest temperature during the month of sample collection. However the elevation and temperature difference between the locations may affect to the microbial composition, which will be reflected in the analyses under the ‘sampling location’ grouping of the samples. No samples were collected from the protected areas; all the selected sampling sites were plantations or commercial estates situated outside the protected or conserved areas.

### 2.2 Sample collection

Rhizosphere soil samples of Eucalyptus (*Eucalyptus*), Pine (*Pinus*), Silver oak (*Grevillea*), Rubber (*Hevea brasiliensis*), Coffee (*Coffea arabica*) and Tea (*Camelliasinensis*) were collected. Coffee, rubber, tea, eucalyptus, and pine were the mono-species plantations (sites having only single-genus vegetation cover) selected, and various mixed plantation samples of Eucalyptus, pine, silver oak, and tea were collected. The selection of mixed plantation sampling sites was based on the availability of sites with vegetation cover composed of only two tree genera and with similar tree density and ages across the sites. The tree density and ages were uniform across the sample sites because we have selected trees only from the plantation sites. These plantation sites were established around the same time-period. Moreover, the plantations followed the same pattern of planting trees across these sites. Hence, age and tree density did not vary much across the sites. The samples were collected during the winter season, specifically in January 2023. Coonoor receive the lowest monthly rainfall in January, with an average of 13 mm. The lowest mean temperature during this month was ~15 °C, and the highest mean temperature was ~27 °C.^4^ During the month of January, Wayanad experiences a minimum mean temperature of 17 °C and a maximum mean temperature of 28 °C. Moreover, similar to Coonoor, Wayanad also receives the lowest monthly rainfall of 12 mm in January.^5^ A total of 17 sets of composite samples of rhizosphere soils were collected: 8 from Coonoor and 9 from Wayanad (Kerala). The sampling sites from Coonoor were Eucalyptus mono-species (11°22′04.7″N, 76°45′28.3″E), pine mono-species (11°22′51.0”N, 76°48′19.7″E), Eucalyptus-Pine mixed-species plantations (11°22′53.2”N, 76°48′17.4″E), Eucalyptus-Silver oak mixed-species plantations (11°23′01.1”N, 76°46′22.7″E) and silver oak-Tea mixed-species plantations (11°22′59.8”N, 76°46′22.6″E) (Table 1). One set of composite samples was collected from each mono-species plantation site, *viz*., EM1 (from the Eucalyptus mono-species site) and PM1 (from the Pine mono-species site). Two sets of composite samples were collected from each mixed-species plantation site, corresponding to the two tree genera at each site (Supplementary Figure S1). A total of 6 mixed-species plantation composite samples were collected from the above-mentioned mixed-species plantation sites, *viz*., PXE1 (rhizosphere soil samples of pine trees from the Eucalyptus-Pine mixed-species site), EXP1 (rhizosphere soil samples of Eucalyptus trees from the Eucalyptus-Pine mixed-species site), EXS1 (rhizosphere soil samples of Eucalyptus trees from the Eucalyptus-Silver oak mixed-species site), SXE1 (rhizosphere soil samples of silver oak trees from the Eucalyptus-Silver oak mixed-species site), SXT1 (rhizosphere soil samples of silver oak trees from the Silver oak-Tea mixed-species site) and TXS1 (rhizosphere soil samples of tea plants from the Silver oak-Tea mixed-species sites). From Wayanad, three plots of mono-species coffee plantations were selected from a coffee estate near Muttil, namely, CM1 (11°37′52.8″N 76°06′27.9″E), CM2 (11°37′53.1″N 76°06′26.0″E), and CM3 (11°37′49.3″N 76°06′27.7″E). Similarly, three plots of mono-species tea plantations were identified in Vythiri, namely, TM1 (11°32′43.0″N 76°02′47.5″E), TM2 (11°32′42.8”N 76°03′24.4″E), and TM3 (11°32′50.9”N 76°02′44.0″E). Moreover, mono-species rubber plantation plots were selected near Thamarassery, namely, RM1 (11°26′41.7″N 75°57′23.3″E), RM2 (11°26′42.3″N 75°57′22.1″E) and RM3 (11°26′40.2″N 75°57′21.8″E). The coffee, rubber and tea plantations for which we were able to obtain permission (from the respective owners of these private plantations) for sample collection were completely mono-species plantations. Hence, we were able to collect only mono-species samples from these locations from Kerala. Each composite sample was obtained by pooling replicate samples from five randomly selected individual trees which are minimum ~10 m apart from each other. Rhizosphere soils were collected by gently dragging the hand shovels along the root portions of each tree (at least 2–3 spots per tree) at a depth of 10–20 cm (after removing the litter layer), and the samples were carried in cool conditions (4 °C) to the laboratory (Lacerda-Júnior et al., 2019; Zhang et al., 2022; Yang et al., 2024). The soil samples are then air-dried at room temperature (28 °C), inside the laminar air flow (LAF) chamber for 24 h with 71% of humidity and sieved using sterilised 2 mm sieves. The sieved soil samples for DNA extraction were stored at −80 °C and the remaining soil samples were split into two; the samples for physicochemical analysis and GC–MS analysis were stored at −20 °C. Whereas, the samples for the soil dehydrogenase assay were stored at 4 °C.

**Table 1:** Sample collection site and sample name details.

Tree genera of rhizosphere soil collection (1st alphabet)	Vegetation type (2nd alphabet)	Co-existing tree genera in the mixed plantation site (3rd alphabet)	Sample name	Geographic location (locality name)	Altitude
Pine (P)	Mixed-species plantation (X)	Eucalyptus (E)	PXE1	11°22′53.2″N 76°48′17.4″E (Yedapalli, Coonoor)	2,097 m
Eucalyptus (E)	Mixed-species plantation (X)	Pine (P)	EXP1		
	Mixed-species plantation (X)	Silver oak (S)	EXS1	11°23′01.1″N 76°46′22.7″E (Jagathala, Coonoor)	1,870 m
Silver oak (S)	Mixed-species plantation (X)	Eucalyptus (E)	SXE1		
	Mixed-species plantation (X)	Tea (T)	SXT1	11°22′59.8″N 76°46′22.6″E (Jagathala, Coonoor)	1,868 m
Tea (T)	Mixed-species plantation (X)	Silver oak (S)	TXS1		
Eucalyptus (E)	Mono-species plantation (M)	-	EM1	11°22′04.7″N 76°45′28.3″E (Aruvankadu, Coonoor)	1,941 m
Pine (P)	Mono-species plantation (M)	-	PM1	11°22′51.0”N 76°48′19.7″E (Yedapalli, Coonoor)	2,090 m
Coffee (C)	Mono-species plantation (M)	-	CM1	11°37′52.8″N 76°06′27.9″E (Muttil, Wayanad, Kerala)	796 m
		-	CM2	11°37′53.1″N 76°06′26.0″E (Muttil, Wayanad, Kerala)	789 m
		-	CM3	11°37′49.3”N 76°06′27.7″E (Muttil, Wayanad, Kerala)	804 m
Tea (T)		-	TM1	11°32′43.0″N 76°02′47.5″E (Vythiri, Wayanad, Kerala)	778 m
		-	TM2	11°32′42.8”N 76°03′24.4″E (Vythiri, Wayanad, Kerala)	866 m
		-	TM3	11°32′50.9″N 76°02′44.0″E (Vythiri, Wayanad, Kerala)	775 m
Rubber (R)		-	RM1	11°26′41.7″N 75°57′23.3″E (Thamarassery, Kerala)	68 m
		-	RM2	11°26′42.3″N 75°57′22.1″E (Thamarassery, Kerala)	71 m
		-	RM3	11°26′40.2″N 75°57′21.8″E (Thamarassery, Kerala)	63 m

In the sample names, the first alphabet represents the tree genera from which the rhizosphere soil samples are collected: E-eucalyptus, P-pine, S-silver oak, and T-tea. The second alphabet denotes the plantation types: M, mono-species, and X, mixed-species. The third alphabet is assigned only to the mixed plantation sample and represents the co-existing tree genera in the mixed plantation site: E-eucalyptus, P-pine, S-silver oak, and T-tea. Thamarassery is situated outside the official boundaries of the Wayanad district.

### 2.3 Soil physicochemical and heavy metal analysis

Soil pH, nitrogen (N), available nitrogen (AN), nitrate nitrogen (NN), phosphorus (P), available phosphorus (AP), total organic carbon (TOC), potassium (K), sodium (Na), magnesium (Mg), calcium (Ca), and heavy metals likelead (Pb), nickel (Ni), cadmium (Cd), chromium (Cr), zinc (Zn), copper (Cu), manganese (Mn), iron (Fe) and aluminium (Al) were analysed via various techniques. The soil pH was estimated at a 1:2 ratio of soil and water [10 g of soil and 20 mL of water (Bárcenas-Moreno et al., 2022)] via a bench top pH meter (Labman LMPH-12). The concentrations of N, AN, NN, P, and TOC were estimated by our group (Vasanthrao et al., 2025). The detection limits of these methods were as follows (respective nutrient-elements are given in the brackets): Walkley-Black method (TOC)—0.01%, Bray-Kurtz method (P)—10 mg/kg, Kjeldahl method (N)—10 Kg/ha, Subbia-Asija method (AN)—5 mg/kg, and phenol di-sulfonic acid method (NN)—1.0 mg/kg. AP was estimated using the molybdate–ascorbic acid method (Arias et al., 2021). To estimate the K, Na, Mg, and Ca concentrations, 5 g of soil samples were thoroughly mixed with 1 N NH4OAc (pH 7), and the filtrates were collected with Whatman No. 1 filter paper. Ca and Mg were estimated via the EDTA titration method, whereas K and Na were estimated via a flame photometer (Ukabiala et al., 2021). Pb, Ni, Cd, Cr, Zn, Cu, Mn, Fe, and Al were analysed via an atomic absorption spectrophotometer (AAS) (Varian SpectraAA 220). Briefly, 5 g of each soil sample was processed via acid digestion using HNO₃-HClO₄ mixture (4,1) with the hot-plate digestion method, cooled, and diluted to 100 mL to estimate the heavy metal concentration. Blank samples or negative controls were processed in a similar manner. Standard solutions (6 concentrations) of the various heavy metals were prepared. The prepared solutions were aspirated into the AAS machine in the following order: blank, standard, and sample. The wavelengths used to measure the absorbance of different heavy metals using the SpectrAA 4.1 software (Agilent) are mentioned in the Supplementary Table S1.

### 2.4 Soil dehydrogenase activity

Dehydrogenase activity was analysed via the methodology described by Seo and Cho (2021). Air-dried soil samples (0.5 g) were weighed and transferred into 15 mL centrifuge tubes. Two milliliters of freshly prepared Tris–HCl buffer (pH 7.6) were carefully added to each of the tubes, followed by the addition of 1 mL of 1% (w/v) triphenyl tetrazolium chloride (TTC) (Sisco Research Laboratories Pvt. Ltd., India). The tubes were vortexed to mix the reaction mixture thoroughly and then incubated in the dark at 37 °C for 24 h (Mir et al., 2023). After 24 h, triphenyl tetrazolium Formazan (TTC formazan/TPF) was formed and extracted with 10 mL of 96% ethanol. The tubes were vortexed thoroughly after adding ethanol to properly mix the contents and then centrifuged for 5 min at 4,000 × g. The amount of TPF in the supernatant was estimated by measuring the red colour intensity at 485 nm via a UV/Vis spectrophotometer (Eppendorf AG 22331, Hamburg, Germany). The amount of TTC converted to TPF was calculated from the OD readings with the help of a standard curve prepared using various concentrations (0–10 μg/mL) of TPF (Sisco Research Laboratories Pvt. Ltd., India) (Ghaly and Mahmoud, 2007). The soil dehydrogenase activity was expressed as μg TTC g^−1^ h^−1^ (Meena and Rao, 2021).

### 2.5 Identification of the metabolites using GC–MS analysis

Sample extraction was performed according to the procedures outlined by Selvaraj et al. (2015) and Elaiyaraja et al. (2022), with minor modifications (methanol was used as the solvent in place of ethyl acetate). The soil samples were properly air-dried and homogenised before sample extraction. One gram of the soil sample was weighed and transferred into an amber glass bottle; 20 mL of methanol was added, and the bottle mouth was covered with aluminium foil. The samples were sonicated for 30 min using an ultrasonicator. The sonicated sample was decanted into a conical flask and dehydrated with 1 g of sodium sulphate. The condensed extract was reconstituted with 1 mL of methanol and transferred into glass vials for GC–MS analysis. The samples extracted into the vials were loaded into a GC–MS instrument (Agilent Model: CH-GCMSMS02, 8,890 GC System, 7,000 GC/TQ). Helium was used as the mobile phase and carrier gas, and nitrogen was used as the collision gas. Analyte separation was achieved using a column with dimensions of 30 m × 250 μm × 0.25 μm. The initial temperature was set at 50 °C for 1 min and then raised to 120 °C at 5 °C/min, further increased to 210 °C at 10 °C/min, and finally reached a temperature of 280 °C at 10 °C/min, which was held for 5 min. The total run time was 38 min. The scan range was 30–900 m/z, and the data were analysed via Mass Hunter software. The chromatogram peaks obtained from the Mass Hunter software were utilised for non-target hit library search using the NIST 17 library (National Institute of Standards and Technology) for compound identification based on the ‘retention time’ (Ueberschaar et al., 2021).

Compound lists with component retention times, compound names, component areas, match factors, CAS numbers, and chemical formulas were obtained after the library search. The compound lists were rearranged based on the ‘component area,’ and the top 100 compounds with the highest component areas were identified. The chemical classification of these compounds was achieved using Classyfire,^6^ and the IUPAC names of the compounds obtained from PubChem were used as input data on the Classyfire website. The significance and biological relevance of the compounds were detected via PubChem, and ChEBI. The top 100 compounds from the samples were compared using a Venn diagram^7^ to identify common and unique compounds present in the samples.

### 2.6 DNA extraction from the soil samples

The soil samples were air-dried, sieved, and used for DNA extraction using the DNeasy Power Soil Pro Kit (QIAGEN, Hilden, Germany) following the manufacturer’s instructions. The quality and quantity of the isolated DNA were determined using a Nanodrop spectrophotometer (Genova Nano, Jenway, UK) with the 260/280 nm ratio. DNA integrity was assured using 1% agarose gel electrophoresis.

### 2.7 Bacterial 16S rDNA amplification and library preparation

PCR amplification of the 16S rDNA V3-V4 region of bacterial DNA extracted from DNA samples (40 ng) from soils were performed using universal primers (10 pM each) (16sF, 5′-AGAGTTTGATGMTGGCTCAG-3′ and 16sR, 5′-TTACCGCGGCMGCSGGCAC-3′). The PCR reaction mixture consisted of bacterial DNA, primers, a master mix of dNTPs (0.5 mM), MgCl_2_ (3.2 mM), high-fidelity DNA polymerase, and PCR enzyme buffer. The PCR was performed at 95 °C for 3 min, followed by 25 cycles of 95 °C for 15 s, 60 °C for 15 s, and 72 °C for 120 s, with a final elongation step at 72 °C for 10 min. The quality of the generated 16S PCR products was verified on 2% agarose gels. To construct sequencing libraries, amplicons were cleaned with Ampure beads (Beckman Coulter Inc., Indianapolis, IN, United States) to eliminate unused primers, followed by 8 cycles of PCR with Illumina barcoded adapters. Libraries were purified with Ampure beads and quantified using the Qubit dsDNA High Sensitivity Assay kit (Thermo Fisher, Bangalore, India). Sequencing was performed using Illumina MiSeq with a 2x300PE v3-v4 sequencing kit at Biokart, India Pvt. Ltd., Bengaluru, India.

### 2.8 Bioinformatics and statistical analysis

The raw binary base call (BCL) file, which was generated during sequencing, was demultiplexed and converted to FASTQ format. FastQC (Version 0.11.9) and MultiQC (Version 1.10.1) were used to assess data quality after demultiplexing the raw sequencing data. TrimGalore (version 0.6.6) was used to remove contaminated adapters and trim low-quality reads. QC-passed samples were analysed using Quantitative Insights into Microbial Ecology (QIIME version 1.9.0) methodology, which included paired-end read fusion, chimaera removal, operational taxonomic unit (OTU) clustering, and taxonomy assignment. OTUs were selected based on Kraken2 with the NCBI database (Wood et al., 2019). All analyses, including rarefaction curve, *α*-diversity, *β*-diversity, core microbiome, cluster study, linear discriminant analysis effect size (LEfSe), and KEGG pathway analyses, were performed using MicrobiomeAnalyst (https://www.microbiomeanalyst.ca/),with the data filtering steps with a low count filter of minimum count 4 and prevalence of minimum 20% along with low variance filter of minimum 10% Inter-quantile range (IQR) (Lu et al., 2023). The Pearson correlation analysis was performed using PAST (Palaeontological Statistics) version 4.17 software, and the correlation matrix obtained was used to generate correlation network plots using Cytoscape (v3.10.3) software along with the Metscape plugin. The Generalised Linear Model (GLM) analysis was performed using the PAST (v4.17) software, using the ‘normal’ distribution and ‘identity’ link function. The non-targeted metabolite data from the GC–MS analysis were analysed using MetaboAnalyst 6.0.^8^ withthe data filter of a minimum of 5% IQR (Pang et al., 2024). Furthermore, taxonomy-to-phenotype mapping of the microbiome data was performed using METAGENassist^9^ with the default data filtering steps; IQR of 5% and removal of variables with over 50% zero values (Arndt et al., 2012). Sequence data supporting the outcomes of this work have been deposited in the NCBI-SRA database under the BioProject accession number PRJNA1358411.

## 3 Results

### 3.1 Soil physicochemical properties and soil dehydrogenase activity

**Figure 1:**
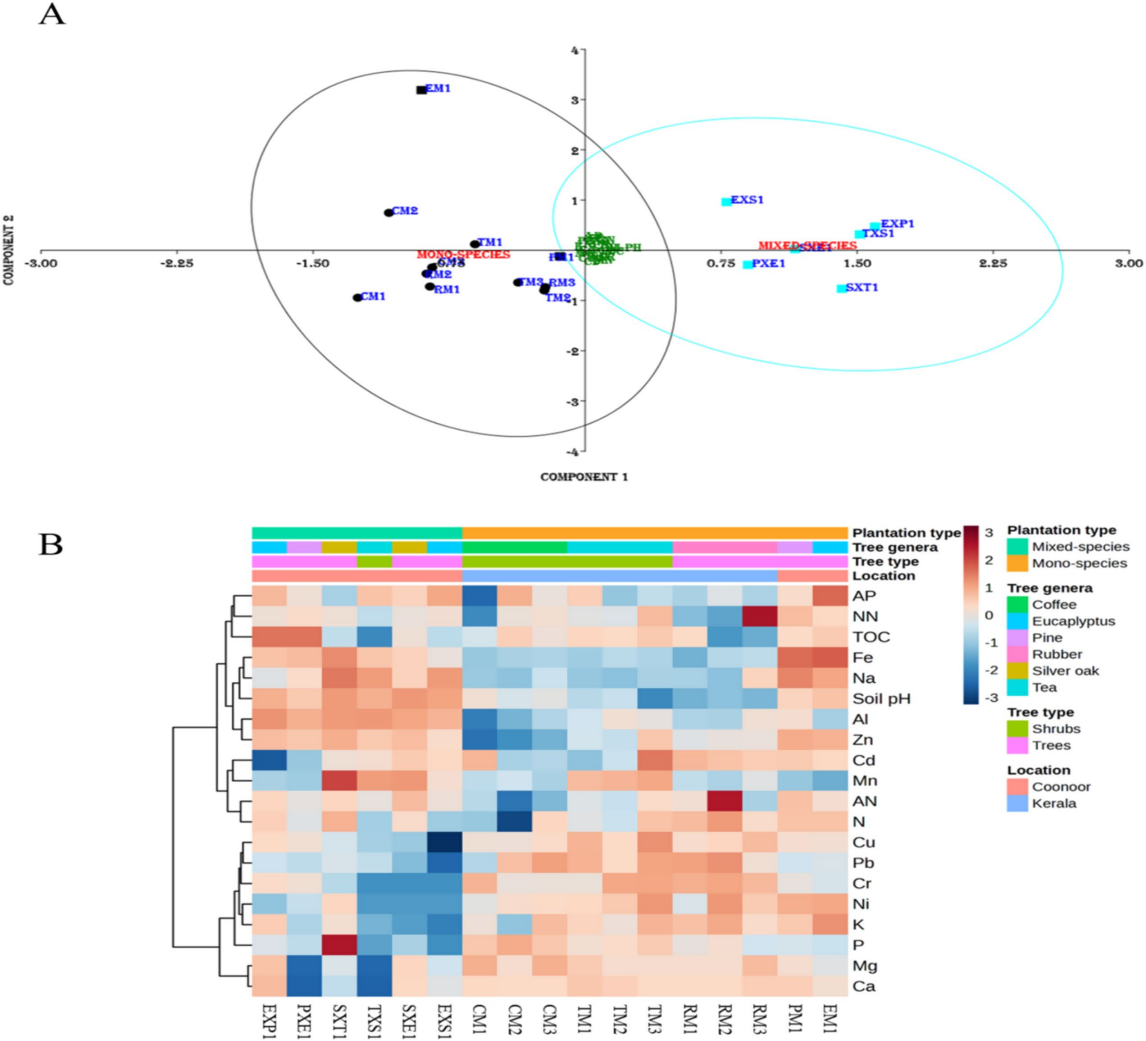
**(A)** The Principal Component Analysis (PCA) plot for the mixed and mono-species rhizosphere samples, based on the soil physicochemical characteristics and heavy metal concentrations, was generated using PAST 4.17 software. The ‘black’ symbols represent mono-species samples and the ‘blue’ symbols represent the mixed-species samples. Meanwhile, the samples from Kerala are represented by ‘round’ symbols and those from Coonoor by ‘square’ symbols. **(B)** Heat map depicting the soil physicochemical properties and heavy metal concentrations across the rhizosphere soil samples prepared using MetaboAnalyst 6.0. Euclidean distance measure and Ward’s clustering Algorithm were used to prepare the heatmap. The scale displays the Z-score measures, where the red boxes represent higher values (above the average), and the blue boxes represent lower values (below the average).

The soil physicochemical characteristics varied across the samples (Supplementary Table S2). The PCA plot (Figure 1A) shows that the soil physicochemical characteristics and heavy metal concentrations varied between the mixed and mono-species plantations. The mixed-species and mono-species groups were well separated from each other in the PCA plot and the separation was validated to be statistically significant (*p* = 0.0001) using the permutational multivariate analysis of variance (PERMANOVA) test (PAST v4.17). Although the PM1, EM1, and mixed species samples were collected from the same locality, the PM1 and EM1 samples differed from the mixed species samples. Moreover, both the EM1 and PM1 samples were similar to the other mono-species samples, regardless of the geographical distance between the locations. All the soil samples had slightly acidic to neutral pH values (ranging from 5.1 to 7.9). The samples from Kerala (6.03 ± 0.55) had slightly lower pH values than did the Coonoor samples (7.53 ± 0.28). The lowest soil pH values were found in the mono-species tea rhizosphere sample TM3 (5.1). However, the group average of the tea samples was 6.28 ± 0.62. The average pH value of the rubber samples was 5.64 ± 0.09, and those of the coffee samples were 6.61 ± 0.18. Amongst the samples from Coonoor, the lowest soil pH values were found in the pine tree rhizosphere samples (7.17 ± 0.1). The highest pH values were detected in the silver oak rhizosphere samples (7.85 ± 0.03), whereas those of the Eucalyptus were 7.55 ± 0.21 (Supplementary Table S3).

The heatmap (Figure 1B) shows that K and heavy metals, such as Pb, Cr, Ni, and Cu, were higher in the mono-species samples than in the mixed species samples. Moreover, the Al content was greater in the mixed-species samples than in the mono-species samples. The Fe, Na, and soil pH values were greater in all Coonoor samples than in those from Kerala. Mn was greater in the rhizosphere samples of silver oak and tea (both mono- and mixed-species). However, the coffee samples had lower Al and Zn concentrations. Mg and Ca had the lowest concentrations in the SXT1, TXS1 and PXE1 samples. NN was highest in RM3, and AN was highest in RM2. Compared to the mixed-species samples EXP1 (7.27%) and PXE1 (7.21%) the TOC content was lower in the mono-species plantation samples PM1 (1.34%) and EM1 (1.81%). The TM3 sample had the highest concentrations of Cu (10.26 ppm), Cd (0.654 ppm) and Cr (1.08 ppm). Whereas, the Ni concentrations were the highest in RM2 (0.79 ppm), followed by TM3 (0.77 ppm). Moreover, both the mono-species plantation samples of pine and Eucalyptus had higher concentrations of Cd, Ni, Fe, Zn, and Pb compared to their respective mixed-species samples. In contrast, higher concentrations of Cr were detected in EXP1 (0.178 ppm) and PXE1 (0.117 ppm) compared to PM1 (0.078 ppm) and EM1 (0.043 ppm). The levels of heavy metals across the samples were below the limits of the BIS (Bureau of Indian Standards for heavy metal) standards. However, when compared with the background levels of Indian soils (Rangasamy and Muniyandi, 2025) to analyse the potential contamination from these heavy metals, we found that the Cd and Zn concentrations of the TM3 samples exceeded the natural background levels. Moreover, the Zn concentrations of the samples from Coonoor (except EXS1) were also exceeding the natural background levels (Supplementary Table S4).

**Figure 2:**
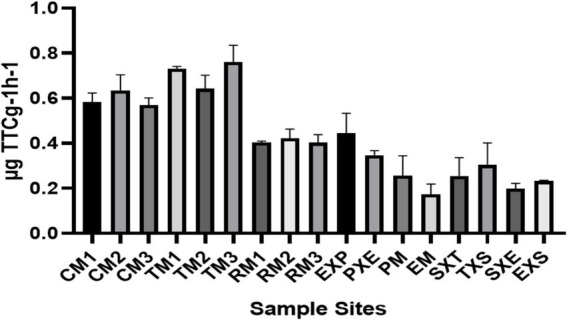
Bar graph depicting the soil dehydrogenase activity across the rhizosphere samples, expressed in μg TTCg^−1^ h^−1^.

The soil DHA plot (Figure 2) revealed that the tea and coffee mono-species samples from Kerala had higher dehydrogenase activity than did the rubber and Coonoor samples. The ANOVA-test revealed that soil DHA significantly differed across the samples (*p*-value 0.0056). The highest soil DHA was detected in tea mono-species sample TM3 (0.76 ± 0.07 μg TTC g^−1^ h^−1^), followed by TM1 (0.73 ± 0.01 μg TTC g^−1^ h^−1^) and TM2 (0.64 ± 0.06 μg TTC g^−1^ h^−1^). The lowest activity was reported in the EM1 (0.17 ± 0.04 μg TTC g^−1^ h^−1^). Amongst the Coonoor samples, soil DHA was the highest in the EXP1 (0.45 ± 0.09 μg TTC g^−1^ h^−1^) samples, followed by the PXE1 (0.35 ± 0.02 μg TTC g^−1^ h^−1^) and TXS1 (0.3 ± 0.09 μg TTC g^−1^ h^−1^) samples. However, the silver oak-tea mixed-species samples had a slightly greater soil DHA (SXT1 = 0.26 ± 0.08 μg TTC g^−1^ h^−1^, TXS1 = 0.3 ± 0.09 μg TTC g^−1^ h^−1^) than did the silver oak-eucalyptus mixed-species samples (SXE1 = 0.2 ± 0.02 μg TTC g^−1^ h^−1^, EXS1 = 0.23 ± 0.004 μg TTC g^−1^ h^−1^). The mixed-species plantations of eucalyptus and pine had higher DHA levels than did the mono-species plantations of eucalyptus and pine (PM1 = 0.26 ± 0.09 μg TTC g^−1^ h^−1^).

Furthermore, the Pearson correlation plot (Supplementary Figure S2) shows that significant correlations exist between the physicochemical parameters, heavy metal concentrations, and soil DHA. The soil pH was positively correlated with Na (*r* = 0.65, *p*-value = 0.004), Zn (*r* = 0.52, *p*-value = 0.03), Al (*r* = 0.68, *p*-value = 0.002), and Fe (*r* = 0.63, *p*-value = 0.007). Moreover, K, Pb, Ni, Cu, Cd, and Cr had significant negative correlations with soil pH. The heavy metals were positively correlated with each other, especially Pb, Cd, and Ni, which had significant positive correlations with Cr and Cu. Moreover, Cr has a positive correlation with Cu (*r* = 0.52, *p*-value = 0.03). Soil DHA was significantly negatively correlated with soil pH (*r* = −0.68, *p*-value = 0.003), Na (*r* = −0.76, *p*-value = 0.0003), Zn (*r* = −0.65, *p*-value = 0.004), Al (*r* = −0.5, *p*-value = 0.04), and Fe (*r* = −0.7, *p*-value = 0.002). Moreover, soil DHA was positively correlated with Pb (*r* = 0.6, *p*-value = 0.01), Cu (*r* = 0.64, *p*-value = 0.006), and Cr (*r* = 0.51, *p*-value = 0.03).

### 3.2 Major metabolites identified in the rhizosphere soils through non-targeted GC–MS analysis

**Figure 3:**
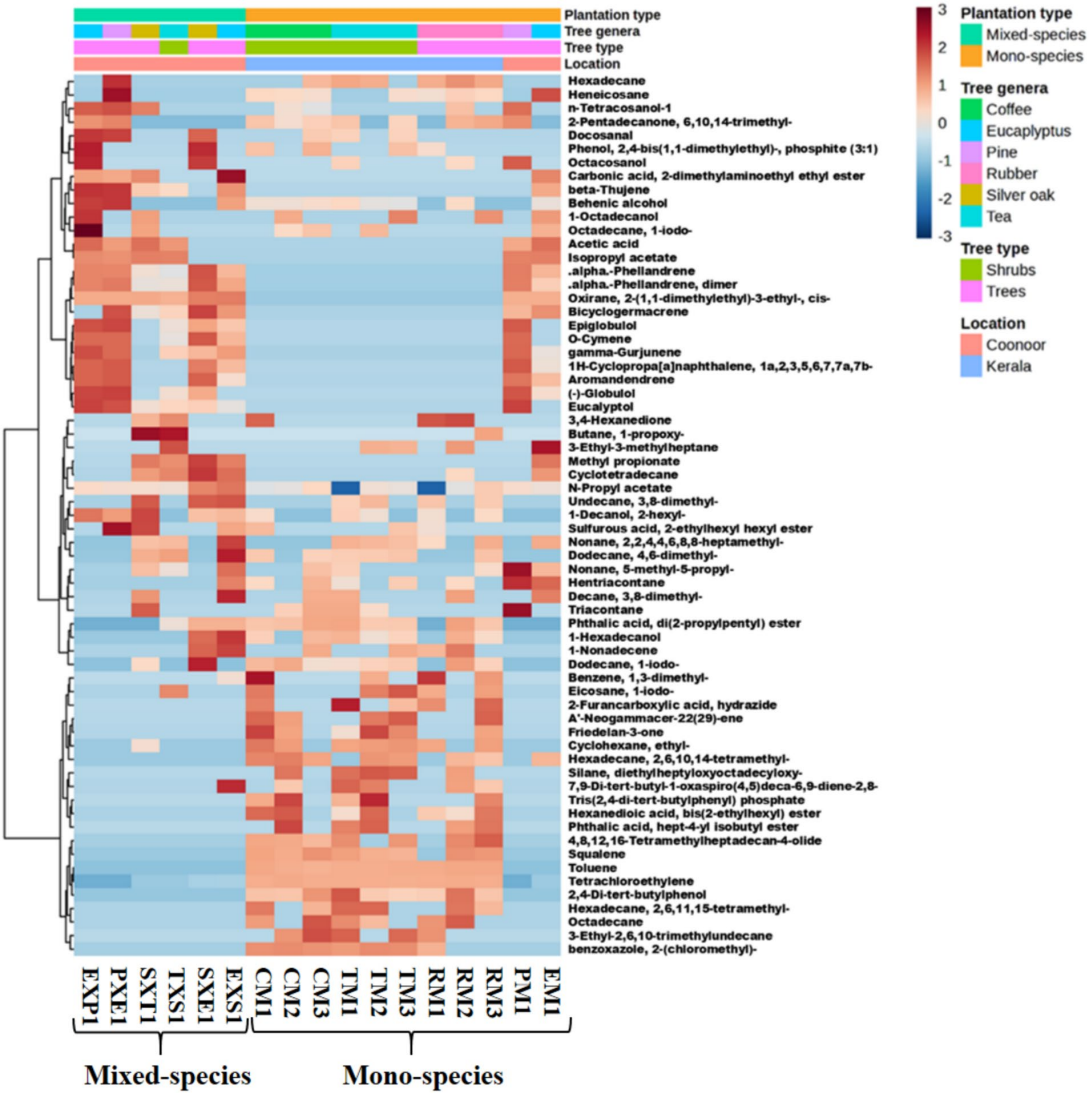
Heatmap of the metabolites detected across the 17 rhizosphere samples, generated using MetaboAnalyst 6.0. using Euclidean distance measure and Ward’s clustering Algorithm. 65 metabolites with higher ‘component area’ and present in at least 5 samples are represented here. The scale shows the Z-score measures; the red-coloured boxes represent higher values (above the average), and blue represents lower values (below the average).

The GC–MS results revealed 100–280 metabolites in each rhizosphere soil sample analysed. The compounds were identified based on the ‘retention time’ from the NIST library and only the compounds with a ‘match factor’ of a minimum of 50 were selected. A total of 200–280 metabolites were reported in thirteen samples, namely, EXP1 (257), PXE1 (276), PM1 (262), SXE1 (223), CM1 (247), CM2 (229), CM3 (225), TM1 (215), TM2 (217), TM3 (241), RM1 (211), RM2 (247), and RM3 (219). Four samples had 100–190 metabolites, namely, EM1 (126), SXT1 (107), TXS1 (117) and EXS1 (185). Amongst these compounds, only the top compounds with the highest ‘peak areas’ were selected for further analysis (Top) 65 compounds with highest ‘peak areas’ and present in at least 5 samples were used to compare the compounds across all 17 composite samples (Figure 3).

The major compounds detected across the samples from the Western Ghats are depicted in the heatmap (Figure 3). The list of compounds included plant metabolites; bacterial metabolites; and several pollutants such as alkanes, phthalic acid compounds, tetrachloroethylene, and toluene. The metabolites belonged to the following categories: monoterpenes, sesquiterpenes, triterpenoids, alkanes, organic acids, fatty acids, fatty alcohols, and fatty acid esters (Supplementary Table S5). Amongst these metabolites, only four metabolites were reported in all eight samples from Coonoor, namely, ‘alpha-Phellandrene dimer’ (C_20_H_32_), ‘alpha-Phellandrene’ (C_10_H_16_), ‘n-Propyl acetate’ (C_5_H_10_O_2_) and ‘Oxirane, 2-(1, 1-dimethylethyl)-3-ethyl-, cis-’ (C_8_H_16_O). Whereas 2, 4-Di-tert-butylphenol (C_14_H_22_O), tetrachloroethylene (C_2_Cl_4_), and toluene (C_7_H_8_) were the compounds detected in all nine samples collected from Kerala. Amongst these compounds, alpha-Phellandrene dimer, alpha-Phellandrene and n-Propyl acetate are plant metabolites. Whereas, 2, 4-Di-tert-butylphenol is a bacterial metabolite and tetrachloroethylene and toluene are not metabolites but are compounds that probably reach the soil as pollutants (Supplementary Table S5).

The top 74 compounds detected across the rhizosphere samples from Coonoor alone are depicted in the heatmap (Supplementary Figure S3). The heatmap shows that the EXP1 sample resembles PXE1. Similarly, SXE1 and EXS1 have similar metabolite compositions, with differences in the component areas of some compounds. The same pattern was found in the case of SXT1 and TXS1. The metabolite compositions of the PM1 samples resembled those of PXE1 and EXP1, probably due to the presence of pine trees common to both sample collection sites. The major metabolites detected across the rhizosphere samples from Kerala alone are depicted in the (Supplementary Figure S4). The major compounds detected only in the samples from Kerala were triterpenes, alkanes, branched alkanes, and aromatic hydrocarbons.

Principal component analysis (PCA) of these compounds revealed the top three principal components (PCs) that explained most of the variations in the data (Supplementary Figure S5). PC1 explained 47.3% of the variations, whereas PC2 and PC3 accounted for 9.9 and 7.2% of the variations, respectively. The PCA plot indicates that all Eucalyptus samples (green colour) differ from each other in their plant metabolite composition. The EXP1 sample closely resembles PXE1. Similarly, EXS1 and SXE1 have a similar metabolite composition, whereas EM1 shows similarities with SXT1 and TXS1. Moreover, the Pine samples (dark blue colour) had a similar composition of plant metabolites; i.e., both the mixed and mono-species plantations of Pine had similar plant metabolite profiles. The silver oak samples (violet colour) varied from one another; these samples showed more similarities with their respective mixed plantation trees (SXE1 had more similarity to the EXS1 sample, and the SXT1 sample had more similarity to the TXS1 sample). Moreover, all 9 samples from Kerala were closely associated with each other. All the mono-species coffee (red-coloured pyramids), tea (yellow-coloured pyramids), and rubber samples (light blue-coloured pyramids) are placed together, suggesting a similar metabolite composition across these samples. Principal component analysis (PCA) based on the metabolite profiles revealed the separation between the rhizosphere samples (Supplementary Figure S5). The PCA plot shows that the samples from Coonoor were well separated from those from Kerala (Supplementary Figure S5A). The mono-species samples EM1 and PM1 showed more relatedness to the mixed-species samples from Coonoor, rather than with other mono-species samples from Kerala. The PCA loading plot (Supplementary Figure S5B) reveals that tetrachloroethylene, toluene, friedelan-3-one, 2, 4-di-tert-butylphenol, benzene, 1, 3-dimethyl-, oxirane, 2-(1,1-dimethylethyl)-3-ethyl-, cis-, alpha-phellandrene, gamma-Gurjunene, eucalyptol, bicyclogermacrene, (−)-globulol, aromandendrene and acetic acid are the key compounds leading to the separations between the samples. The results show that the presence of tetrachloroethylene and toluene leads to significant variation in the metabolome profiles. Moreover, the higher number of plant metabolites suggests that the plant genera may also have a crucial role in the metabolome diversity across the samples. Overall, the results show that sesquiterpenoids, monoterpenoids, triterpenes, alkanes, branched alkanes, carboxylic acid esters, fatty alcohols, and aromatic hydrocarbons were the major groups of compounds detected across all the samples. The metabolites found only in the Coonoor samples were mostly sesquiterpenoids and monoterpenoids. The metabolites detected exclusively in the Kerala samples were mostly triterpenes, branched alkanes, and aromatic hydrocarbons.

### 3.3 Bacterial diversity across the rhizosphere samples

The total numbers of operational taxonomic units (OTUs) detected in the samples from Kerala and Coonoor were, 1,404 (2, 03,053 reads) and 573 (1, 36,210 reads), respectively. Overall, 1,819 unique OTUs of bacterial genera were detected from the total of 3, 39,263 reads. Earlier reports revealed that 1,174 OTUs of bacterial genera were detected from the 16S rRNA V3-V4 sequencing data of areca nut rhizosphere soils from Karnataka, India (Paulraj et al., 2021). The Venn diagram (Supplementary Figure S6A) shows that 158 OTUs were common between the Coonoor and Kerala samples. However, 415 and 1,246 OTUs were detected exclusively in the samples from Coonoor and Kerala, respectively. A total of 682 OTUs with relatively high absolute count values across the samples (a minimum absolute count value of 10 altogether in all the samples) were selected for further analysis. A total of 3, 24,776 reads were assigned to these 682 OTUs. The rarefaction curves (Supplementary Figures S6B,C) revealed that the sequencing depth was sufficient to capture almost the entire microbial diversity of the samples.

In all the further analyses, the rhizosphere samples are grouped based on four different criteria, namely, ‘plantation types’ (mixed and mono-species), ‘sampling locations’ (Kerala and Coonoor), ‘plant types’ (shrubs and trees) and ‘plant genera’ (Eucalyptus, Pine, Silver oak, Tea, Rubber and Coffee). All the coffee and tea samples were classified as ‘shrubs’ and all the bigger plants such as eucalyptus, pine, rubber, and silver-oak were classified as ‘trees.’ These multiple groupings of samples enable us to understand which criteria amongst the above-mentioned groupings best describe the variations in bacterial diversity and composition across the samples. Such multiple groupings were previously used to study the bacterial diversities across the samples (Štempelová et al., 2025; Ramos et al., 2025).

**Figure 4:**
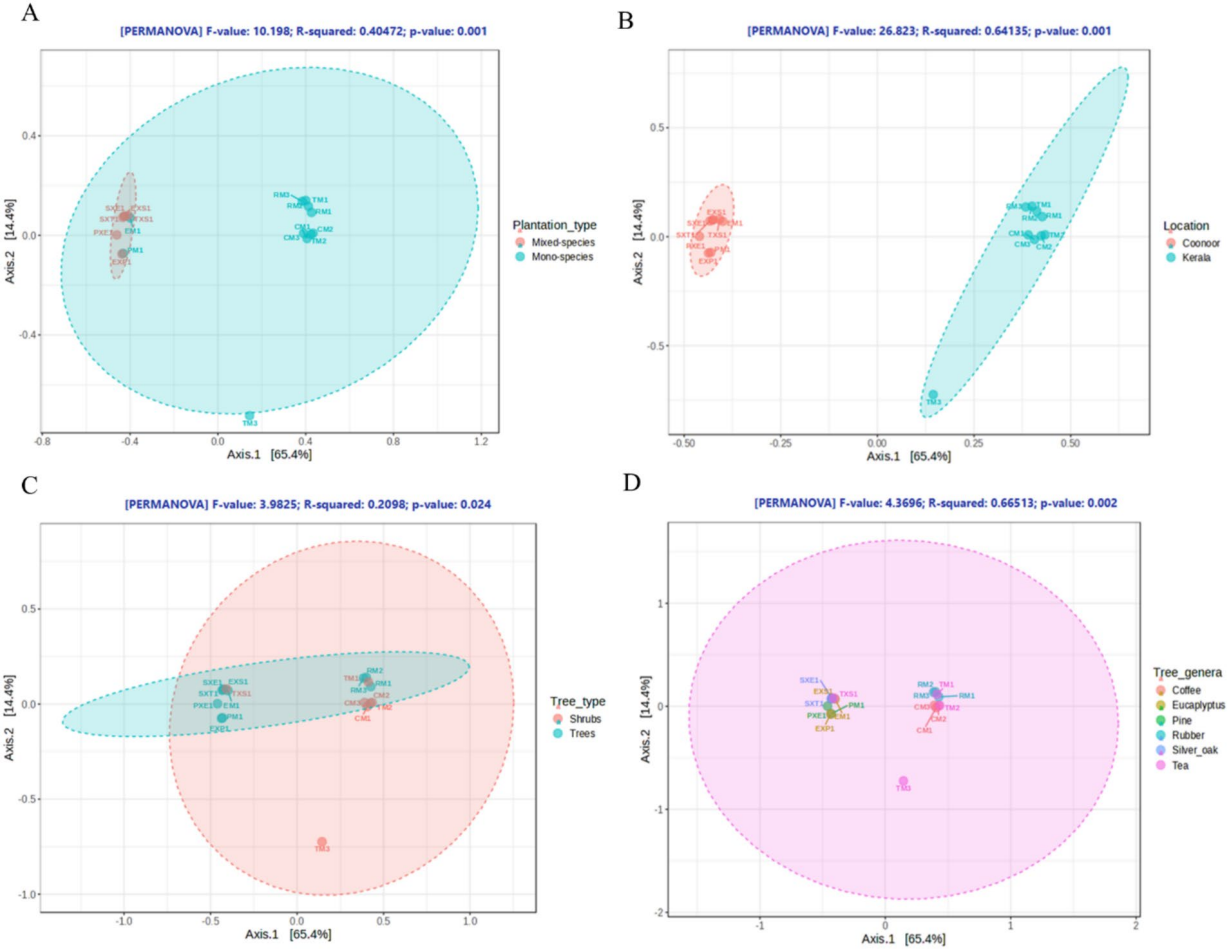
Beta-diversity across the samples is depicted in a PCoA plot based on the Bray–Curtis dissimilarity index of the microbial communities within each group, as shown for **(A)** plantation types, **(B)** sampling locations, **(C)** plant types, and **(D)** plant genera.

The alpha diversity indices such as the Chao1, Shannon, observed, and Simpson indices were not statistically significant (*p* > 0.05) across any of the groupings, and were consistent across the samples suggesting that similar diversity exists across the samples (Supplementary Table S6). However, the alpha diversity indices were lower in TM3, EXP1 and PM1. The alpha diversity plots based on the Shannon diversity index (*p* > 0.05) depicts the lesser diversity of the TM3 (1.386287), EXP1 (2.624097) and PM1 (2.664024) samples. Similarly, PXE1 (3.187136) had comparatively lesser diversity than EM1 (3.888166) and the other samples (Supplementary Figure S7). The *β*-diversity metric was used to identify differences in the bacterial community profiles between the soil samples. The βdiversity acrossthe soil samples were analysed with the principal coordinate analysis (PCoA) plots using the permutational multivariate analysis of variance (PERMANOVA) algorithm on the basis of Bray–Curtis dissimilarity (Figure 4), which revealed that the soil samples clustered separately because of their distinct bacteriome profiles. Beta diversities were statistically significant across all four grouping criteria, revealing differential bacterial diversities across the sample groups. The sample groupings based on ‘plantation type’ and ‘sampling location’ presented greater significance, with a *p*-value of 0.001 in both cases, followed by ‘plant genera’ (0.002) and ‘plant type’ (0.024), basedgroupings. Hence, greater variations in bacterial diversity are observed across plantation types and sampling locations than across plant genera and plant types.

### 3.4 Rhizosphere bacteriome composition

**Figure 5:**
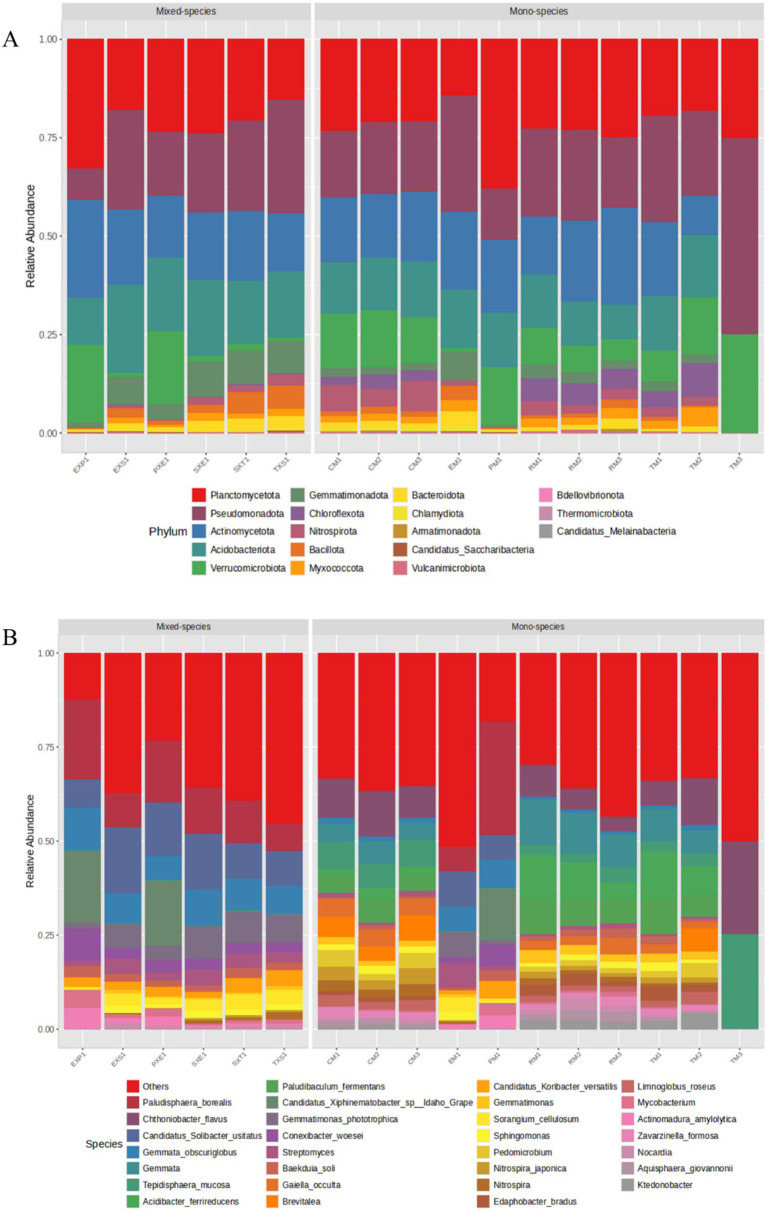
Stacked bar plots depicting the relative abundances of bacterial taxa across the samples, constructed using MicrobiomeAnalyst 2.0. **(A)** phylum-level **(B)** species-level.

Planctomycetota (22.61%) and Pseudomonadota (20.12%) were the predominant phyla detected across the rhizosphere samples, followed by Actinomycetota (18.15%), Acidobacteriota (14.37%), Verrucomicrobiota (9.36%), Gemmatimonadota (3.61%), Chloroflexota (2.78%), Nitrospirota (2.7%), Bacillota (2.03%), Bacteroidota (1.62%), and Myxococcota (1.58%) (Figure 5A). Moreover, earlier reports have shown that, the dominant phyla detected in the present study, especially Pseudomonadota, Planctomycetota, Actinomycetota, and Acidobacteriota are known to include several genera of bacteria which have crucial roles in carbon cycling, phosphate-solubilisation, nitrogen-fixation, and plant growth-promotion (Bruto et al., 2014; Kovaleva et al., 2015; Mathur and Ulanova, 2023; Kalam et al., 2020). Planctomycetota was abundant in the PM1, EXP1 and PXE1 samples, whereas Pseudomonadota dominated the EM1, EXS1, TXS1, and SXT1 samples. Acidobacteriota had a relatively high abundance in the EXS1, SXE1, and EM1 samples. Compared with the other samples, in the PM1, PXE1, and EXP1 samples, Verrucomicrobiota had a significantly greater abundance, whereas Gemmatimonadota had a lower abundance. The other abundant phyla found across the samples were Bacillota and Chloroflexota.

Bacillota had higher abundances in the SXT1 and TXS1 samples. Planctomycetota, Pseudomonadota, and Verrucomicrobiota were the only bacterial phyla abundant in the TM3 sample. Compared with the tea mono-species samples, the coffee and rubber mono-species samples presented a greater abundance of Planctomycetota and a lower abundance of Pseudomonodata. TM3 had a unique microbial composition, which was distinct from that of all the other samples, including TM1 and TM2. Amongst the tea mono-species samples, TM2 had a lower abundance of Actinomycetota and higher abundances of Verrucomicrobiota, Acidobacteriota, and Chloroflexota. Similarly, amongst the rubber samples, RM3 presented a higher prevalence of Actinomycetota, Bacillota, Myxococcota, Bacteroidota and Chlamydiota.

At the class level, approximately 62% of the OTUs belonged to the classes such as; Planctomycetia (19.84%), Alphaproteobacteria (10.85%), Spartobacteria (7.92%), Actinomycetes (7.22%), Acidobacteriia (5.77%), Terriglobia (5.46%) and Gammaproteobacteria (5.25%) (Supplementary Figure S8A). Planctomycetia, Spartobacteria, and Thermoleophilia were the major bacterial classes predominant in the PM1, EXP1, and PXE1 samples. In contrast, Acidobacteriota, Alphaproteobacteria, Deltaproteobacteria, and Gemmatimonadetes were prevalent in EM1, EXS1, SXE1, SXT1, and TXS1. Rubrobacteria and Blastocatellia had comparatively higher abundances in the Kerala samples than in the Coonoor samples. Gemmatales (11.72%), Bryobacterales (8.12%), Isosphaerales (7.85%), Chthoniobacterales (5.11%), Hyphomicrobiales (5.04%), Solirubrobacterales (4.99%), Gemmatimonadales (3.61%), Tepidisphaerales (2.75%), and Nitrospirales (2.71%) together composed approximately 51% of the OTUs at the order level (Supplementary Figure S8B). Tepidisphaerales was more abundant in the coffee samples than in the other samples. At the family level, Gemmataceae (11.72%), Isosphaeraceae (7.85%), Chthoniobacteraceae (5.11%), Solibacteraceae (4.4%), Bryobacteraceae (3.71%), Gemmatimonadaceae (3.61%), Acidobacteriaceae (3.12%), Hyphomicrobiaceae (2.92%) and Tepidisphaeraceae (2.75%) were abundant across the rhizospheric soil samples (Supplementary Figure S9A).

At the genus level (Supplementary Figure S9B), *Gemmata* (8.47%), *Paludisphaera* (5.91%), *Chthoniobacter* (5.11%), *Candidatus Solibacter* (4.4%), *Acidibacter* (3.7%), *Paludibaculum* (3.67%), *Gemmatimonas* (3.55%), *Candidatus Xiphinematobacter* (2.82%), *Tepidisphaera* (2.75%), *Nitrospira* (2.71%), *Conexibacter* (2.26%), *Gaiella* (2.02%), *Streptomyces* (1.87%), *Brevitalea* (1.8%), and *Baekduia* (1.69%) were predominant in the rhizospheric soil samples. The other abundant bacteria were *Pedomicrobium* (1.48%), *Edaphobacter* (1.38%), *Sphingomonas* (1.35%), *Limnoglobus* (1.29%), *Candidatus Koribacter* (1.17%), *Aquisphaera* (1.13%), *Za*var*zinella* (1.12%), *Actinomadura* (1.07%), *Sorangium* (1.06%), *Phenylobacterium* (1.02%), *Nocardia* (1.01%), *Mycobacterium* (0.95%), *Ktedonobacter* (0.94%), *Reyranella* (0.94%), and *Urbifossiella* (0.79%). However, *Candidatus Solibacter*, *Candidatus Xiphinematobacter*, *Candidatus Koribacter*, and *Sorangium* were found only in the Coonoor samples. *Acidibacter*, *Aquisphaera*, *Brevitalea*, *Chthoniobacter*, *Edaphobacter*, *Gaiella*, *Ktedonobacter*, *Limnoglobus*, *Paludibaculum*, *Pedomicrobium*, *Reyranella*, *Tepidisphaera*, and *Zavarzinella* were detected only in the samples from Kerala. At the species level (Figure 5B), *Paludisphaera borealis* (5.88%), *Chthoniobacter flavus* (5.11%), *Gemmata* (4.54%), *Candidatus Solibacter usitatus* (4.4%), *Gemmata obscuriglobus* (3.9%), *Acidibacter ferrireducens* (3.7%), *Paludibaculum fermentans* (3.67%), *Candidatus Xiphinematobacter* sp. Idaho Grape (2.81%), *Tepidisphaera mucosa* (2.75%), *Gemmatimonas phototrophica* (2.14%), *Gaiella occulta* (2.02%), *Brevitalea* (1.8%), *Streptomyces* (1.72%), *Conexibacter woesei* (1.69%), *Baekduia soli* (1.69%), *Pedomicrobium* (1.48%), *Nitrospira japonica* (1.4%), *Gemmatimonas* (1.4%), *Sphingomonas* (1.32%), and *Limnoglobus roseus* (1.28%) were the dominant bacterial species present across the samples, despite significant inter-sample variance.

The cluster dendrogram based on the species-level bacterial composition (Supplementary Figure S10) revealed that the rhizosphere soil samples were grouped into two main clusters: thesamples from Coonoor formed the first cluster, and the samples from Kerala formed the second cluster. Amongst the first cluster, the PM1, PXE1 and EXP1 samples formed a subcluster, and the other five comprised the second subcluster. The second subcluster was divided into two subclusters: TXS1, SXT1, SXE1, and EXS1 formed one subcluster, whereas the EM1 sample alone formed another subcluster. The second cluster, comprising Kerala samples, was broadly divided into two clusters: TM3 alone formed one cluster, and all the other Kerala samples formed another cluster. All three coffee samples and TM2 formed a subcluster, whereas the three rubber samples and TM1 formed another subcluster. The dendrogram revealed that samples collected from the same location and/or from the same plant genus presented greater similarity in terms of bacteriome composition. SXT1 resembles TXS1 (same plot) rather than SXE1 (same species); likewise, EXS1 closely resembles SXE1, suggesting that regardless of the plant genera, the plants in the near vicinity share some common bacterial taxa. The bacteriome compositions of the three coffee samples were similar to one another. The same was the case for the three rubber samples, suggesting that both the same genera and the different sampling locations led to the strong similarity of these samples. However, the compositions of the three mono-species tea samples collected from Kerala were not very similar to one another, especially in the case of TM3.

### 3.5 Core microbiome

The core microbiome analysis revealed that Planctomycetota, Pseudomonadota, Actinomycetota, and Acidobacteriota were the core bacterial taxa with the highest prevalence. Verrucomicrobiota, Gemmatimonadota, Nitrospirota, Chloroflexota, Bacillota, Bacteroidota and Myxococcota were the other prevalent phyla representing the core bacteriome (Supplementary Figure S11A). The groupwise comparison of the core bacteriome revealed varying prevalences of core phyla across various groups. The plant genera based grouping (Supplementary Figure S11B) shows that coffee samples had a greater prevalence of all core phyla except Chlamydiota. Meanwhile, Chlamydiota was prevalent only in the Eucalyptus samples. Chloroflexota and Myxococcota were less prevalent in silver oak samples. Moreover, Bacillota and Bacteroidota were less prevalent in rubber samples. The lower prevalences of Nitrospirota, Chloroflexota, Bacteroidota and Myxococcota were observed in the pine samples. In both the mono- and mixed-species samples, Planctomycetota and Pseudomonadota had the highest prevalences (Supplementary Figure S11C). However, Actinomycetota, Acidobacteriota, Gemmatimonadota, and Bacillota were more prevalent in mixed-species samples than in mono-species samples. Chloroflexota, Verrucomicrobiota, Myxococcota and Nitrospirota were more prevalent in the mono-species samples. Chloroflexota, Verrucomicrobiota, Myxococcota and Nitrospirota had higher prevalence in the Kerala samples (Supplementary Figure S11D). The core bacteriome across the plant types (Supplementary Figure S11E) revealed that Nitrospirota, Chloroflexota, Myxococcota, and Bacteroidota were prevalent in the shrubs, whereas Actinomycetota and Acidobacteriota were prevalent across the tree samples.

The genus-level core microbiome analysis revealed that *Gemmata*, *Gemmatimonas*, *Paludisphaera*, *Candidatus Solibacter*, *Chthoniobacter*, *Tepidisphaera*, *Acidibacter*, *Paludibaculum*, *Nitrospira*, *Conexibacter*, *Streptomyces*, and *Baekudia* were the gene core bacterial genera detected across the samples (Supplementary Figure S12). *Streptomyces*, *Sphingomonas*, *Nitrospira*, *Nocardia*, *Baekudia*, *Conexibacter*, *Gemmata* and *Gemmatimonas* were the predominant genera in bothmixed andmono-species samples (Supplementary Figure S13A). These same genera were predominant in both groups (Kerala and Coonoor) of locations (Supplementary Figure S13B). *Brevitalea*, *Limisphaera*, *Nocardioides*, *Paraconexibacter*, *Usitatibacter*, *Dictyobacter*, *Jatrophihabitans*, and *Solirubrobacter* were the core bacterial genera with relatively high prevalence in the shrub samples but not in the tree samples (Supplementary Figure S13C). Moreover, those that had a relatively high prevalence in only the tree samples were *Acidibervibacterium*, *Acidiferrimicrobium*, *Acidisarcina*, *Actinomadura*, *Bacillus*, *Candidatus Koribacter*, *Candidatus Solibacter*, *Candidatus Xiphinematobacter*, *Lutetalea*, *Mesorhizobium*, *Mycobacterium*, *Paludisphaera*, *Paraburkholderia*, *Phenylobacterium*, *Rhodoplanes*, *Saccharomonosphora*, *Singuisphaera*, and *Sorangium*. The plant genus grouping revealed that the core bacterial genera with the highest prevalence amongst all six plant genera were *Gemmata*, *Gemmatimonas*, *Streptomyces* and *Baekudia* (Supplementary Figure S14). The coffee samples presented a relatively high prevalence of relatively few genera in the core microbiome. However, the tea samples had a greater number of genera with a comparatively lower prevalence.

**Figure 6:**
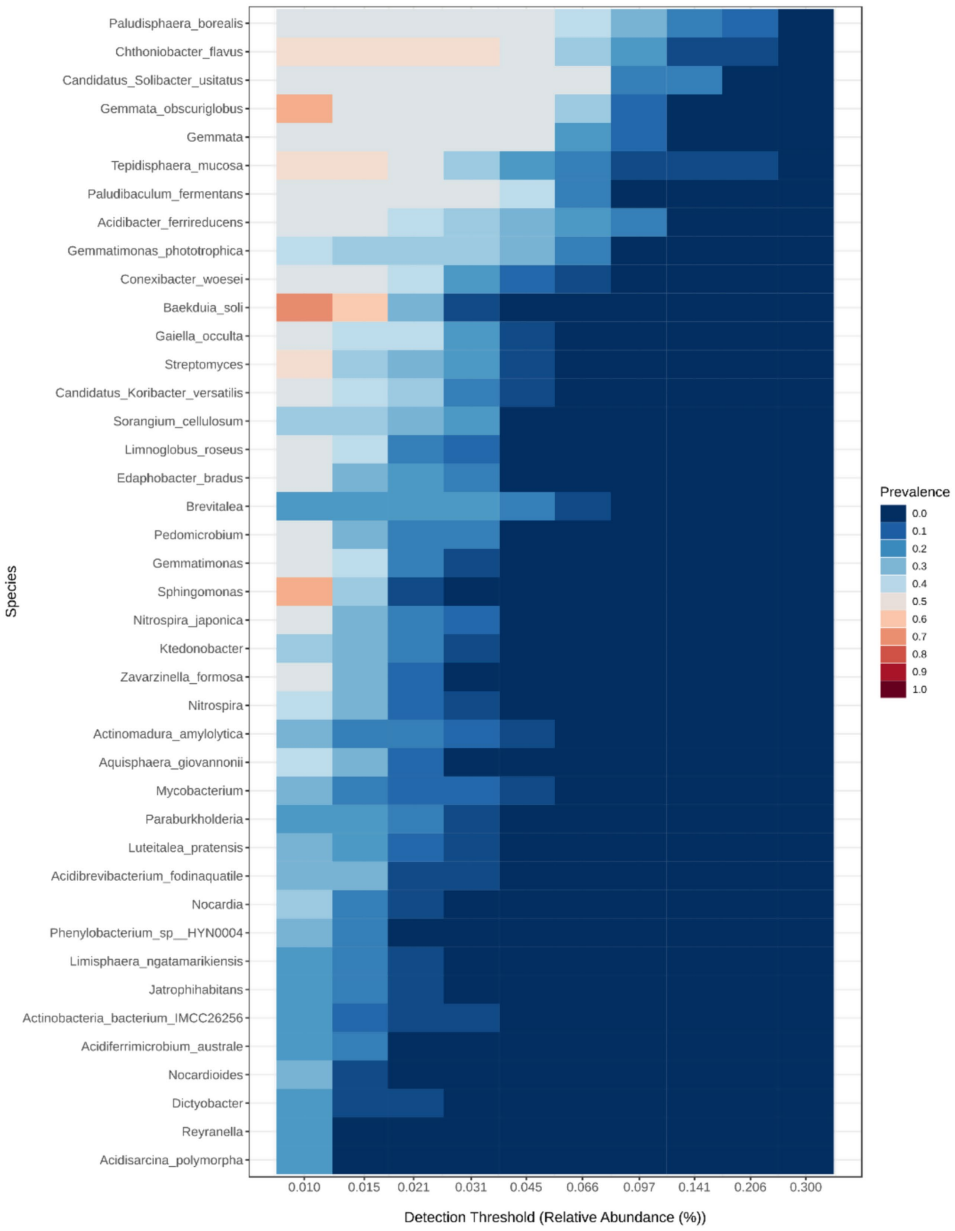
The species-level core bacteriome heatmapdepicting the core bacterial speciesacross the rhizophere soil samples, obtained by applying the parameters of sample prevalence percentage (≥20%) and relative abundance (≥0.01%) (constructed using MicrobiomeAnalyst 2.0).

The species level core bacteriome heatmap (Figure 6) depicts that the core bacterial species were *Paludisphaera borealis*, *Chthoniobacter flavus*, *Gemmata*, *Candidatus Solibacter usitatus*, *Gemmata obscuriglobus*, *Tepidisphaera mucosa*, *Paludibaculum fermentans*, *Gemmatimonas phototrophica*, *Conexibacter woesei*, *Baekduia soli*, *Gaiella occulta Gemmatimonas phototrophica*, and *Streptomyces*. The core microbiome barplots based on plantation types (Supplementary Figure S15A) shows that *Baekduia soli*, *Gemmata obscuriglobus*, *Streptomyces*, *Sphingomonas* and *Nocardia* were the core bacterial taxa common to the mixed-species and mono-species samples. Moreover, these same taxa were detected as the common core bacteria in the ‘Kerala’ and ‘Coonoor’ sample groups, based on the sampling locations (Supplementary Figure S15B). *Baekduia soli* and *Streptomyces*were the only core bacterial taxa found common across all the plant genera (Supplementary Figure S16). *Gemmata obscuriglobus* was not detected in the core microbiome of rubber and *Sphingomonas* was not detected in the core microbiome of pine samples. Whereas, *Nocardia* was absent in the core bacteriome of both pine and silver oak. However, based on the plant types (Supplementary Figure S15C), more number of core bacterial taxa were common to the tree and shrub sample groups.

### 3.6 LEfSe analysis

Linear discriminant analysis (LDA), with effect size (LEfSe) analysis with LDA score > 3 and FDR-adjusted *p*-value < 0.05, was used to identify the key bacterial taxa, that were differentially abundant across the rhizosphere samples (Nath et al., 2023; Imdad et al., 2024). The FDR-adjusted *p*-values were only significant (< 0.05) for the sampling location grouping, at the phylum level. Acidobacteriota and Candidatus Saccharibacteria were predominant in the samples from Coonoor, whereas Chloroflexota and Nitrospirota were predominant in the samples from Kerala (Supplementary Figure S17).

At the genus-level, FDR-adjusted *p*-values were significant only for the plantation types and sampling location based groupings but not significant for the plant genera and plant type groupings. The bacterial genera with significant predominance in the mixed-species samples were*Candidatus Solibacter*, *Candidatus Xiphinematobacter*, *Gemmatimonas*, *Conexibacter*, *Candidatus Koribacter*, *Streptomyces*, *Sorangium*, *Actinomadura*, *Bacillus*, *Paraburkholderia*, *Acidibervibacterium*, and *Paludisphaera*. However, *Chthoniobacter*, *Tepidisphaera*, *Acidibacter*, *Paludibaculum*, *Gaiella*, *Brevitalea*, *Serratia*, *Pseudomonas*, *Pedomicrobium*, *Edaphobacter*, *Limnoglobus*, *Zavarzinella*, *Aquisphaera*, *Ktedonobacter*, *Jatrophihabitans* and *Reyranella* had significantly higher predominance in the mono-species samples (Supplementary Figure S18A). At the same time, based on sampling locations (Supplementary Figure S18B), the bacterial genera with significant predominance in the samples from Coonoor were *Paludisphaera*, *Candidatus Solibacter*, *Candidatus Xiphinematobacter*, *Gemmatimonas*, *Conexibacter*, *Candidatus Koribacter*, *Streptomyces*, *Sorangium*, *Actinomadura*, *Bacillus*, *Paraburkholderia*, *Acidibervibacterium*, *Saccharomonospora* and *Mycobacterium*. On the other hand, *Chthoniobacter*, *Tepidisphaera*, *Acidibacter*, *Paludibaculum*, *Gaiella*, *Brevitalea*, *Serratia*, *Nitrospira*, *Pseudomonas*, *Pedomicrobium*, *Edaphobacter*, *Limnoglobus*, *Zavarzinella*, *Aquisphaera*, *Ktedonobacter*, and *Reyranella* were predominant only in the samples from Kerala.

**Figure 7:**
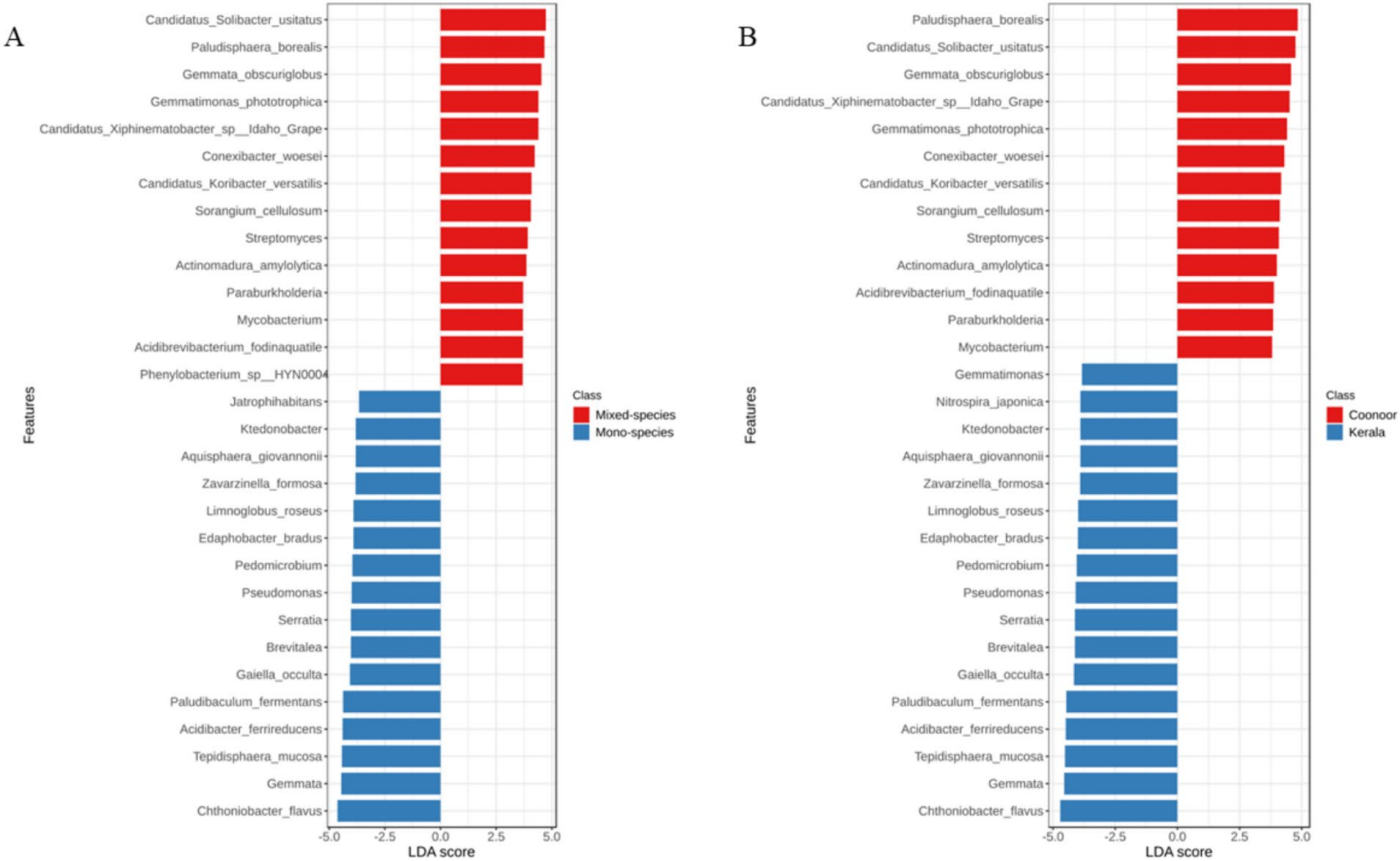
LEfSe analysis major bacterial species across the sample groups, depicting the differentially abundant taxa using LDA scores > 3 and FDR-adjusted *p*-value < 0.05. **(A)** Plantation type **(B)** Sampling location.

Similarly in the species-wise LEfSe analysis also FDR-adjusted *p*-values were significant only for the plantation types and sampling location based groupings (Figure 7). *Paludisphaera borealis*, *Candidatus Solibacter usitatus*, *Gemmata obscuriglobus*, *Candidatus Xiphinematobacter* sp. Idaho Grape, *Gemmatimonas phototrophica*, *Streptomyces*, *Conexibacter woesei*, *Actinomadura amylolytica*, *Paraburkholderia* and *Mycobacterium* were having significantly higher abundance in the ‘mixed-species’ samples (Figure 7A) as well as in the ‘Coonoor’ samples (Figure 7B). *Phenylobacterium* sp. HYN0004 was abundant only in the mixed-species samples. *Chthoniobacter flavus*, *Gemmata*, *Tepidisphaera mucosa*, *Acidibacter ferrireducens*, *Paludibaculum fermentans*, *Gaiella occulta*, *Brevitalea*, *Pedomicrobium*,and *Limnoglobus roseus* had significantly higher abundance in the ‘mono-species’ samples (Figure 7A) as well as in the ‘Kerala’ samples (Figure 7B). *Jatrophihabitans* was abundant only in the mono-species samples. Whereas, *Nitrospira japonica* and *Gemmatimonas* were abundant only in the samples from Kerala.

### 3.7 Taxonomy-to-phenotype mapping

The taxonomy-to-phenotype mapping (Supplementary Figures S19, S20) revealed the ‘Ammonia oxidizer,’ nitrite reducer, sulphate reducer, dehalogenation, chitin degradation, xylan degrader, nitrogen fixation, ‘sulfur metabolizing,’ and lignin degrader were the major metabolism categories assigned. Additionally, a major percentage of the mapping data were classified as unknown (56–81%). In terms of plantation type (Supplementary Figures S19A,B), a lower percentage of mappings were assigned as ‘unknown’ in the mixed-species samples (63.3%), than the mono-species (76.5%). The mixed-species samples had higher abundances of major metabolic functions, such as ‘ammonia oxidizers,’ nitrite reducers, sulphate reducers, dehalogenation, chitin degradation, xylan degraders, nitrogen fixation, ‘sulphur metabolism,’ and lignin degraders. In contrast, dinitrogen fixing (0.02%), gramicidin producer (0.03%), and ‘stores polyhydroxybutyrate (0.5%)’ were detected only in the mono-species samples. The metabolic categories detected in the ‘location’ (Supplementary Figures S19C,D) and ‘plant type’ (Supplementary Figures S19E,F) groupings were similar between the respective sample groups. In terms of the plant genera (Supplementary Figures S20A–F), the metabolic categories with relatively high abundances in the pine samples were sulphate reducers (19.6%), dehalogenation (18.7%), ammonia oxidizers (18.3%) and nitrite reducers (16.4%). In contrast, the ammonium oxidizer was the metabolism category with the higher abundance in the Eucalyptus (30.2%), Silver-oak (35.9%) and Tea (23.6%) samples. Moreover, the nitrate reducer had the highest abundance in the coffee (17.1%) and rubber (15.6%) samples. The ‘Streptomycin producer’ had higher abundances in the Silver-oak (2.5%), Eucalyptus (1.4%), and tea (0.9%) samples than in the pine (0.3%) and rubber (<0.01%) samples.

### 3.8 Functional annotation using PICRUSt analysis

**Figure 8:**
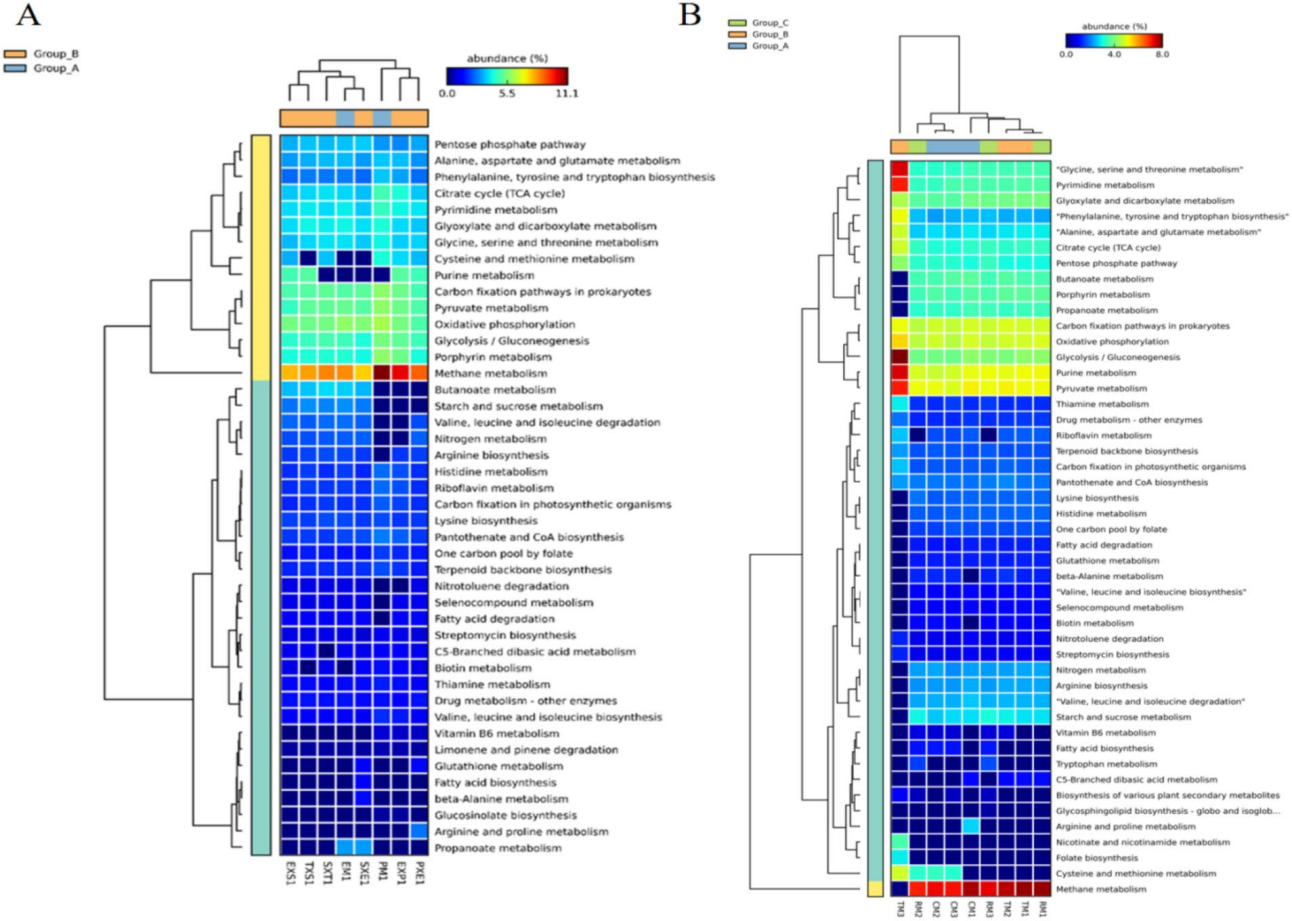
Heatmap showing the functional annotation of genes using PICRUSt analyses. **(A)** Samples from Coonoor **(B)** Samples from Kerala.

The heatmaps (Figure 8) represent the functional annotation of genes belonging to the identified bacteria, which were determined from the Kyoto Encyclopedia of Genes and Genomes (KEGG) pathways using PICRUSt. The functional traits predominant in all the Coonoor samples were methane metabolism, porphyrin metabolism, glycolysis/gluconeogenesis, oxidative phosphorylation, pyruvate metabolism, and carbon fixation pathways in prokaryotes (Figure 8A). The functional traits were broadly clustered into two groups: the first cluster comprised functional traits with relatively high abundances, and the second group comprised functional traits with relatively low abundances. The PM1, PXE1, and EXP1 samples formed separate clusters in the sample dendrogram of the heatmap for the Coonoor samples, whereas the other five samples were clustered together. The citrate cycle (TCA cycle), pyrimidine metabolism, glyoxylate and dicarboxylate metabolism, and glycine, serine and threonine metabolism were abundant in all the samples. Purine metabolism was predominant only in the PXE1, EXP1, EXS1 and TXS1 samples. Butanoate metabolism and starch and sucrose metabolism were significantly lower in the PM1, PXE1 and EXP1 samples. Similarly, nitrogen metabolism was lower in PM1 and EXP1. Terpenoid backbone biosynthesis, streptomycin biosynthesis, glucosinolate biosynthesis, fatty acid biosynthesis, fatty acid degradation, and limonene-pinene degradation were the other important functional traits detected. Glucosinolate biosynthesis was relatively low in the PM1 and EXP1 samples, whereas fatty acid degradation was relatively low in the PM1 samples. However, fatty acid biosynthesis was found to be greater in the SXE1 samples. Carbon fixation pathways in prokaryotes, oxidative phosphorylation, purine metabolism, and pyruvate metabolism were the most abundant functional traits across the Kerala samples (Figure 8B). The Kerala samples were divided into two clusters: TM3 formed a separate cluster, and the remaining 8 samples formed the second cluster. Compared with the other samples, TM3 presented highly variable abundances of functional genes. Methane metabolism had the highest abundance in all the samples except TM3. Moreover, butanoate metabolism, porphyrin metabolism, propanoate metabolism, nitrogen metabolism, arginine metabolism, starch and sucrose metabolism and valine, leucine and isoleucine degradation were relatively low in TM3. The functional traits with higher abundance in TM3 than in the other samples were pyrimidine metabolism, glycolysis/gluconeogenesis, purine metabolism, pyruvate metabolism, folate biosynthesis, glycine, serine and threonine metabolism and nicotinamide metabolism.

**Figure 9:**
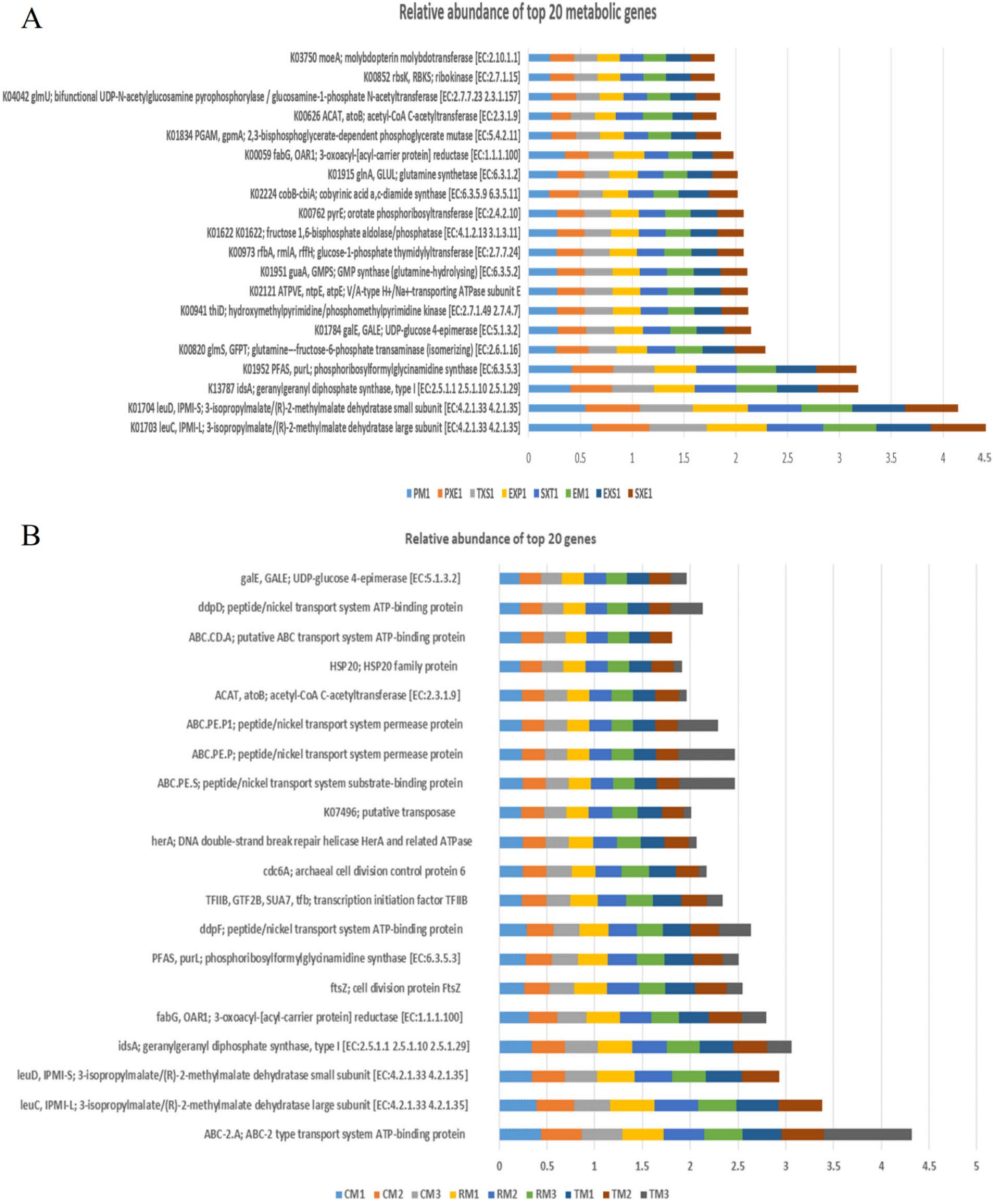
Bar chart depicting the mean relative proportion of the top 20 metabolic genes detected across the samples, using PICRUSt analyses. **(A)** Samples from Coonoor **(B)** Samples from Kerala.

The top 20 metabolic genes detected through the PICRUSt analyses are depicted in Figures 9A,B. [Isopropyl malate/(R)-2-methyl maleate dehydratase large subunit (*leuC*), 3-isopropyl malate/(R)-2-methyl maleate dehydratase small subunit (*leuD*), geranylgeranyl diphosphate synthase, type I gene (*idsA*), phosphoribosyl formyl glycinamide synthase gene (*purL*), and 3-oxoacyl-acyl-carrier protein] reductase (*fabG*)were the metabolic genes with the highest mean relative proportions across all the rhizosphere soil samples. The UDP-glucose-4-epimerase gene (*galE*) and acetyl-CoA C-acetyl transferase (*atoB*) were the other metabolic genes detected amongst the top 20 genes across both the Coonoor and Kerala samples. The glutamine fructose-6-phosphate transaminase gene (*glmS*) and hydroxymethyl pyrimidine/phosphomethylpyrimidine kinase gene (*thiD*) were more abundant in the Coonoor samples.

### 3.9 Correlations between bacteria, metabolites, and soil physiochemical parameters

**Figure 10:**
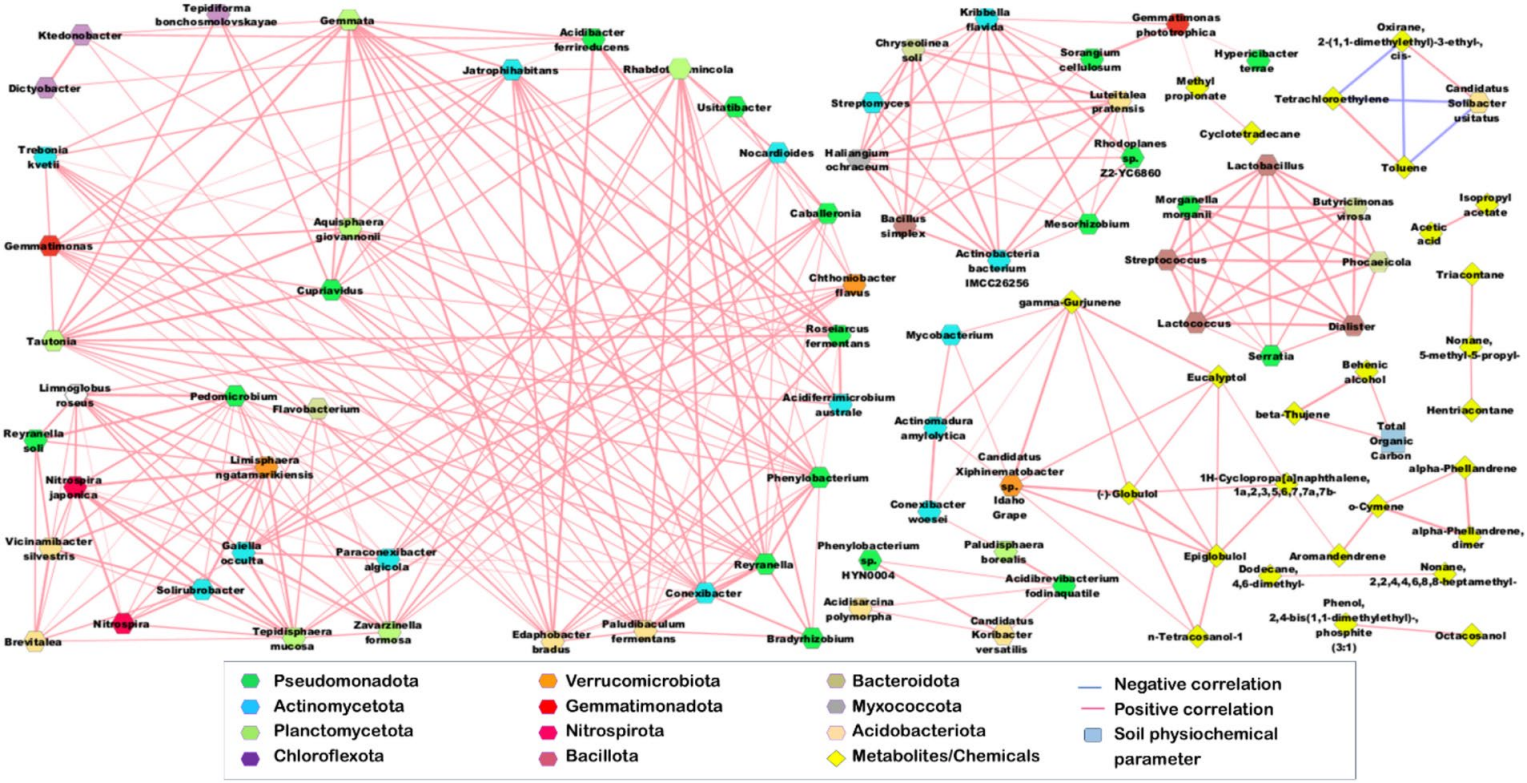
Correlation network (Pearson r) of physiochemical parameters, heavy metal levels and metabolites vs. abundant bacterial taxa generated using the Metscape platform in Cytoscape software (v3.10.2). Significant correlations with r values >0.9 and <−0.9, with *p*-value < 0.05 were only depictedin the network.

The correlation network (Figure 10) illustrates the significant correlations with Pearson’s correlation coefficient (r) greater than 0.9 (0.9 to 1) and less than −0.9 (−0.9 to −1). Moreover, other significant correlations [greater than 0.7 (0.7 to 1) and less than −0.7 (−0.7 to −1)] are given in Supplementary Table S7. Amongst the physicochemical parameters, P had a significant positive correlation with 1-Decanol, 2-hexyl (*r* = 0.79, *p*-value = 0.0001). AN showed significant correlation with *Nocardia* (*r* = 0.82, *p*-value = 0.00006) and *Acidiferrimicrobium australe* (*r* = 0.7, *p*-value = 0.002). Meanwhile, AP was positively correlated with bacterial taxa such as *Streptomyces*, *Luteitalea pratensis*, *Actinobacteria bacterium IMCC26256*, *Hyphomicrobium*, *Rhodoplanes* sp. *Z2-YC6860*, *Haliangium ochraceum*, *Mesorhizobium*, *Chryseolinea soli*, *Legionella*, and *Bacillus simplex*. TOC was positively correlated with *Candidatus Xiphinematobacter* sp. Idaho Grape, and *Actinomadura amylolytica*. Na was positively correlated with *Bacillus*, *Paraburkholderia*, *Sorangium cellulosum*, *Candidatus Koribacter versatilis* and *Gemmatimonas phototrophica*. Cu and Cd were positively correlated with each other and with bacterial taxa, including *Phocaeicola*, *Serratia*, *Lactococcus*, *Morganella morganii*, *Lactobacillus*, *Butyricimonas virosa*, and *Dialister*. The soil DHA had no strong positive correlations with any bacterial taxa or plant metabolites (based on Pearson’s correlation analysis using PAST v4.13). However, it was positively correlated with compounds such as tetrachloroethylene and toluene. Moreover, soil DHA was negatively correlated with bacterial phyla such as *Candidatus Solibacterusitatus*, *Gemmatimonas phototrophica*, *Sorangiumcellulosum*, *Rhodoplanes* sp. Z2-YC6860and *Kribbellaflavida* (Supplementary Table S8). The bacterial taxa exhibited significant positive correlations with each other, particularly between species belonging to the phyla Pseudomonadota, Planctomycetota, Actinomycetota, and Acidobacteriota. *Candidatus Solibacter usitatus* has a positive correlation with oxirane, 2-(1, 1-dimethylethyl)-3-ethyl-, cis-, (*r* = 0.95, *p*-value = 3.26E-09) and has negative correlations with tetrachloroethylene(*r* = −0.94, *p*-value = 1.12E-08) and toluene(*r* = −0.95, *p*-value = 5.80E-09). Even oxirane, 2-(1,1-dimethylethyl)-3-ethyl-,cis-, was also negatively correlated with tetrachloroethylene (*r* = −0.95, *p*-value = 2.72E-09) and toluene(*r* = −0.96, *p*-value = 1.18E-09). Moreover, a significant positive correlation was detected between tetrachloroethylene and toluene (*r* = 0.99, *p*-value = 1.87E-15). At the same time, *Lactobacillus*, *Streptococcus*, *Lactococcus*, *Dialister*, *Morganella morganii*, *Serratia, Butyricimonas virosa*, and *Phocaeicola* were strongly positively correlated with one another. Eucalyptol, epiglobulol,(−)-globulol, gamma-gurjunene, and n-tetracosanol-1 were positively correlated with one another and with the bacterial taxa.

The diterpenoid, Hexadecane, 2,6,10,14-tetramethyl- (phytane) and the carboxylic diester, Hexanedioic acid, bis(2-ethylhexyl) esterhad significant positive correlation with the bacteria *Chthoniobacter flavus*, *Tepidisphaera mucosa*, *Gaiella occulta*, *Brevitalea*, *Limnoglobus roseus*, *Limisphaera ngatamarikiensis*, *Usitatibacter*, and *Reyranella soli*. Meanwhile the isomer of phytane; Hexadecane, 2,6,11,15-tetramethyl- showed significant positive correlation with *Gemmatimonas*, *Ktedonobacter*, *Conexibacter*, *Reyranella Anaeromyxobacter dehalogenans*, and *Bradyrhizobium* The triterpenoid, Friedelan-3-one had strong positive correlation with *Reyranella soli*. At the same time, the terpenoids such as Eucalyptol, gamma-gurjunene, (−)-globulol, and epiglobulol exhibited strong positive correlations with *Paludisphaera borealis* and *Candidatus Xiphinematobacter* sp. Idaho Grape, *Conexibacter woesei*, *Mycobacterium*, *Actinomadura amylolytica*, and *Acidibrevibacterium fodinaquatile*. *Phenylobacterium* sp. HYN0004 was positively correlated with (−)-globuloland epiglobulol. *Paludisphaera borealis* and *Candidatus Xiphinematobacter* sp. Idaho Grape were positively correlated with Aromandendrene. However, o-cymene was positively correlated with *Paludisphaera borealis*. Tetrachloroethylene and toluene were strongly positively correlated with *Paludibaculum fermentans*, *Paraconexibacter algicola*, *Conexibacter*, *Reyranella*, *Usitatibacter*, *Rhabdothermincola* and *Bradyrhizobium*.

## 4 Discussion

### 4.1 Soil physiochemical parameters, heavy metal concentrations, and DHA across the samples

The soil dehydrogenase activity, soil pH, and concentrations of iron, aluminium, cadmium, potassium, lead, copper, chromium, and nickel exhibited varied profiles amongst the rhizosphere samples. The soil physicochemical characteristics and heavy metal concentrations clearly differed between mono-species and mixed-species plantation rhizosphere soils. The mono-species plantations have higher concentrations of K and heavy metals (Pb, Cr, Ni, and Cu) as compared to the mixed species as per our study. At the same time, the mono-species tea sample (TM3) had the lowest pH and the highest concentrations of heavy metals (mainly Cu, Cr, and Cd). Hence, the plantation age and historical land use may not play a significant role in the variations in the concentrations of K and heavy metals (Pb, Cr, Ni, and Cu) across the sites, but the use of fertilisers in tea, coffee and rubber plantations may be a strong probable reason. Earlier reports have shown that compared with the mono-species plantations, mixed-species plantations enhance the nutrient effectiveness and improve the physicochemical characteristics of the soil (Li et al., 2024). Our soil samples had similar pH values to those of the forest soils from the Western Ghats regions of Maharashtra (ranging from 6.8 to 7.1) (Ghare et al., 2023). At the same time, previous studies have found that plant species significantly influence the levels of total organic carbon (TOC), total nitrogen, total phosphorus, and available phosphorus in the soil, primarily through root secretions and litter decomposition. TOC, nitrogen, phosphorus, and available phosphorus are significant indicators of soil fertility and quality (Lemanowicz et al., 2020). Earlier studies reported that K is a common component added in the multicomponent fertilisers such as NPK and PK fertilisers (Gambuś and Wieczorek, 2012), which may have led to the higher concentrations of K in these soils. Earlier reports have shown that, compared with mono-species plantations, mixed-species plantations enhance nutrient effectiveness and improve the physicochemical characteristics of the soil (Lei et al., 2023; Li et al., 2024). The PXE1 and EXP1 samples were rich in TOC content compared to other mono-species as well as mixed-species samples. These two mixed-species samples were found to have higher component areas of several terpenoids and fatty alcohols, including behenic alcohol, n-tetracosanol-1, gamma-gurjunene, eucalyptol, docosanal, (−)-globulol, epiglobulol and aromandendrene (Figure 3). At the same time, the terpenoids such as fenchol and alpha-santalene were detected only in the PXE1 and EXP1 samples (Supplementary Figure S3). Earlier studies reported that the mixed plantations of coniferous trees such as pine, with the broad-leaved trees (angiosperms) led to the increased aromatic and aliphatic compounds in the rhizosphere soils (Huang et al., 2022). Pine plantations were reported to possess the highest soil organic carbon content amongst the tree plantations across the Western Ghats region (Babu et al., 2023). The pine metabolites may have crucial contributions to these higher carbon contents observed in the pine rhizospheres. The combined input of pine and eucalyptus metabolites may be one of the reasons for the observed high TOC content in EXP1 and PXE1. Another possible cause of higher TOC content may be litter accumulation. Earlier studies reported that pine plantations experience a higher rate of litterfall and lead to needle accumulation (Zinn et al., 2002). The pine needles accumulate faster due to the slower decomposing rate caused by the higher lignin: nitrogen ratio compared to other leaves (sclerophyllous or broad-leaves from angiosperms) (Williams and Wardle, 2007). The pine needles possess high lignin (43%) and cellulose (20%) content (Wang et al., 2024); therefore, the decomposition of these accumulated pine needles, along with the litterfall from the eucalyptus trees, will lead to the release of large amounts of organic carbon into the surrounding soil. Hence, the higher TOC content of PXE1 and EXP1 may be attributed to the synergetic effect of pine and eucalyptus root exudationand litter decomposition. However, further studies with deeper insights into the litter decomposition and root exudates composition may be required to validate these observations.

Soil dehydrogenase activity (DHA) is a significant bioindicator of soil fertility and directly resembles microbial activity in the soil (Navnage et al., 2018). The soil DHA of the forest samples was significantly correlated with the pH of the soil samples; slightly acidic samples presented relatively high soil DHA values (Januszek et al., 2015). Muhlbachova et al. (2015) reported that increased concentrations of Zn and Al are significantly negatively correlated with soil dehydrogenase activity. The higher concentrations of Zn in the rhizosphere samples from Coonoor may be a reason for the lower soil DHA in these samples. Moreover, the higher soil DHA in mono-species samples from the Kerala region may have arisen due to the slightly acidic soil pH in these samples, compared with the neutral pH of the samples from the Coonoor region. The soil DHA was significantly negatively correlated with the soil pH, Na, Zn, Al, and Fe. However, positive correlations were found with Pb, Cu, and Cr (Supplementary Figure S2).

### 4.2 The role of major metabolites in the plant rhizosphere

Sesquiterpenoids, monoterpenoids, triterpenes, alkanes, branched alkanes, carboxylic acid esters, fatty alcohols, and aromatic hydrocarbons were the major groups of compounds detected across the samples from the present study. Sesquiterpenes are important plant secondary metabolites that contribute to plant health, defence, and stress tolerance (Barreto et al., 2021). Moreover, terpenoids are crucial plant metabolites in plant adaptations and interactions with biotic and abiotic environmental factors (Chou et al., 2023). Earlier studies have confirmed the phytotoxic activities of terpenoids belonging to the sesquiterpene (Araniti et al., 2016) and monoterpene (Chaimovitsh et al., 2010) classes. These phytotoxic activities of terpenoids enable plants to outcompete their competitors and secure the necessary space and resources for growth and development. Several monoterpenes can inhibit nitrogen mineralisation and net nitrification (Chomel et al., 2016). However, no proteins or carbohydrates were detected. Earlier reports suggest that in many studies attempting to determine root exudate compositions, carbohydrates and proteins were not detected, primarily because of their rapid degradation and microbial uptake (van Dam and Bouwmeester, 2016; Adedeji and Babalola, 2020). Root exudates promote the growth of beneficial microbes and suppress the growth of pathogens (Chai and Schachtman, 2022). Plants are known to select specific bacterial species through various root exudates or chemoattractant compounds (Adedeji and Babalola, 2020). Organic acids, sugars, and amino acids are the major chemo-attractants in root exudates (Feng et al., 2021).

‘Alpha-phellandrene’ is a monoterpenoid reported as a major metabolite in the *Eucalyptus* essential oils (Radice et al., 2022). Alpha-phellandrene exhibits pest-repellent properties and has been shown to possess inhibitory activities against certain bacteria and fungi (Radice et al., 2022). Hence, the presence of monoterpenoids such as alpha-phellandrene (present in all eight rhizosphere samples from Coonoor) suggests that they may serve as a defence mechanism for these plants to repel their microbial and insect pathogens. Eucalyptol is a bicyclic terpenoid found in eucalyptus and tea plants (Mączka et al., 2021). Friedelan-3-one is a triterpenoid detected in various tropical plants and exhibits antimicrobial activity against certain pathogenic bacteria (Chama et al., 2023). 2, 4-Di-tert-butylphenol plays a crucial role in the plant rhizosphere, as it acts as both a chemoattractant and an allelochemical (Lin et al., 2022). 2, 4-Di-tert-butylphenol is an allelochemical detected in plant rhizospheres that can inhibit seed germination and affect the immune system of plants. However, this compound can inhibit pathogens and provide systemic acquired resistance to plants (Zheng et al., 2018). Hence, the presence of cyclohexane, 1, 4-dimethyl-, cis- in the rhizosphere samples from the plot with tea plants (SXT1 and TXS1) may be due to the presence of this metabolite in the tea root exudates. Organic acids in root exudates are strongly associated with the potassium concentration in soils, and formic acid has the strongest positive correlation with available phosphorus (Xu et al., 2021). Acetic acid contributes to alleviating abiotic stress tolerance in plants, such as that caused by salinity, drought, and metal toxicity, by increasing the abundance of soil microbes (Rahman et al., 2024). Alpha-pinene, alpha-phellandrene, camphene, and limonene were detected in the essential extracted from needles, twigs, and branches of the gymnosperm species *Abies alba* (European silver fir) (Ancuceanu et al., 2023); interestingly, the levels of these metabolites were also high in the pine rhizosphere (the only gymnosperm plant in our samples). Cyclohexane, 1, 4-dimethyl- cis- was reported in the tea seeds of *Camellia oleifera* Abel (Zhang et al., 2019). Alkanes serve as the primary energy and carbon source for microbes, leading to the hydrolysis of alkanes into their respective alcohols and fatty acids (Beier et al., 2014). Root exudates, such as carboxylates, facilitate phosphorus solubilisation and provide tolerance to Al toxicity in plants (Chai and Schachtman, 2022). Stigmasterol has been experimentally proven to increase the denitrification enzyme nitrite reductase (NIR) in the bacteria (Lu et al., 2021); hence, stigmasterol may regulate nitrate reduction in soil microbes and maintain the denitrification rate in the soil rhizosphere. The phytochemicals released by plant roots, including terpenes, can regulate the composition of the rhizosphere microbiome and influence nutrient availability. Therefore, the phytochemical diversity of the rhizosphere affects both the rhizosphere microbiome and the ecosystem (Müller and Junker, 2022).

The plant metabolites (or metabolite classes) detected were previously reported to be involved in various ecological functions within the plant rhizosphere, such as allelopathic interactions (positive or negative plant–plant interactions), attracting beneficial microbes to the rhizosphere zone, and repelling pathogenic microbes and pests. Our results suggest that the major metabolites detected in the present study enable the healthy survival of plants by interacting with various biotic and abiotic factors.

### 4.3 Role of varying bacteria across the rhizosphere soils of the Western Ghats

Pseudomonadota, Acidobacteriota, Actinomycetota, Planctomycetota, Verrucomicrobiota, Gemmatimonadota, Nitrospirota and Bacillota were the core bacterial phyla detected with relatively high prevalence across the rhizosphere samples of the present study. Earlier studies have shown that Acidobacteriota, Actinomycetota, and Pseudomonadota are more abundant in several forest soils, indicating their ecological importance (Lladó et al., 2017). Pseudomonadota, Acidobacteriota, Actinomycetota, Verrucomicrobiota, and Bacteroidota are predominant in the Sal tree (*Shorea*) rhizospheric soils of the Joypur tropical forest in West Bengal (Bera et al., 2023). The dominant bacterial taxa found in Eucalyptus rhizosphere soils from China are Pseudomonadota, Acidobacteriota, Actinomycetota, and Chloroflexota (Qu et al., 2020; Huo et al., 2024). The abundant phyla in the tropical coffee rhizospheres from the State of São Paulo, Brazil, were Pseudomonadota, Acidobacteriota, Actinomycetota, Planctomycetota, Verrucomicrobiota, Gemmatimonadota, Nitrospirota, Bacillota, and Bacteroidota (Andrade et al., 2023), which are similar to the phyla found in the coffee samples from the present study. The rhizosphere bacteriomes of rubber trees from tropical regions of China are composed of Pseudomonadota, Actinomycetota, Acidobacteriota, Chloroflexota, Bacteroidota, Gemmatimonadota, Planctomycetota, Myxococcota and Verrucomicrobiota (Lan et al., 2023). However, Chloroflexota and Myxococcota were not abundant in these samples but were found in the samples of the present study. Various Pseudomonadota, Bacillota and Actinomycetota genera are known for degrading lignin and other plant-produced phenolic compounds (Lladó et al., 2017). The phylum Acidobacteriota comprises several bacteria that are tolerant to acidity, stress, and starvation; play crucial roles in carbon, nitrogen, and sulphur metabolism; and promote plant growth (Kalam et al., 2020). Members of Bacillota and Actinomycetota promote plant growth by producing various polysaccharides, lipopeptides, aromatic compounds, and plant hormones (Mathur and Ulanova, 2023). Gemmatimonadota species are reported to be common in soils, plants, and rhizospheres (Mujakić et al., 2022).

Amongst the samples from Coonoor, PM1, EXP1, and PXE1 differed from the remaining samples because of the relatively high abundances of *Plaudisphaera borealis*, *Candidatus Xiphinematobacter* sp—Idaho Grape, and *Actinomadura amylolytica*. Moreover, *Streptomyces* sp., *Sorangium cellulosum*, and *Gematimonas phototrophica* had relatively high abundances in the EM1, EXS1, SXE1, and SXT1 samples. *Candidatus Solibacter usitatus* and *Gemmata obscuriglobus* were abundant across all the samples from Coonoor. The TM3 sample had higher abundance of *Lactobacillus*, *Streptococcus*, *Lactococcus*, *Dialister*, *Morganella morganii*, *Serratia*, *Butyricimonas virosa*, and *Phocaeicola*, which were not abundant in any other samples. The eucalyptus and silver oak rhizosphere samples presented a greater abundance of *Streptomyces* species than did the pine rhizosphere samples. *Mycobacterium*, *Streptomyces*, *Bacillus*, *Conexibacter*, *Sphingomonas*, and *Phenylobacterium* were previously detected as the major endophytic bacterial genera in Eucalyptus root samples collected from Rio de Janeiro State, Brazil (Fonseca et al., 2018). The coffee rhizospheres of tropical Brazil also presented relatively high abundances of *Streptomyces*, *Sphingomonas* (de Sousa et al., 2022), *Reyranella*, *and Nocardioides* (Andrade et al., 2023). The dominant genera detected in the rhizospheric samples of Eucalyptus plantations in China were *Ktedonobacter*, *Burkholderia*, *Mycobacterium* and *Gaiella* (Qu et al., 2020). *Paraburkholderia* were reported to be less abundant in pine rhizospheric soils in Poland (Staszel-Szlachta et al., 2024). On the other hand, some abundant bacteria detected in the present study have not yet been reported in rhizosphere soils from tropical regions of India; for example, *Paludisphaera borealis* is a hydrolytic planctomycete that was previously detected in Western Himalayan soils and wetlands (Kulichevskaya et al., 2016). *Candidatus Koribacter*, *Candidatus Solibacter*, and *Chthoniobacter* are the prevalent genera in indigenous and commercial forests in South Africa (Amoo et al., 2019). *Edaphobacter bradus* was isolated from the forest soil of a biosphere reserve in China (Xia et al., 2018). *Actinomadura amylolytica* was isolated from soils collected from geothermally heated regions of Yunnan Province, China (Jiao et al., 2015). *Reyranella soli* was first isolated from the forest soils of Korea (Kim et al., 2013). *Lactococcus* is abundant in rhizosphere soils from alpine wet meadows (Iqbal et al., 2024) and has also been isolated from non-rhizosphere soils of black plum (Sharma et al., 2021). *Streptococcus* is abundant in *Lonicera japonica* rhizosphere soil (Wang et al., 2023). *Morganella morganii* is reported to be an opportunistic pathogen (Liu et al., 2016) and was found to possess zinc sequestration properties, especially for strains isolated from polluted soils (Ramya et al., 2022). *Lactobacillus* strains were isolated from soil samples collected from Korea (Kim et al., 2018), were detected in the rhizospheres of *Phytolacca* species and had significant interactions with the soil pH and Cd concentration. *Lactobacillus* species can reduce the soil pH and increase Cd uptake by plants (Li et al., 2023). *Reyranella soli* has been reported as a plant growth-promoting bacterium that helps plants take up nutrients (Li et al., 2022; Zhang et al., 2023b). *Streptomyces* species play crucial roles in promoting plant growth and suppressing pathogens (Himaman et al., 2016). *Gemmata obscuriglobus* is a planctomycete known to produce sterols (Rivas-Marin et al., 2019). *Gemmatimonas phototrophica* are reported as facultative photoheterotrophs capable of anoxygenic photosynthesis and requires organic substrates. *G*. *Phototrophica* produces several carotenoids and bacteriochlorophyll a (Mujakić et al., 2022). *Gaiella occulta* can be considered an indicator of soil pollutions caused due to polyaromatic hydrocarbons (PAHs) (Zhang et al., 2023a). *Mesorhizobium* is a growth-promoting rhizobacterium capable of copper phytoremediation (Mannaa et al., 2020). *Pseudomonas* species are commonly found in plant rhizospheres and are root-colonising and plant growth-promoting bacteria with the ability to produce siderophores and indole-3-acetic acid (Qessaoui et al., 2019). Although some species of *Serratia* are known as opportunistic pathogens, *Serratia* species also stimulate plant growth and suppress the growth of several soil-borne fungal pathogens (Neupane et al., 2013). *Acidibrevibacterium fodinaquatile* exhibit resistance to heavy metals and tolerance to osmotic stress (Bolivar-Torres et al., 2022). *Acidibacter ferrireducens* is a gamma-proteobacterium capable of ferric iron reduction (Falagán and Johnson, 2014). *Candidatus Solibacter* species serve as bioindicators of soil fertility (Liu et al., 2024). *Solibacillus silvestris* possesses amylolytic properties (Pascon et al., 2011), whereas *Cellulomonas flavigena* is capable of cellulose and xylan degradation by producing cellulases and xylanases (Sánchez-Herrera et al., 2007). *Gemmatimonas* may aid plant growth via phosphate solubilisation (Zhou et al., 2022). The other major bacteria capable of phosphate solubilisation are *Pseudomonas* (Qessaoui et al., 2019), *Streptomyces* (Himaman et al., 2016), *Bacillus* (Jha and Subramanian, 2014; Rathore et al., 2021), *Paraburkholderia*, and *Burkholderia* (Tang et al., 2020). Several bacterial species detected in the present study have been previously identified in the rhizospheres of various tropical plants.

The Pearson correlation analysis revealed that soil physiochemical parameters, including TOC, soil pH, AP, AN, and heavy metals, were the major abiotic factors that were positively correlated with various bacterial taxa (Supplementary Table S7); these positive correlations may indicate biotic–abiotic interactions in the rhizosphere soils. The heavy metals Pb, Zn, Cr, Cu, Cd, Fe, and Al presented significant positive correlations with several bacterial species. Physicochemical properties, such as P, N, K, C, Na, Mg, and Ca contents, are relatively high in rhizospheric soils with increased rhizodeposition and microbial activity due to relatively high amounts of exudates and metabolites from roots and microbes (Kandziora-Ciupa et al., 2022). Available nitrogen (AN), potassium (K), and soil pH affect the soil microbial community composition (Xu et al., 2024). The terpenoids such as phytane, Hexadecane, 2, 6, 11, 15-tetramethyl, and Friedelan-3-one had significant positive correlations with bacteria abundant in the samples collected from Kerala. At the same time, the terpenoids such as Eucalyptol, gamma-gurjunene, (−)-globulol, and epiglobulol exhibited strong positive correlations with bacterial taxa prevalent in the Coonoor samples.

The correlation analysis revealed that tetrachloroethylene and toluene had strong positive correlations with Actinomycetota (*Paraconexibacter algicola*, *Conexibacter*, and *Rhabdothermincola*), Pseudomonadota (*Reyranella*, *Usitatibacter*, and *Bradyrhizobium*), and Acidobacteriota (*Paludibaculum fermentans*). The major toluene-degrading bacteria belong to the phyla Actinomycetota and Pseudomonadota (BenIsrael et al., 2021). Furthermore, *Bradyrhizobium* strains were found to possess toluene monooxygenase (TMO) six-gene clusters, which are responsible for the hydroxylation and degradation of toluene and other aromatic compounds (Yanık-Yıldırım and Vardar-Schara, 2014). Hence, the positive correlations of toluene with these Actinomycetota and Pseudomonadota taxa may have arisen due to the possible toluene-degrading capabilities of these bacteria. *Candidatus Xiphinematobacter* sp. *Idaho Grape*, *Actinomadura amylolytica*, *Paludisphaera borealis*, *Mycobacterium*, *Conexibacter woesei*, and *Acidibervibacterium fodinaquatile* exhibit strong positive correlations with each other and with plant metabolites such as gamma-gurjunene, eucalyptol, (−)-globulol, and epiglobulol. However, various sesquiterpenes and monoterpenoids are known to promote the growth of rhizosphere microbes (Massalha et al., 2017). Earlier reports revealed that terpenoid compounds serve as energy sources for several bacteria. For example, *Serratia* and *Pseudomonas* are capable of terpenoid degradation and can be utilised as carbon sources (Qiao et al., 2023). Similarly, *Burkholderia* and *Pseudomonas* are capable of degrading *β*-pinene and β-caryophyllene (Francoeur et al., 2020). Monoterpenoids, such as alpha-pinene, serve as carbon sources for *Pseudomonas* species (Massalha et al., 2017; Adedeji and Babalola, 2020). *Pseudomonas* and *Rhodococcus* are known to possess terpene-degrading capabilities (Eaton and Sandusky, 2010), and *Pseudomonas putida* can hydrolyse eucalyptol (Mączka et al., 2021). These results suggest that terpenoids with positive correlations with rhizosphere bacteria may play a crucial role in the differential composition of the bacteriome in the rhizosphere. However, further studies may be required to validate the positive interactions between these metabolites and bacteria detected in the present study.

Amongst these bacteria involved in the nitrogen cycle, *Nitrospira japonica* and *Gemmatimonas* were found to be abundant only in the samples from Kerala. *Nitrospira*, *Gemmatimonas*, *Anaeromyxobacter*, *Phenylobacterium* were known for their crucial roles in the nitrogen cycle (Mehmood et al., 2022). *Nitrospira* are nitrate oxidisers and *Rhodoplanes* are denitrifying bacteria (Rosenzweig et al., 2013). Nitrogen-fixing bacteria, such as *Mesorhizobium* (Mannaa et al., 2020) and *Rhizobium* (Li and Li, 2023), were also detected in the samples, especially in the Coonoor samples. *Paludibaculum* is known to participate in the nitrogen cycle through feammox processes (Jia et al., 2025). *Candidatus Koribacter* and *Candidatus Solibacter* have an important role in nitrogen cycling (Ward et al., 2009). *Hyphomicrobium* is known for its methylotrophic and denitrifying capabilities (Rosenzweig et al., 2013; Martineau et al., 2015). *Candidatus Solibacter usitatus* strains play a crucial role in the carbon cycle, as they possess carbohydrate-active enzyme (CAZyme) genes that enable the breakdown and utilisation of various polysaccharides from plants, such as cellulose, hemicellulose, pectin, glycogen, or starch (Rawat et al., 2012). *Chthoniobacter flavus* is a heterotrophic bacterium rich in glucoside hydrolase enzyme (CAZy) that grows by degrading plant biomass polysaccharides and carbohydrates (Sharma et al., 2023). Moreover, *Actinomadura amylolytica* strains are reported to possess extracellular cellulases, including cellulase beta-glucosidase genes, and are known to be efficient cellulose degraders (Yin et al., 2018). *Sorangium cellulosum* strains possess cellulolytic and lipolytic properties, enabling them to efficiently breakdown lipids and polysaccharides (Yuan et al., 2023). Moreover, the genome of *Acidibrevibacterium fodinaquatile* contains genes involved in carbon fixation (Bolivar-Torres et al., 2022). *Paludibaculum fermentans* uses complex polysaccharides as growth substrates (Dedysh et al., 2021). *Serratia* and *Bacillus* possess cellulolytic properties (Tang et al., 2020; Meena et al., 2022). The other major bacterial taxa using carbon sources such as polysaccharides and sugars as their growth substrates were *Tepidisphaera mucosa* (Kovaleva et al., 2015), *Edaphobacter bradus* (Xia et al., 2018) and *Gaiella* (Wei et al., 2024).

Whereas, *Candidatus Koribacter* and *Phenylobacterium* sp. HYN0004 were abundant only in the samples from Coonoor, based on the LEfSe analysis. Previously it was reported that the geographical locations lead to greater variations in the microbial communities of tropical plants than in those of plant taxa (Ji et al., 2024). *Paludibaculum fermentans* and *Reyranella soli* were found only in the core microbiome of mono-species samples. In contrast, *Rhodoplanes* and *Mesorhizobium* were found only in the core microbiome of mixed-species samples. Similarly, amongst the bacteria involved in the carbon cycle, *Candidatus Solibacter usitatus*, *Actinomadura amylolytica*, *Sorangium cellulosum*, and *Acidibrevibacterium fodinaquatile* had significantly higher abundance in the mixed-species samples based on the LEfSe analysis. However, *Chthoniobacter flavus*, *Tepidisphaera mucosa*, *Edaphobacter bradus*, *Gaiella occulta*and *Paludibaculum fermentans* had significantly higher abundance in the mono-species samples. The results of the present study suggest that the occurrence and abundance of biogeochemical cycle-related and ecologically significant bacteria across the Western Ghats are dependent on both plantation type and sampling location.

### 4.4 Predictive functional roles of the rhizosphere bacteriome

The functional predictions revealed that methane metabolism, purine metabolism, porphyrin metabolism, glycolysis/gluconeogenesis, oxidative phosphorylation, pyruvate metabolism, ABC transporters, and carbon fixation pathways in prokaryotes are the major functional traits predicted. Streptomycin biosynthesis, fatty acid biosynthesis and fatty acid degradation were the functional traits reported. The greater abundances of amino acid biosynthesis functional traits confirmed the ability of the bacteriome to produce amino acids (Nwachukwu et al., 2023). *Streptomyces* species are mainly responsible for streptomycin biosynthesis, which can, in turn, enhance plant defence by suppressing plant pathogens and other antagonistic bacteria (Lin et al., 2012; Chaparro et al., 2014). Fatty acids play crucial roles in the survival and stress response of microbes, particularly in acidic environments (Wu et al., 2018; Fozo and Quivey Jr, 2004). Bera et al. (2023) reported that purine metabolism, pyrimidine metabolism, methane metabolism, alanine, aspartate, and glutamate metabolism, ABC transporters, two-component systems, oxidative phosphorylation, and aminoacyl-tRNA biosynthesis were the major functional traits detected in the rhizosphere soil microbiome of Joypur tropical forests in West Bengal.

Although the metagenomics approach cannot directly detect gene expression in the rhizosphere, it can be used to infer gene expression indirectly (Wu et al., 2018). In the present study, the top 20 metabolic genes were predicted via PICRUSt analyses. The *leuC* (3-isopropyl malate/(R)-2-methyl maleate dehydratase large subunit) gene significantly influences the transcript levels of various plant-beneficial genes, such as siderophore biosynthetic genes, iron starvation regulator/adaptor genes, and ferric TAFC uptake transporter genes (Orasch et al., 2019). The geranylgeranyl pyrophosphate synthase gene *idsA* is involved in carotenogenesis in bacteria (Heider et al., 2014). Carotenoids are precursors for vitamin A and plant hormones such as strigolactones and abscisic acid (ABA) (Stra et al., 2023). The *purL* gene has potential for biofilm formation (Cepas et al., 2020), and bacterial biofilms play a crucial role in the rhizosphere, particularly in root adhesion and colonisation (Bhattacharyya et al., 2023; Zhang et al., 2015). The *atoB* gene plays a crucial role in the biosynthesis of secondary fatty acid metabolism and carbon metabolism (Marmitt et al., 2024). The *fabG* gene plays a significant role in fatty acid biosynthesis in bacteria (Shanbhag, 2019; Mao et al., 2016). Fatty acids, including both saturated and unsaturated fatty acids, play crucial roles in plant growth (Aluko et al., 2021; Bonaventure et al., 2003). The *galE* gene synthesises lipopolysaccharide (LPS) (Fry et al., 2000). LPSs play significant roles in plant rhizospheres, as they provide surface adaptations and serve as communication signals in plant–microbe interactions (Ormeño-Orrillo et al., 2008). Hence, the major metabolic genes predicted to have a relatively high mean proportion across the samples contribute to various functions, such as root colonisation, nutrient absorption, and plant growth promotion; thus, they may play crucial roles in the health and survival of the plants and rhizosphere microbiome.

### 4.5 Probable factors responsible for the differential bacterial composition and diversity

Previous studies have also indicated that mixed-species plantations have higher soil nutrients, beneficial microbes, bacterial diversity, and functional potential compared to mono-species plantations (Zuo et al., 2025). Mixed-species plantations exhibit increased bacterial diversity and ecological niches due to the greater heterogeneity and biodiversity of both above-ground and below-ground environments (Qu et al., 2022). Mono-species plantations lead to reduced biodiversity and are prone to disturbances, whereas mixed-species plantations increase multifunctionality, ecosystem services, and resilience (Messier et al., 2022). The LEfSe analysis of our study revealed statistically significant variations in bacterial composition (LDA score > 3; FDR-adjusted *p* value < 0.05) across plantation types and sampling locations. Compared with mono-species plantations, mixed-species plantations offer increased resource heterogeneity. Resource heterogeneity contributes to greater species diversity in ecosystems, and it governs species richness more than the quantity of resources (Zhang et al., 2020). Owing to their heterogeneous tree composition, mixed-species plantations can maintain a more ideal microclimate and reduce the impact of climate change events on plantations (Zhang et al., 2022). Our results showed that the mixed plantations of pine and eucalyptus had a higher peak area for several plant metabolites and TOC contents than did their corresponding mono-species samples did, suggesting that the heterogeneity in the above-ground plantation is also reflected in the below-ground resource availability. These increased metabolites and TOC contents may attract beneficial bacteria to rhizosphere soils, as these samples presented relatively high abundances of *Gemmata*, *Actinomadura*, and *Streptomyces* species. Previous studies have also shown that rhizosphere soil has a higher abundance of beneficial bacteria, including Actinobacteria and Planctomycetes, when a mono-species forest is converted into a mixed-species forest (Lei et al., 2023). The altitude was the major variable across the sampling locations of the present study (Table 1). It was previously reported that altitude was the major geographic parameter affecting the soil bacteriome composition and diversity across the sampling locations in the Western Ghats regions of Maharashtra (Ghare et al., 2023). Hence, altitude may be the major factor responsible for the variations in bacterial composition across the sampling locations. The plantations considered under the present study were known to use tilling only during or before the planting. The plantation plots with mature trees usually follow a no-tillage strategy to prevent the root damage of the trees. Mulching techniques are usually used for controlling the weeds. Hence, tillage is not expected to have a severe impact on the soil microbiome composition of the mature plantations considered in the present study.

The major genera dominating the PM1, PXE1, and EXP1 rhizospheric soils were *Candidatus Xiphinematobacter* sp. Idaho Grape, *Actimomadura amylolytica*, *Paludisphaera borealis*, *Mycobacterium*, *Conexibacter woesei*, *Actimomadura* sp., *Acidobacterium capsulatum* and *Xanthobacter autotrophicus*. Terpenoids including monoterpenes and sesquiterpenoids (probably from pine root exudates) with species-specific differential impacts on microbial taxa may have led to the unique microbial composition of these three samples. Hence, the lower alpha diversity and varied bacterial composition of the EXP1 sample may be due to the influence of pine metabolites, which selected only the particular bacterial species that has affinity for the pine metabolites. However, the functional annotation of genes revealed that, despite the low bacterial diversity in the PM1, PXE1 and EXP1 samples, the ecologically relevant inputs of these rhizosphere soil bacteriomes, such as those related to carbon fixation, metabolism, and nutrient cycling, were not compromised in these rhizospheric samples. Hence, the low bacterial diversity clearly did not affect the abundance of metabolic genes or functional traits of the PM1, PXE1, and EXP1 samples. This finding indicates that the pine trees not only have a highly selective rhizosphere microbiome with reduced diversity but also possess all-important functional gene categories similar to those found in other rhizosphere samples in the present study.

Similarly, the significantly low bacterial diversity and highly variable bacterial composition of the TM3 sample were also noted. Even though the TM1, TM2 and TM3 samples were collected from nearby locations at the same time, the TM3 sample was dominated by *Lactobacillus*, *Streptococcus*, *Lactococcus*, *Dialister*, *Morganella morganii*, *Serratia*, *Butyricimonas virosa*, and *Phocaeicola*, which were not abundant in any other samples, including TM2 and TM1. The TM3 sample had the highest concentrations of Cu, Cr, and Cd. The soil sample was the most acidic. The TM3 sample had some unique compounds present only in this sample, mostly benzoic compounds and chemicals with Br, Cl, Li, F and Si (Supplementary Table S9). The long-term usage of inorganic fertilisers (especially the NPK fertilisers) may lead to decreased soil pH and altered soil microbiome composition (Megyes et al., 2021). The combined effect of these toxic chemicals with lower soil pH and increased concentrations of heavy metals may have led to the altered bacterial composition in the TM3 sample. The presence of these compounds and increased heavy metal concentrations in such plantation soils may have led to the loss of natural diversity in the soil microbiome and altered the soil ecology of the Western Ghats region. The periodic monitoring of the below-ground diversities of commercial plantations should be maintained to ensure the sustainable ecosystem functioning of the Western Ghats regions. However, commercial plantations, except for TM3, have not experienced this type of loss in their soil microbiomes, even in the presence of toluene and tetrachloroethylene in the soil. The findings indicated that ‘plantation types’ and ‘sampling sites’ caused the most significant changes in bacteriome composition and diversity across the samples, followed by ‘plant genera’ and plant type.

The GLM analysis is used to reveal the relatedness between the various factors or parameters (Meira-Neto et al., 2017). Here we employed the GLM analysis to unravel the relationships between various soil parameters affecting the rhizosphere microbiome. Our results (Supplementary Figure S21) indicated an antagonistic relationship between soil pH and DHA. Whereas TOC and AP were positively related. Similarly, Ca vs. Mg and N vs. P analyses also showed positive relatedness. The Na vs. K and NN vs. AN GLM analyses indicated slightly antagonistic relationships. All the GLM analyses across the heavy metals were positively related. Further, the GLM analysis was performed between the soil parameters and Shannon diversity index across the samples (Supplementary Figure S22). The dispersion phi, slope, deviance, G-value and *p*-value of the GLM analyses are provided in the Supplementary Table S10. The analyses revealed that P, AP, Mg and soil pH were the major soil parameters exhibiting a positive relationship with the bacterial diversity. Whereas the heavy metals Cd, Cu, Cr and Zn showed a strong antagonistic relationship (*p*-value ≤ 0.05) with the bacterial diversity, followed by Ni. The results suggest that the P, AP, Mg and soil pH enhanced the bacterial diversity across the rhizosphere samples. At the same time, the heavy metals Cd, Cu, Cr and Zn were the major factors responsible for the reduced bacterial diversity in the rhizosphere samples from the Western Ghats.

## 5 Conclusion

In the present study, we compared the soil physicochemical characteristics, metabolome, and microbiome composition across rhizosphere soil samples from the Western Ghats. Plantation type and sampling location were the factors that significantly influenced the rhizosphere metabolome and microbiome profiles, followed by plant genera and plant type. The mono-species samples from Coonoor clearly differed from the mixed-species plantation samples from this location based on soil physicochemical parameters. The mono-species samples had higher concentrations of K and heavy metals, such as Pb, Cr, Ni, and Cu (especially in TM3), than the mixed-species samples, probably due to the usage of fertilisers in these plantations. The soil dehydrogenase activity was highest in the mono-species tea and coffee samples, probably due to the acidic pH of these samples. The soil dehydrogenase activity was significantly lower in the mono-species eucalyptus (EM1) and pine (PM1) samples when compared to the mixed-species eucalyptus (EXP1) and pine (PXE1) samples. Sesquiterpenoids, monoterpenoids, triterpenes, alkanes, branched alkanes, carboxylic acid esters, fatty alcohols, and aromatic hydrocarbons were the major metabolites and they contibute to plant and ecosystem health through the suppression of pathogens, allelopathic interactions with other plants and enabling plant microbe interactions. The beneficial bacterial taxa comprised the majority of the rhizosphere microbes, suggesting that these bacterial taxa are crucial in the ecological sustainablituy of the Western Ghats. The rhizosphere bacteriome compositions exhibited statistically significant variations across ‘plantation types’ (mixed-species and mono-species) and ‘sampling locations’ (Kerala and Coonoor). In the correlation and GLM analyses, it was evident that soil nutrients, metabolites, pH, and heavy metals played a crucial role in the difference in alpha diversity across the samples. Heavy metal concentrations were observed to be associated with the sampling locations (higher concentrations in the samples from Kerala). At the same time, the soil nutrients and metabolites (especially tepenoids and fatty alcohols) were higher in the mixed-species samples compared to the mono-species samples. Hence, ‘plantation types’ and ‘sampling locations’ may have a synergetic influence on the variations observed in the soil microbiome compositions across the samples. The major rhizosphere bacteria detected across the samples, contribute to plant health through their ability to control pathogens and promote plant growth. These variations may attribute to the higher diversity and heterogeneity of the mixed-species samples and to the variations in geographical parameters such as altitude across the sampling sites. The reduced bacterial diversity may be due to the higher heavy metal concentration and pollutants in the TM3 sample. The lack of sampling across the seasons is a limitation of the present study. Further studies across different seasons will provide much deeper insights into the dynamic variations in the microbiome and metabolome composition of these rhizosphere soils under various weather conditions.

## Data Availability

The datasets presented in this study can be found in online repositories. The names of the repository/repositories and accession number(s) can be found in the article/Supplementary material.

## Footnotes

1
https://www.tnurbantree.tn.gov.in/coonoor/

2
https://mausam.imd.gov.in/chennai/mcdata/extreme_coonoor.pdf

3
https://elevationmap.net/

4
https://www.weather-atlas.com/en

5
https://weather-and-climate.com/

6
http://classyfire.wishartlab.com/

7
https://bioinformatics.psb.ugent.be/webtools/Venn/

8
https://www.metaboanalyst.ca/

9
http://www.metagenassist.ca/

## References

[ref1] Adedeji A. A. Babalola O. O. (2020). Secondary metabolites as plant defensive strategy: a large role for small molecules in the near root region. Planta 252:61. doi: 10.1007/s00425-020-03468-1 32965531

[ref2] Aluko O. O. Li C. Wang Q. Liu H. (2021). Sucrose utilization for improved crop yields: a review article. Int. J. Mol. Sci. 22:4704. doi: 10.3390/ijms22094704, 33946791 PMC8124652

[ref3] Amoo A. E. Enagbonma B. J. Babalola O. O. (2019). High-throughput sequencing data of soil bacterial communities from Tweefontein indigenous and commercial forests, South Africa. Data Brief 28:104916. doi: 10.1016/j.dib.2019.104916, 31890783 PMC6926133

[ref4] Ancuceanu R. Hovaneț M. V. Miron A. Anghel A. I. Dinu M. (2023). Phytochemistry, biological, and pharmacological properties of *Abies alba* Mill. Plants (Basel). 12:2860. doi: 10.3390/plants12152860 37571016 PMC10421038

[ref5] Andrade P. H. M. Machado P. C. Paula A. F. Paganin A. C. L. Rezende G. S. Matheucci E. Jr. . (2023). 16S metabarcoding analysis reveals the influence of organic and conventional farming practices on bacterial communities from the rhizospheric of *Coffea arabica* L. Braz. J. Biol. 83:e274070. doi: 10.1590/1519-6984.274070, 37937628

[ref6] Araniti F. Graña E. Krasuska U. Bogatek R. Reigosa M. J. Abenavoli M. R. . (2016). Loss of gravitropism in Farnesene-treated Arabidopsis is due to microtubule malformations related to hormonal and ROS unbalance. PLoS One 11:e0160202. doi: 10.1371/journal.pone.0160202, 27490179 PMC4974009

[ref7] Arias D. M. Teasdale P. R. Doolette C. L. Lombi E. Farquhar S. Huang J. (2021). Development and evaluation of a new colorimetric DGT technique for the 2D visualisation of labile phosphate in soils. Chemosphere 269:128704. doi: 10.1016/j.chemosphere.2020.128704 33220985

[ref8] Arndt D. Xia J. Liu Y. Zhou Y. Guo A. C. Cruz J. A. . (2012). METAGENassist: a comprehensive web server for comparative metagenomics. Nucleic Acids Res. 40, W88–W95. doi: 10.1093/nar/gks497, 22645318 PMC3394294

[ref9] Augusto L. De Schrijver A. Vesterdal L. Smolander A. Prescott C. Ranger J. (2015). Influences of evergreen gymnosperm and deciduous angiosperm tree species on the functioning of temperate and boreal forests. Biol. Rev. 90, 444–466. doi: 10.1111/brv.12119, 24916992

[ref10] Babu K. N. Mandyam S. Jetty S. Dar A. A. Ayushi K. Narayanan A. . (2023). Carbon stocks of tree plantations in a Western Ghats landscape, India: influencing factors and management implications. Environ. Monit. Assess. 195:404. doi: 10.1007/s10661-023-10964-w, 36792838

[ref11] Bárcenas-Moreno G. Jiménez-Compán E. San Emeterio L. M. Jiménez-Morillo N. T. González-Pérez J. A. (2022). Soil pH and soluble organic matter shifts exerted by heating affect microbial response. Int. J. Environ. Res. Public Health 19:15751. doi: 10.3390/ijerph192315751, 36497826 PMC9735712

[ref12] Barreto I. C. de Almeida A. S. Sena Filho J. G. (2021). Taxonomic insights and its type cyclization correlation of volatile Sesquiterpenes in Vitex species and potential source insecticidal compounds: a review. Molecules 26:6405. doi: 10.3390/molecules26216405, 34770814 PMC8587464

[ref13] Beier A. Hahn V. Bornscheuer U. T. Schauer F. (2014). Metabolism of alkenes and ketones by Candida maltosa and related yeasts. AMB Express 4:75. doi: 10.1186/s13568-014-0075-2 25309846 PMC4192553

[ref14] BenIsrael M. Habtewold J. Z. Khosla K. Wanner P. Aravena R. Parker B. L. . (2021). Identification of degrader bacteria and fungi enriched in rhizosphere soil from a toluene phytoremediation site using DNA stable isotope probing. Int. J. Phytoremediation 23, 846–856. doi: 10.1080/15226514.2020.1860901, 33397125

[ref15] Bera I. Nidhin I. K. Hembrom M. E. Das K. Chattopadhyay I. (2023). Metagenomics offers insights into the rhizospheric bacterial diversity of mushrooms from a tropical forest and temperate forest of India. Ecol. Genet. Genom. 29:100203. doi: 10.1016/j.egg.2023.100203

[ref16] Berntsen H. F. Berg V. Thomsen C. Ropstad E. Zimmer K. E. (2017). The design of an environmentally relevant mixture of persistent organic pollutants for use in in vivo and in vitro studies. J. Toxicol. Environ. Health A 80, 1002–1016. doi: 10.1080/15287394.2017.1354439, 28854125

[ref17] Bhattacharyya A. Mavrodi O. Bhowmik N. Weller D. Thomashow L. Mavrodi D. (2023). Bacterial biofilms as an essential component of rhizosphere plant-microbe interactions. Methods Microbiol. 53, 3–48. doi: 10.1016/bs.mim.2023.05.006, 38415193 PMC10898258

[ref18] Bolivar-Torres H. H. Marín-Paredes R. Ramos-Madrigal C. Servín-Garcidueñas L. E. (2022). Metagenome-assembled genome of *Acidibrevibacterium fodinaquatile* FLA01 from fumarole sediments from the Los Azufres geothermal field. Microbiol Resour Announc. 11:e0082322. doi: 10.1128/mra.00823-22, 36190231 PMC9583776

[ref19] Bonan G. B. (2008). Forests and climate change: forcings, feedbacks, and the climate benefits of forests. Science 320, 1444–1449. doi: 10.1126/science.1155121, 18556546

[ref20] Bonaventure G. Salas J. J. Pollard M. R. Ohlrogge J. B. (2003). Disruption of the FATB gene in Arabidopsis demonstrates an essential role of saturated fatty acids in plant growth. Plant Cell 15, 1020–1033. doi: 10.1105/tpc.008946 12671095 PMC152346

[ref21] Bruto M. Prigent-Combaret C. Muller D. Moënne-Loccoz Y. (2014). Analysis of genes contributing to plant-beneficial functions in plant growth-promoting Rhizobacteria and related Proteobacteria. Sci. Rep. 4:6261. doi: 10.1038/srep06261, 25179219 PMC4151105

[ref22] Cepas V. Ballén V. Gabasa Y. Ramírez M. López Y. Soto S. M. (2020). Transposon insertion in the purL gene induces biofilm depletion in *Escherichia coli* ATCC 25922. Pathogens. 9:774. doi: 10.3390/pathogens9090774, 32971800 PMC7558270

[ref23] Chagas F. O. Pessotti R. C. Caraballo-Rodríguez A. M. Pupo M. T. (2018). Chemical signaling involved in plant-microbe interactions. Chem. Soc. Rev. 47, 1652–1704. doi: 10.1039/c7cs00343a 29218336

[ref24] Chai Y. N. Schachtman D. P. (2022). Root exudates impact plant performance under abiotic stress. Trends Plant Sci. 27, 80–91. doi: 10.1016/j.tplants.2021.08.003 34481715

[ref25] Chaimovitsh D. Abu-Abied M. Belausov E. Rubin B. Dudai N. Sadot E. (2010). Microtubules are an intracellular target of the plant terpene citral. Plant J. Cell Mol. Biol. 61, 399–408. doi: 10.1111/j.1365-313X.2009.04063.x, 19891702

[ref26] Chama M. A. Dziwornu G. A. Popli E. Mas-Claret E. Egyir B. Ayine-Tora D. M. . (2023). Antimicrobial and in silico studies of the triterpenoids of *Dichapetalum albidum*. Heliyon 9:e18299. doi: 10.1016/j.heliyon.2023.e18299, 37539285 PMC10395534

[ref27] Chaparro J. M. Badri D. V. Vivanco J. M. (2014). Rhizosphere microbiome assemblage is affected by plant development. ISME J. 8, 790–803. doi: 10.1038/ismej.2013.196, 24196324 PMC3960538

[ref28] Chen L. Liu Y. (2024). The function of root exudates in the root colonization by beneficial soil Rhizobacteria. Biology (Basel). 13:95. doi: 10.3390/biology13020095, 38392313 PMC10886372

[ref29] Chen F. Ro D. K. Petri J. Gershenzon J. Bohlmann J. Pichersky E. . (2004). Characterization of a root-specific Arabidopsis terpene synthase responsible for the formation of the volatile monoterpene 1,8-cineole. Plant Physiol. 135, 1956–1966. doi: 10.1104/pp.104.044388, 15299125 PMC520767

[ref30] Cheng S. S. Chang S. T. (2014). Bioactivity and characterization of exudates from *Cryptomeria japonica* bark. Wood Sci. Technol. 48, 831–840. doi: 10.1007/s00226-014-0644-1

[ref31] Chomel M. Guittonny-Larchevêque M. Fernandez C. Gallet C. DesRochers A. Paré D. . (2016). Plant secondary metabolites: a key driver of litter decomposition and soil nutrient cycling. J. Ecol. 104, 1527–1541. doi: 10.1111/1365-2745.12644

[ref32] Chou M. Y. Andersen T. B. Mechan Llontop M. E. Beculheimer N. Sow A. Moreno N. . (2023). Terpenes modulate bacterial and fungal growth and sorghum rhizobiome communities. Microbiol. Spectrum 11:e0133223. doi: 10.1128/spectrum.01332-23, 37772854 PMC10580827

[ref33] de Sousa L. P. Guerreiro-Filho O. Mondego J. M. C. (2022). The rhizosphere microbiomes of five species of coffee trees. Microbiol. Spectrum 10:e0044422. doi: 10.1128/spectrum.00444-22 PMC9045209 35289671

[ref34] Dedysh S. N. Beletsky A. V. Kulichevskaya I. S. Mardanov A. V. Ravin N. V. (2021). Complete genome sequence of *Paludibaculum fermentans* P105T, a Facultatively anaerobic Acidobacterium capable of dissimilatory Fe(III) reduction. Microbiol Resour Announc. 10, e01313–e01320. doi: 10.1128/MRA.01313-20, 33414319 PMC8407742

[ref35] Dickschat J. S. (2016). Bacterial terpene cyclases. Nat. Prod. Rep. 33, 87–110. doi: 10.1039/c5np00102a, 26563452

[ref36] Dukunde A. Schneider D. Schmidt M. Veldkamp E. Daniel R. (2019). Tree species shape soil bacterial community structure and function in temperate deciduous forests. Front. Microbiol. 10:1519. doi: 10.3389/fmicb.2019.01519, 31338079 PMC6629791

[ref37] Eaton R. W. Sandusky P. (2009). Biotransformations of 2-methylisoborneol by camphor-degrading bacteria. Appl. Environ. Microbiol. 75, 583–588. doi: 10.1128/AEM.02126-08 19060161 PMC2632152

[ref38] Eaton R. W. Sandusky P. (2010). Biotransformations of (+/−)-geosmin by terpene-degrading bacteria. Biodegradation 21, 71–79. doi: 10.1007/s10532-009-9282-y, 19578827

[ref39] Elaiyaraja A. Mayilsamy M. Vimalkumar K. Nikhil N. P. Noorani P. M. Bommuraj V. . (2022). Aquatic and human health risk assessment of Humanogenic emerging contaminants (HECs), phthalate esters from the Indian Rivers. Chemosphere 306:135624. doi: 10.1016/j.chemosphere.2022.135624, 35810861

[ref40] Falagán C. Johnson D. B. (2014). *Acidibacter ferrireducens* gen. Nov., sp. nov.: an acidophilic ferric iron-reducing gamma proteobacterium. Extremophiles 18, 1067–1073. doi: 10.1007/s00792-014-0684-3, 25116055

[ref41] Feng H. Fu R. Hou X. Lv Y. Zhang N. Liu Y. . (2021). Chemotaxis of Beneficial Rhizobacteria to Root Exudates: The First Step towards Root-Microbe Rhizosphere Interactions. Int J Mol Sci. 22:6655. doi: 10.3390/ijms22136655 34206311 PMC8269324

[ref42] Fonseca E. D. S. Peixoto R. S. Rosado A. S. Balieiro F. C. Tiedje J. M. Rachid C. T. C. D. C. (2018). The microbiome of eucalyptus roots under different management conditions and its potential for biological nitrogen fixation. Microb. Ecol. 75, 183–191. doi: 10.1007/s00248-017-1122-8 28634640

[ref43] Fourneau E. Pannier M. Riah W. Personeni E. Morvan-Bertrand A. Bodilis J. . (2024). A "love match" score to compare root exudate attraction and feeding of the plant growth-promoting rhizobacteria *Bacillus subtilis*, *Pseudomonas fluorescens*, and *Azospirillum brasilense*. Front. Microbiol. 15:1473099. doi: 10.3389/fmicb.2024.1473099, 39376706 PMC11456545

[ref44] Fozo E. M. Quivey R. G. Jr. (2004). Shifts in the membrane fatty acid profile of *Streptococcus mutans* enhance survival in acidic environments. Appl. Environ. Microbiol. 70, 929–936. doi: 10.1128/AEM.70.2.929-936.2004, 14766573 PMC348902

[ref45] Francoeur C. B. Khadempour L. Moreira-Soto R. D. Gotting K. Book A. J. Pinto-Tomás A. A. . (2020). Bacteria contribute to plant secondary compound degradation in a generalist herbivore system. MBio 11, e02146–e02120. doi: 10.1128/mBio.02146-20, 32934088 PMC7492740

[ref46] Frankowski M. Zioła-Frankowska A. Kurzyca I. Novotný K. Vaculovič T. Kanický V. . (2011). Determination of aluminium in groundwater samples by GF-AAS, ICP-AES, ICP-MS and modelling of inorganic aluminium complexes. Environ. Monit. Assess. 182, 71–84. doi: 10.1007/s10661-010-1859-8 21274747

[ref47] Fry B. N. Feng S. Chen Y. Y. Newell D. G. Coloe P. J. Korolik V. (2000). The *galE* gene of *Campylobacter jejuni* is involved in lipopolysaccharide synthesis and virulence. Infect. Immun. 68, 2594–2601. doi: 10.1128/IAI.68.5.2594-2601.2000, 10768949 PMC97464

[ref48] Gambuś F. Wieczorek J. (2012). Pollution of fertilizers with heavy metals. Ecol. Chem. Eng. A 19, 353–360.

[ref49] Ghaly A. E. Mahmoud N. S. (2007). Effects of tetrazolium chloride concentration, O2, and cell age on dehydrogenase activity of *Aspergillus niger*. Appl. Biochem. Biotechnol. 136, 207–222. doi: 10.1007/BF02686018, 17496341

[ref50] Ghare U. Narvekar S. Lodha T. Mallebhari R. Dastager S. Barvkar V. T. . (2023). Bacterial communities and diversity of Western Ghats soil: a study of a biodiversity hotspot. Curr. Microbiol. 80:108. doi: 10.1007/s00284-023-03207-1 36807001

[ref51] Guo B. Wen A. Yu H. Guo Y. Cheng Y. Xie Y. . (2023). Interaction between six waxy components in summer black grapes (*Vitis vinifera*) and Mancozeb and its effect on the residue of Mancozeb. Int. J. Mol. Sci. 24:7705. doi: 10.3390/ijms24097705, 37175414 PMC10178566

[ref52] Heider S. A. Peters-Wendisch P. Beekwilder J. Wendisch V. F. (2014). IdsA is the major geranylgeranyl pyrophosphate synthase involved in carotenogenesis in *Corynebacterium glutamicum*. FEBS J. 281, 4906–4920. doi: 10.1111/febs.13033 25181035

[ref53] Himaman W. Thamchaipenet A. Pathom-Aree W. Duangmal K. (2016). Actinomycetes from Eucalyptus and their biological activities for controlling Eucalyptus leaf and shoot blight. Microbiol. Res. 188-189, 42–52. doi: 10.1016/j.micres.2016.04.011, 27296961

[ref54] Huang Y. X. Wu Z. J. Zong Y. Y. Li W. Q. Chen F. S. Wang G. G. . (2022). Mixing with coniferous tree species alleviates rhizosphere soil phosphorus limitation of broad-leaved trees in subtropical plantations. Soil Biol. Biochem. 175:108853. doi: 10.1016/j.soilbio.2022.108853

[ref55] Huo C. Zhang J. Yang X. Li X. Su Y. Chen Z. (2024). Dry season irrigation promotes nutrient cycling by reorganizing Eucalyptus rhizosphere microbiome. Sci. Total Environ. 954:176307. doi: 10.1016/j.scitotenv.2024.176307, 39284445

[ref56] Imdad S. So B. Jang J. Park J. Lee S. J. Kim J. H. . (2024). Temporal variations in the gut microbial diversity in response to high-fat diet and exercise. Sci. Rep. 14:3282. doi: 10.1038/s41598-024-52852-4 38332014 PMC10853223

[ref57] Iqbal A. Maqsood Ur Rehman M. Sajjad W. Degen A. A. Rafiq M. Jiahuan N. . (2024). Patterns of bacterial communities in the rhizosphere and rhizoplane of alpine wet meadows. Environ. Res. 241:117672. doi: 10.1016/j.envres.2023.117672, 37980986

[ref58] Jacoby R. P. Koprivova A. Kopriva S. (2021). Pinpointing secondary metabolites that shape the composition and function of the plant microbiome. J. Exp. Bot. 72, 57–69. doi: 10.1093/jxb/eraa424, 32995888 PMC7816845

[ref59] Januszek K. Blonska E. Dluga J. Socha J. (2015). Dehydrogenase activity of forest soils depends on the assay used. Int. Agrophys. 29, 47–59. doi: 10.1515/intag-2015-0009

[ref60] Jeevith S. Manjunath J. (2023). Urban trees of the Nilgiris district, Tamil Nadu, India. Biodiversity Res. Conservat. 69, 1–12. doi: 10.14746/biorc.2023.69.2

[ref61] Jha Y. Subramanian R. B. (2014). Characterization of root-associated bacteria from paddy and its growth-promotion efficacy. 3. Biotech 4, 325–330. doi: 10.1007/s13205-013-0158-9, 28324437 PMC4026455

[ref62] Ji K. Wei Y. Lan G. (2024). Geographic location affects the bacterial community composition and diversity more than species identity for tropical tree species. Plants (Basel). 13:1565. doi: 10.3390/plants13111565, 38891373 PMC11175100

[ref63] Jia F. Chen Y. Xu Z. Gao X. Mei N. Qi X. . (2025). FeO might be more suitable than Fe2+ for the construction of anammox-dominated Fe-N coupling system: based on 15N isotope tracing. Water Res. 274:123097. doi: 10.1016/j.watres.2025.123097, 39842215

[ref64] Jiao J. Y. Liu L. Zhou E. M. Wei D. Q. Ming H. Xian W. D. . (2015). *Actinomadura amylolytica* sp. nov. and *Actinomadura cellulosilytica* sp. nov., isolated from geothermally heated soil. Antonie Van Leeuwenhoek 108, 75–83. doi: 10.1007/s10482-015-0465-8, 25944084

[ref65] Jin R. Bu D. Liu G. Zheng M. Lammel G. Fu J. . (2020). New classes of organic pollutants in the remote continental environment - chlorinated and brominated polycyclic aromatic hydrocarbons on the Tibetan plateau. Environ. Int. 137:105574. doi: 10.1016/j.envint.2020.105574, 32078871

[ref66] Jin J. Kim M. J. Dhandapani S. Tjhang J. G. Yin J. L. Wong L. . (2015). The floral transcriptome of ylang ylang (*Cananga odorata* var. fruticosa) uncovers biosynthetic pathways for volatile organic compounds and a multifunctional and novel sesquiterpene synthase. J. Exp. Bot. 66, 3959–3975. doi: 10.1093/jxb/erv196, 25956881 PMC4473991

[ref67] Kalam S. Basu A. Ahmad I. Sayyed R. Z. El-Enshasy H. A. Dailin D. J. . (2020). Recent understanding of soil acidobacteria and their ecological significance: a critical review. Front. Microbiol. 11:580024. doi: 10.3389/fmicb.2020.580024, 33193209 PMC7661733

[ref68] Kandziora-Ciupa M. Dabioch M. Nadgórska-Socha A. (2022). Evaluating the accumulation of antioxidant and macro- and trace elements in *Vaccinium myrtillus* L. Biol. Trace Element Res. 200, 4175–4185. doi: 10.1007/s12011-021-02989-4 PMC9374609 34714487

[ref69] Kim S. J. Ahn J. H. Lee T. H. Weon H. Y. Hong S. B. Seok S. J. . (2013). *Reyranella soli* sp. nov., isolated from forest soil, and emended description of the genus *Reyranella Pagnier* et al. 2011. Int. J. Syst. Evol. Microbiol. 63, 3164–3167. doi: 10.1099/ijs.0.045922-0, 23435248

[ref70] Kim H. J. Lee H. J. Lim B. Kim E. Kim H. Y. Suh M. . (2018). *Lactobacillus terrae* sp. nov., a novel species isolated from soil samples in the Republic of Korea. Int. J. Syst. Evol. Microbiol. 68, 2906–2911. doi: 10.1099/ijsem.0.002918 30010525

[ref71] Korenblum E. Dong Y. Szymanski J. Panda S. Jozwiak A. Massalha H. . (2020). Rhizosphere microbiome mediates systemic root metabolite exudation by root-to-root signaling. Proc. Natl. Acad. Sci. USA 117, 3874–3883. doi: 10.1073/pnas.1912130117, 32015118 PMC7035606

[ref72] Korenblum E. Massalha H. Aharoni A. (2022). Plant-microbe interactions in the rhizosphere via a circular metabolic economy. Plant Cell 34, 3168–3182. doi: 10.1093/plcell/koac163, 35678568 PMC9421461

[ref73] Kovaleva O. L. Merkel A. Y. Novikov A. A. Baslerov R. V. Toshchakov S. V. Bonch-Osmolovskaya E. A. (2015). *Tepidisphaera mucosa* gen. Nov., sp. nov., a moderately thermophilic member of the class Phycisphaerae in the phylum Planctomycetes, and proposal of a new family, Tepidisphaeraceae fam. Nov., and a new order, Tepidisphaerales Ord. Nov. Int. J. Syst. Evol. Microbiol. 65, 549–555. doi: 10.1099/ijs.0.070151-0, 25404483

[ref74] Kulichevskaya I. S. Ivanova A. A. Suzina N. E. Rijpstra W. I. C. Sinninghe Damsté J. S. Dedysh S. N. (2016). *Paludisphaera borealis* gen. Nov., sp. nov., a hydrolytic planctomycete from northern wetlands, and proposal of Isosphaeraceae fam. Nov. Int. J. Syst. Evol. Microbiol. 66, 837–844. doi: 10.1099/ijsem.0.000799 26611145

[ref75] Lacerda-Júnior G. V. Noronha M. F. Cabral L. Delforno T. P. de Sousa S. T. P. Fernandes-Júnior P. I. . (2019). Land use and seasonal effects on the soil microbiome of a Brazilian dry Forest. Front. Microbiol. 10:648. doi: 10.3389/fmicb.2019.00648, 31024471 PMC6461016

[ref76] Lan G. Wei Y. Li Y. Wu Z. (2023). Diversity and assembly of root-associated microbiomes of rubber trees. Front. Plant Sci. 14:1136418. doi: 10.3389/fpls.2023.1136418, 37063173 PMC10102524

[ref77] Leferink N. G. H. Jervis A. J. Zebec Z. Toogood H. S. Hay S. Takano E. . (2016). A 'plug and play' platform for the production of diverse monoterpene hydrocarbon scaffolds in *Escherichia coli*. ChemistrySelect 1, 1893–1896. doi: 10.1002/slct.201600563, 29756025 PMC5947754

[ref78] Lei J. Wu H. Li X. Guo W. Duan A. Zhang J. (2023). Response of rhizosphere bacterial communities to near-natural forest management and tree species within Chinese fir plantations. Microbiol. Spectrum 11:e0232822. doi: 10.1128/spectrum.02328-22, 36688690 PMC9927156

[ref79] Lemanowicz J. Haddad S. A. Bartkowiak A. Lamparski R. Wojewódzki P. (2020). The role of an urban park's tree stand in shaping the enzymatic activity, glomalin content and physicochemical properties of soil. Sci. Total Environ. 741:140446. doi: 10.1016/j.scitotenv.2020.140446, 32887013

[ref80] Li H. Kang Z. Hua J. Feng Y. Luo S. (2022). Root exudate sesquiterpenoids from the invasive weed *Ambrosia trifida* regulate rhizospheric Proteobacteria. Sci. Total Environ. 834:155263. doi: 10.1016/j.scitotenv.2022.155263, 35439515

[ref81] Li X. Li Z. (2023). What determines symbiotic nitrogen fixation efficiency in rhizobium: recent insights into *Rhizobium leguminosarum*. Arch. Microbiol. 205:300. doi: 10.1007/s00203-023-03640-7, 37542687

[ref82] Li X. Li B. Liu Y. Xu J. (2023). Rhizospheric *Lactobacillus* spp. contribute to the high cd-accumulating characteristics of *Phytolacca* spp. in acidic cd-contaminated soil. Environ. Res. 238:117270. doi: 10.1016/j.envres.2023.117270 37776944

[ref83] Li Q. Wan F. Zhao M. (2022). Distinct soil microbial communities under *Ageratina adenophora* invasions. Plant Biol. (Stuttg.) 24, 430–439. doi: 10.1111/plb.13387, 35050505

[ref84] Li W. Xie L. Xu Y. Yang M. (2024). Effect of mixed planting on soil nutrient availability and microbial diversity in the rhizosphere of *Parashorea chinensis* plantations. Front Microbiol. 15:1464271. doi: 10.3389/fmicb.2024.1464271 39473841 PMC11520325

[ref85] Lin L. Ge H. M. Yan T. Qin Y. H. Tan R. X. (2012). Thaxtomin A-deficient endophytic *Streptomyces* sp. enhances plant disease resistance to pathogenic Streptomyces scabies. Planta 236, 1849–1861. doi: 10.1007/s00425-012-1741-8, 22922880

[ref86] Lin Q. Li M. Wang Y. Xu Z. Li L. (2022). Root exudates and chemotactic strains mediate bacterial community assembly in the rhizosphere soil of *Casuarina equisetifolia* L. Front. Plant Sci. 13:988442. doi: 10.3389/fpls.2022.988442, 36212345 PMC9534574

[ref87] Liu Y. Wang L. H. Hao C. B. Li L. Li S. Y. Feng C. P. (2014). Microbial diversity and ammonia-oxidizing microorganism of a soil sample near an acid mine drainage lake. Huan Jing Ke Xue 35, 2305–2313. doi: 10.13227/j.hjkx.2014.06.037, 25158511

[ref88] Liu Y. Xu Z. Chen L. Xun W. Shu X. Chen Y. . (2024). Root colonization by beneficial rhizobacteria. FEMS Microbiol. Rev. 48:fuad066. doi: 10.1093/femsre/fuad066, 38093453 PMC10786197

[ref89] Liu H. Zhu J. Hu Q. Rao X. (2016). *Morganella morganii*, a non-negligent opportunistic pathogen. Int. J. Infect. Dis. 50, 10–17. doi: 10.1016/j.ijid.2016.07.006, 27421818

[ref90] Lladó S. López-Mondéjar R. Baldrian P. (2017). Forest soil bacteria: diversity, involvement in ecosystem processes, and response to global change. Microbiol. Mol. Biol. Rev. 81, e00063–e00016. doi: 10.1128/MMBR.00063-16, 28404790 PMC5485800

[ref91] Lu Y. Kronzucker H. J. Shi W. (2021). Stigmasterol root exudation arising from Pseudomonas inoculation of the duckweed rhizosphere enhances nitrogen removal from polluted waters. Environ Pollut. 15:117587. doi: 10.1016/j.envpol.2021.117587 34182390

[ref92] Lu Y. Zhou G. Ewald J. Pang Z. Shiri T. Xia J. (2023). Microbiome analyst 2.0: comprehensive statistical, functional and integrative analysis of microbiome data. Nucleic Acids Res. 51, W310–W318. doi: 10.1093/nar/gkad407, 37166960 PMC10320150

[ref93] Mączka W. Duda-Madej A. Górny A. Grabarczyk M. Wińska K. (2021). Can eucalyptol replace antibiotics? Molecules 26:4933. doi: 10.3390/molecules26164933, 34443521 PMC8398027

[ref94] Mannaa M. Han G. Jeon H. W. Kim J. Kim N. Park A. R. . (2020). Influence of resistance-inducing chemical elicitors against pine wilt disease on the rhizosphere microbiome. Microorganisms. 8:884. doi: 10.3390/microorganisms8060884, 32545246 PMC7356868

[ref95] Mao Y. H. Li F. Ma J. C. Hu Z. Wang H. H. (2016). *Sinorhizobium meliloti* Functionally replaces 3-Oxoacyl-acyl carrier protein reductase (FabG) by overexpressing NodG during fatty acid synthesis. Mol. Plant-Microbe Interact. 29, 458–467. doi: 10.1094/MPMI-07-15-0148-R, 26975437

[ref96] Marmitt M. Cauduro G. P. Sbruzzi R. C. Valiati V. H. (2024). Evaluation of differentially expressed candidate genes in benzo[a]pyrene degradation by *Burkholderia vietnamiensis* G4. Mol. Biotechnol. 67, 3673–3684. doi: 10.1007/s12033-024-01284-6, 39298104

[ref97] Martineau C. Mauffrey F. Villemur R. (2015). Comparative analysis of denitrifying activities of *Hyphomicrobium nitrativorans*, *Hyphomicrobium denitrificans*, and *Hyphomicrobium zavarzinii*. Appl. Environ. Microbiol. 81, 5003–5014. doi: 10.1128/AEM.00848-15, 25979892 PMC4495217

[ref98] Massalha H. Korenblum E. Tholl D. Aharoni A. (2017). Small molecules below-ground: the role of specialized metabolites in the rhizosphere. Plant J. 90, 788–807. doi: 10.1111/tpj.13543, 28333395

[ref99] Mathur V. Ulanova D. (2023). Microbial metabolites beneficial to plant hosts across ecosystems. Microb. Ecol. 86, 25–48. doi: 10.1007/s00248-022-02073-x, 35867138

[ref100] Meena A. Rao K. S. (2021). Assessment of soil microbial and enzyme activity in the rhizosphere zone under different land use/cover of a semiarid region, India. Ecol. Process. 10:16. doi: 10.1186/s13717-021-00288-3

[ref101] Meena M. Yadav G. Sonigra P. Nagda A. Mehta T. Swapnil P. . (2022). Role of elicitors to initiate the induction of systemic resistance in plants to biotic stress. Plant. Stress 5:100103. doi: 10.1016/j.stress.2022.100103

[ref102] Megyes M. Borsodi A. K. Árendás T. Márialigeti K. (2021). Variations in the diversity of soil bacterial and archaeal communities in response to different long-term fertilization regimes in maize fields. Appl. Soil Ecol. 168:104120. doi: 10.1016/j.apsoil.2021.104120

[ref103] Mehmood M. A. Fu Y. Zhao H. Cheng J. Xie J. Jiang D. (2022). Enrichment of bacteria involved in the nitrogen cycle and plant growth promotion in soil by sclerotia of rice sheath blight fungus. Stress Biol. 2:32. doi: 10.1007/s44154-022-00049-y, 37676387 PMC10441917

[ref104] Meira-Neto J. A. A. Tolentino G. S. Silva M. C. N. A. D. Neri A. V. Gastauer M. Magnago L. F. S. . (2017). Functional antagonism between nitrogen-fixing leguminous trees and calcicole-drought-tolerant trees in the Cerrado. Acta Botanica Brasilica 31, 11–18. doi: 10.1590/0102-33062016abb0380

[ref105] Messier C. Bauhus J. Sousa-Silva R. Auge H. Baeten L. Barsoum N. . (2022). For the sake of resilience and multifunctionality, let's diversify planted forests! Conserv. Lett. 15:e12829. doi: 10.1111/conl.12829

[ref106] Mhlongo M. I. Piater L. A. Dubery I. A. (2022). Profiling of volatile organic compounds from four plant growth-promoting Rhizobacteria by SPME-GC-MS: a metabolomics study. Meta 12:763. doi: 10.3390/metabo12080763, 36005635 PMC9414699

[ref107] Mir Y. H. Ganie M. A. Shah T. I. Bangroo S. A. Mir S. A. Shah A. M. . (2023). Soil microbial and enzyme activities in different land use systems of the northwestern Himalayas. PeerJ. 11:e15993. doi: 10.7717/peerj.15993, 37780386 PMC10540776

[ref108] Muhlbachova G. Sagova-Mareckova M. Omelka M. Szakova J. Tlustos P. (2015). The influence of soil organic carbon on interactions between microbial parameters and metal concentrations at a long-term contaminated site. Sci. Total Environ. 502, 218–223. doi: 10.1016/j.scitotenv.2014.08.079 25260167

[ref109] Mujakić I. Piwosz K. Koblížek M. (2022). Phylum *Gemmatimonadota* and its role in the environment. Microorganisms. 10:151. doi: 10.3390/microorganisms10010151, 35056600 PMC8779627

[ref110] Müller C. Junker R. R. (2022). Chemical phenotype as important and dynamic niche dimension of plants. New Phytol. 234, 1168–1174. doi: 10.1111/nph.18075 35297052

[ref111] Musongora M. Karanja N. Kimenju W. Kamau S. (2023). Spatio-temporal change of selected soil physico-chemical properties in grevillea-banana agroforestry systems. Heliyon. 9:e16121. doi: 10.1016/j.heliyon.2023.e16121, 37234607 PMC10208817

[ref112] Nasir Shah S. Khan I. Tul Muntaha S. Hayat A. Ur Rehman M. Ali Shah T. . (2023). Bioactive, antioxidant and antimicrobial properties of chemically fingerprinted essential oils extracted from *Eucalyptus globulus*: *in-vitro* and *in-silico* investigations. Front. Chem. 11:1287317. doi: 10.3389/fchem.2023.1287317, 38188929 PMC10768562

[ref113] Nath S. Sarkar M. Maddheshiya A. De D. Paul S. Dey S. . (2023). Upper respiratory tract microbiome profiles in SARS-CoV-2 Delta and omicron infected patients exhibit variant specific patterns and robust prediction of disease groups. Microbiol. Spectrum 11:e0236823. doi: 10.1128/spectrum.02368-23, 37905804 PMC10715160

[ref114] Navnage N. P. Patle P. N. Ramteke P. R. (2018). Dehydrogenase activity (DHA): measure of total microbial activity and as indicator of soil quality. Int. J. Chem. Stud 20, 456–458. doi: 10.1371/journal.pone.0328055, 40880451 PMC12396641

[ref115] Neupane S. Goodwin L. A. Högberg N. Kyrpides N. C. Alström S. Bruce D. . (2013). Non-contiguous finished genome sequence of plant-growth promoting *Serratia proteamaculans* S4. Stand Genomic Sci. 8, 441–449. doi: 10.4056/sigs.4027757 24501629 PMC3910699

[ref116] Nuccio E. E. Starr E. Karaoz U. Brodie E. L. Zhou J. Tringe S. G. . (2020). Niche differentiation is spatially and temporally regulated in the rhizosphere. ISME J. 14, 999–1014. doi: 10.1038/s41396-019-0582-x 31953507 PMC7082339

[ref117] Nwachukwu B. C. Ayangbenro A. S. Babalola O. O. (2023). Structural diversity of bacterial communities in two divergent sunflower rhizosphere soils. Ann. Microbiol. 73:9. doi: 10.1186/s13213-023-01713-y

[ref118] Oppenheimer-Shaanan Y. Jakoby G. Starr M. L. Karliner R. Eilon G. Itkin M. . (2022). A dynamic rhizosphere interplay between tree roots and soil bacteria under drought stress. eLife 11:e79679. doi: 10.7554/eLife.79679, 35858113 PMC9385208

[ref119] Orasch T. Dietl A. M. Shadkchan Y. Binder U. Bauer I. Lass-Flörl C. . (2019). The leucine biosynthetic pathway is crucial for adaptation to iron starvation and virulence in *Aspergillus fumigatus*. Virulence 10, 925–934. doi: 10.1080/21505594.2019.1682760, 31694453 PMC6844326

[ref120] Ormeño-Orrillo E. Rosenblueth M. Luyten E. Vanderleyden J. Martínez-Romero E. (2008). Mutations in lipopolysaccharide biosynthetic genes impair maize rhizosphere and root colonization of *Rhizobium tropici* CIAT899. Environ. Microbiol. 10, 1271–1284. doi: 10.1111/j.1462-2920.2007.01541.x, 18312393

[ref121] Pang Z. Lu Y. Zhou G. Hui F. Xu L. Viau C. . (2024). MetaboAnalyst 6.0: towards a unified platform for metabolomics data processing, analysis and interpretation. Nucleic Acids Res. 52, W398–W406. doi: 10.1093/nar/gkae253, 38587201 PMC11223798

[ref122] Pascon R. C. Bergamo R. F. Spinelli R. X. de Souza E. D. Assis D. M. Juliano L. . (2011). Amylolytic microorganism from sãopaulo zoo composting: isolation, identification, and amylase production. Enzyme Res. 2011:679624. doi: 10.4061/2011/679624 21845217 PMC3154541

[ref123] Paulraj S. Bhat R. Rajesh M. K. Ramesh S. V. Priya U. K. Pandian R. T. P. . (2021). Data of 16S rRNA gene amplicon-based metagenomic signatures of arecanut rhizosphere soils in yellow leaf disease (YLD) endemic region of India. Data Brief 38:107443. doi: 10.1016/j.dib.2021.107443, 34746339 PMC8551408

[ref124] Pausch J. Kuzyakov Y. (2018). Carbon input by roots into the soil: quantification of rhizodeposition from root to ecosystem scale. Glob Change Biol. 24, 1–12. doi: 10.1111/gcb.13850, 28752603

[ref125] Qessaoui R. Bouharroud R. Furze J. N. El Aalaoui M. Akroud H. Amarraque A. . (2019). Applications of new Rhizobacteria *Pseudomonas* isolates in agroecology via fundamental processes complementing plant growth. Sci. Rep. 9:12832. doi: 10.1038/s41598-019-49216-8 31492898 PMC6731270

[ref126] Qiao H. Zhu H. Li H. Chen H. Li S. Chen C. . (2023). Isolation and characterization of gut bacteria associated with the degradation of host-specific terpenoids in *Pagiophloeus tsushimanus* (Coleoptera: Curculionidae) larvae. J. Insect Sci. 23:21. doi: 10.1093/jisesa/iead055, 37074003 PMC10114288

[ref127] Qu Z. Liu B. Ma Y. Sun H. (2020). Differences in bacterial community structure and potential functions among Eucalyptus plantations with different ages and species of trees. Appl. Soil Ecol. 149:103515. doi: 10.1016/j.apsoil.2020.103515

[ref128] Qu Z. L. Liu B. Zhang Y. M. Huang L. Ming A. G. Sun H. (2022). Impacts of near-natural management in eucalyptus plantations on soil bacterial community assembly and function related to nitrogen cycling. Funct. Ecol. 36, 1912–1923. doi: 10.1111/1365-2435.14106

[ref129] Radice M. Durofil A. Buzzi R. Baldini E. Martínez A. P. Scalvenzi L. . (2022). Alpha-phellandrene and alpha-phellandrene-rich essential oils: a systematic review of biological activities. Pharm. Food Appl. Life (Basel). 12:1602. doi: 10.3390/life12101602 PMC9605662 36295037

[ref130] Rahman M. M. Keya S. S. Sahu A. Gupta A. Dhingra A. Tran L. P. . (2024). Acetic acid: a cheap but chief metabolic regulator for abiotic stress tolerance in plants. Stress Biol. 4:34. doi: 10.1007/s44154-024-00167-9, 39073476 PMC11286891

[ref131] Ramos C. Magistro D. Walton G. E. Whitham A. Camp N. Poveda C. . (2025). Assessing the gut microbiota composition in older adults: connections to physical activity and healthy ageing. Geroscience. 47, 6039–6063. doi: 10.1007/s11357-025-01605-w, 40095191 PMC12397483

[ref132] Ramya D. Thatheyus A. J. Juliana S. J. B. Kiruba N. J. M. Selvam A. D. G. (2022). Physical characterization and kinetic studies of Zn (II) biosorption by *Morganella morganii* ACZ05. Water Sci. Technol. 85, 970–986. doi: 10.2166/wst.2022.031, 35228348

[ref133] Rangasamy E. Muniyandi M. (2025). Assessment of heavy metal pollution and human health risk in the soil of selected tea plantations from southern Western Ghats, Tamil Nadu, India. Toxicol. Rep. 15:102081. doi: 10.1016/j.toxrep.2025.102081, 40810008 PMC12343469

[ref134] Rathore P. Joy S. S. Yadav R. Ramakrishna W. (2021). Co-occurrence and patterns of phosphate solubilizing, salt and metal tolerant and antibiotic-resistant bacteria in diverse soils. 3 Biotech 11:356. doi: 10.1007/s13205-021-02904-7, 34249597 PMC8225767

[ref135] Rawat S. R. Männistö M. K. Bromberg Y. Häggblom M. M. (2012). Comparative genomic and physiological analysis provides insights into the role of Acidobacteria in organic carbon utilization in Arctic tundra soils. FEMS Microbiol. Ecol. 82, 341–355. doi: 10.1111/j.1574-6941.2012.01381.x 22486608

[ref136] Rivas-Marin E. Stettner S. Gottshall E. Y. Santana-Molina C. Helling M. Basile F. . (2019). Essentiality of sterol synthesis genes in the planctomycete bacterium *Gemmata obscuriglobus*. Nat. Commun. 10:2916. doi: 10.1038/s41467-019-10983-7 31266954 PMC6606645

[ref137] Robinson B. H. Yalamanchali R. Reiser R. Dickinson N. M. (2018). Lithium as an emerging environmental contaminant: mobility in the soil-plant system. Chemosphere 197, 1–6. doi: 10.1016/j.chemosphere.2018.01.012 29324285

[ref138] Rohini-Kumar M. Osborne J. W. Saravanan V. S. (2013). Comparison of soil bacterial communities of *Pinus patula* of Nilgiris, western ghats with other biogeographically distant pine forest clone libraries. Microb. Ecol. 66, 132–144. doi: 10.1007/s00248-012-0167-y 23274880

[ref139] Rolfe S. A. Griffiths J. Ton J. (2019). Crying out for help with root exudates: adaptive mechanisms by which stressed plants assemble health-promoting soil microbiomes. Curr. Opin. Microbiol. 49, 73–82. doi: 10.1016/j.mib.2019.10.003 31731229

[ref140] Rolli E. Vergani L. Ghitti E. Patania G. Mapelli F. Borin S. (2021). 'Cry-for-help' in contaminated soil: a dialogue among plants and soil microbiome to survive in hostile conditions. Environ. Microbiol. 23, 5690–5703. doi: 10.1111/1462-2920.15647 34139059 PMC8596516

[ref141] Rosenzweig N. Bradeen J. M. Tu Z. J. McKay S. J. Kinkel L. L. (2013). Rhizosphere bacterial communities associated with long-lived perennial prairie plants vary in diversity, composition, and structure. Can. J. Microbiol. 59, 494–502. doi: 10.1139/cjm-2012-0661 23826959

[ref142] Sánchez-Herrera L. M. Ramos-Valdivia A. C. de la Torre M. Salgado L. M. Ponce-Noyola T. (2007). Differential expression of cellulases and xylanases by *Cellulomonas flavigena* grown on different carbon sources. Appl. Microbiol. Biotechnol. 77, 589–595. doi: 10.1007/s00253-007-1190-7 17899068

[ref143] Satish K. V. Saranya K. R. Reddy C. S. Krishna P. H. Jha C. S. Rao P. V. (2014). Geospatial assessment and monitoring of historical forest cover changes (1920-2012) in Nilgiri biosphere reserve, Western Ghats, India. Environ. Monit. Assess. 186, 8125–8140. doi: 10.1007/s10661-014-3991-3, 25117494

[ref144] Selvaraj K. K. Sundaramoorthy G. Ravichandran P. K. Girijan G. K. Sampath S. Ramaswamy B. R. (2015). Phthalate esters in water and sediments of the Kaveri River, India: environmental levels and ecotoxicological evaluations. Environ. Geochem. Health 37, 83–96. doi: 10.1007/s10653-014-9632-5, 25056812

[ref145] Seo Y. Cho K. S. (2021). Effects of plant and soil amendment on remediation performance and methane mitigation in petroleum contaminated soil. J. Microbiol. Biotechnol. 31, 104–114. doi: 10.4014/jmb.2006.06023, 33144544 PMC9705697

[ref146] Shanbhag A. P. (2019). FabG: from a core to circumstantial catalyst. Biotechnol. Lett. 41, 675–688. doi: 10.1007/s10529-019-02691-5, 31037463

[ref147] Sharma P. Nadda A. K. Kumar S. (2023). Microbial community profiling in bio-stimulated municipal solid waste for effective removal of organic pollutants containing endocrine disrupting chemicals. Microbiol. Res. 267:127273. doi: 10.1016/j.micres.2022.127273, 36481500

[ref148] Sharma P. Singh N. Singh S. Khare S. K. Nain P. K. S. Nain L. (2021). Potent γ-amino butyric acid producing psychobiotic *Lactococcus lactis* LP-68 from non-rhizospheric soil of *Syzygium cumini* (black plum). Arch. Microbiol. 204:82. doi: 10.1007/s00203-021-02629-4, 34958412

[ref149] Sharma M. Sudheer S. Usmani Z. Rani R. Gupta P. (2020). Deciphering the omics of plant-microbe interaction: perspectives and new insights. Curr. Genomics 21, 343–362. doi: 10.2174/1389202921999200515140420, 33093798 PMC7536805

[ref150] Singh H. Yadav M. Kumar N. Kumar A. Kumar M. (2020). Assessing adaptation and mitigation potential of roadside trees under the influence of vehicular emissions: a case study of Grevillea robusta and *Mangifera indica* planted in an urban city of India. PLoS One 15:e0227380. doi: 10.1371/journal.pone.0227380, 31990922 PMC6986729

[ref151] Song N. Tian Y. Luo Z. Dai J. Liu Y. Duan Y. (2022). Advances in pretreatment and analysis methods of aromatic hydrocarbons in soil. RSC Adv. 12, 6099–6113. doi: 10.1039/d1ra08633b, 35424557 PMC8981609

[ref152] Song Y. Yao S. Li X. Wang T. Jiang X. Bolan N. . (2024). Soil metabolomics: deciphering underground metabolic webs in terrestrial ecosystems. Eco-Environ. Health 3, 227–237. doi: 10.1016/j.eehl.2024.03.001, 38680731 PMC11047296

[ref153] Sousa L. G. V. Castro J. Cavaleiro C. Salgueiro L. Tomás M. Palmeira-Oliveira R. . (2022). Synergistic effects of carvacrol, α-terpinene, γ-terpinene, ρ-cymene and linalool against Gardnerella species. Sci. Rep. 12:4417. doi: 10.1038/s41598-022-08217-w, 35292704 PMC8924259

[ref154] Staszel-Szlachta K. Lasota J. Szlachta A. Błońska E. (2024). The impact of root systems and their exudates in different tree species on soil properties and microorganisms in a temperate forest ecosystem. BMC Plant Biol. 24:45. doi: 10.1186/s12870-024-04724-2, 38212695 PMC10785385

[ref155] Štempelová L. Micenková L. Andrla P. Strompfová V. (2025). The skin microbiome on healthy and inflammatory altered canine skin determined by next generation sequencing. Front. Microbiol. 16:1528747. doi: 10.3389/fmicb.2025.1528747, 40083787 PMC11903403

[ref156] Stra A. Almarwaey L. O. Alagoz Y. Moreno J. C. Al-Babili S. (2023). Carotenoid metabolism: new insights and synthetic approaches. Front. Plant Sci. 13:1072061. doi: 10.3389/fpls.2022.1072061, 36743580 PMC9891708

[ref157] Tan X. Liu Y. Yan K. Wang Z. Lu G. He Y. . (2017). Differences in the response of soil dehydrogenase activity to cd contamination are determined by the different substrates used for its determination. Chemosphere 169, 324–332. doi: 10.1016/j.chemosphere.2016.11.076, 27886534

[ref158] Tang A. Haruna A. O. Majid N. M. A. Jalloh M. B. (2020). Potential PGPR properties of cellulolytic, nitrogen-fixing, phosphate-solubilizing Bacteria in rehabilitated tropical Forest soil. Microorganisms 8:442. doi: 10.3390/microorganisms8030442 32245141 PMC7143980

[ref159] Tuzen M. (2003). Determination of heavy metals in soil, mushroom and plant samples by atomic absorption spectrometry. Microchem. J. 74, 289–297. doi: 10.1016/S0026-265X(03)00035-3

[ref160] Ueberschaar N. Lehmann K. Meyer S. Zerfass C. Michalzik B. Totsche K. U. . (2021). Soil solution analysis with untargeted GC–MS—A case study with different lysimeter types. Front. Earth Sci. 8:563379. doi: 10.3389/feart.2020.563379

[ref161] Ukabiala M. E. Kolo J. Obalum S. E. Amhakhian S. O. Igwe C. A. Hermensah (2021). Physicochemical properties related to the mineralogical composition of floodplain soils in humid tropical environments and the pedological significance. Environ. Monit. Assess. 193:569. doi: 10.1007/s10661-021-09329-y, 34386866

[ref162] Uroz S. Oger P. Tisserand E. Cébron A. Turpault M. P. Buée M. . (2016). Specific impacts of beech and Norway spruce on the structure and diversity of the rhizosphere and soil microbial communities. Sci. Rep. 6:27756. doi: 10.1038/srep27756, 27302652 PMC4908602

[ref163] Valadares R. V. Costa M. D. Neves J. C. L. Vieira Netto J. A. F. Silva I. R. D. Moro E. . (2020). Rhizosphere microbiological processes and eucalypt nutrition: synthesis and conceptualization. Sci. Total Environ. 746:141305. doi: 10.1016/j.scitotenv.2020.141305 32771762

[ref164] van Dam N. M. Bouwmeester H. J. (2016). Metabolomics in the rhizosphere: tapping into belowground chemical communication. Trends Plant Sci. 21, 256–265. doi: 10.1016/j.tplants.2016.01.008 26832948

[ref165] Vasanthrao R. Nidhin I. K. Taj Z. Chattopadhyay I. (2025). Comprehensive whole metagenomics analysis uncovers microbial community and resistome variability across anthropogenically contaminated soils in urban and suburban areas of Tamil Nadu, India. Front. Microbiol. 16:1649872. doi: 10.3389/fmicb.2025.1649872 41199948 PMC12586116

[ref166] Veach A. M. Chen H. Yang Z. K. Labbe A. D. Engle N. L. Tschaplinski T. J. . (2020). Plant hosts modify belowground microbial community response to extreme drought. mSystems 5:e00092-20. doi: 10.1128/mSystems.00092-20, 32606021 PMC7329318

[ref167] Wang X. Ma B. Liu H. Bao Y. Li M. McLaughlin N. B. . (2023). Improvement in gravel-mulched land soil nutrient and bacterial community diversity with *Lonicera japonica*. Front. Microbiol. 14:1225503. doi: 10.3389/fmicb.2023.1225503 38130947 PMC10733477

[ref168] Wang Y. Sun X. Li S. Wei B. (2024). Lignin and cellulose contents in Chinese red pine (Pinustabuliformis Carr.) plantations varied in stand structure, soil property, and regional climate. Forests 15:240. doi: 10.3390/f15020240

[ref169] Ward N. L. Challacombe J. F. Janssen P. H. Henrissat B. Coutinho P. M. Wu M. . (2009). Three genomes from the phylum Acidobacteria provide insight into the lifestyles of these microorganisms in soils. Appl. Environ. Microbiol. 75, 2046–2056. doi: 10.1128/AEM.02294-08, 19201974 PMC2663196

[ref170] Wei L. Wang Y. Li N. Zhao N. Xu S. (2024). Bacteria-like *Gaiella* accelerate soil carbon loss by decomposing organic matter of grazing soils in alpine meadows on the Qinghai-Tibet plateau. Microb. Ecol. 87:104. doi: 10.1007/s00248-024-02414-y 39110233 PMC11306262

[ref171] Williams M. C. Wardle G. M. (2007). Pine and eucalypt litterfall in a pine-invaded eucalypt woodland: the role of fire and canopy cover. For. Ecol. Manag. 253, 1–10. doi: 10.1016/j.foreco.2007.06.045

[ref172] Wood D. E. Lu J. Langmead B. (2019). Improved metagenomic analysis with kraken 2. Genome Biol. 20:257. doi: 10.1186/s13059-019-1891-0, 31779668 PMC6883579

[ref173] Wu Y. Wang H. Peng L. Zhao H. Zhang Q. Tao Q. . (2024). Root-soil-microbiome interaction in the rhizosphere of Masson pine (*Pinus massoniana*) under different levels of heavy metal pollution. Ecotoxicol. Environ. Saf. 283:116779. doi: 10.1016/j.ecoenv.2024.116779, 39083870

[ref174] Wu L. Wang J. Wu H. Chen J. Xiao Z. Qin X. . (2018). Comparative metagenomic analysis of rhizosphere microbial community composition and functional potentials under *Rehmannia glutinosa* consecutive monoculture. Int. J. Mol. Sci. 19:2394. doi: 10.3390/ijms19082394, 30110928 PMC6121535

[ref175] Wu L. Weston L. A. Zhu S. Zhou X. (2023). Editorial: rhizosphere interactions: root exudates and the rhizosphere microbiome. Front Plant Sci. 14:1281010. doi: 10.3389/fpls.2023.1281010, 37736613 PMC10509041

[ref176] Xia Z. Bai E. Wang Q. Gao D. Zhou J. Jiang P. . (2016). Biogeographic distribution patterns of Bacteria in typical Chinese Forest soils. Front. Microbiol. 7:1106. doi: 10.3389/fmicb.2016.01106, 27468285 PMC4942481

[ref177] Xia F. Ou-Yang T. N. Gao Z. H. Qiu L. H. (2018). *Edaphobacter flagellatus* sp. nov. and *Edaphobacter bradus* sp. nov., two acidobacteria isolated from forest soil. Int. J. Syst. Evol. Microbiol. 68, 2530–2537. doi: 10.1099/ijsem.0.002871, 29957171

[ref178] Xu Q. Fu H. Zhu B. Hussain H. A. Zhang K. Tian X. . (2021). Potassium improves drought stress tolerance in plants by affecting root morphology, root exudates and microbial diversity. Metabolites. 11:131. doi: 10.3390/metabo11030131, 33668385 PMC7996290

[ref179] Xu Y. Li J. Qiao C. Yang J. Li J. Zheng X. . (2024). Rhizosphere bacterial community is mainly determined by soil environmental factors, but the active bacterial diversity is mainly shaped by plant selection. BMC Microbiol. 24:450. doi: 10.1186/s12866-024-03611-y, 39501158 PMC11536854

[ref180] Yang Y. Li Y. Hao K. Zhao Y. Li M. Fan Y. (2024). Microbial community composition and co-occurrence network analysis of the rhizosphere soil of the main constructive tree species in Helan Mountain of Northwest China. Sci. Rep. 14:24557. doi: 10.1038/s41598-024-76195-2, 39427091 PMC11490567

[ref181] Yanık-Yıldırım K. C. Vardar-Schara G. (2014). Saturation mutagenesis of Bradyrhizobium sp. BTAi1 toluene 4-monooxygenase at alpha-subunit residues proline 101, proline 103, and histidine 214 for regiospecific oxidation of aromatics. Appl. Microbiol. Biotechnol. 98, 8975–8986. doi: 10.1007/s00253-014-5913-2, 25016343

[ref182] Yin Y. R. Sang P. Xian W. D. Li X. Jiao J. Y. Liu L. . (2018). Expression and characteristics of two glucose-tolerant GH1 β-glucosidases from *Actinomadura amylolytica* YIM 77502T for promoting cellulose degradation. Front. Microbiol. 9:3149. doi: 10.3389/fmicb.2018.03149, 30619214 PMC6305311

[ref183] Yuan S. F. Yue X. J. Hu W. F. Wang Y. Li Y. Z. (2023). Genome-wide analysis of lipolytic enzymes and characterisation of a high-tolerant carboxylesterase from *Sorangium cellulosum*. Front. Microbiol. 14:1304233. doi: 10.3389/fmicb.2023.1304233, 38111649 PMC10725956

[ref184] Zhang X. Gai X. Zhong Z. Bian F. Yang C. Li Y. . (2021). Understanding variations in soil properties and microbial communities in bamboo plantation soils along a chromium pollution gradient. Ecotoxicol. Environ. Saf. 222:112507. doi: 10.1016/j.ecoenv.2021.112507 34265530

[ref185] Zhang Q. Gao M. Sun X. Wang Y. Yuan C. Sun H. (2023a). Nationwide distribution of polycyclic aromatic hydrocarbons in soil of China and the association with bacterial community. J. Environ. Sci. (China) 128, 1–11. doi: 10.1016/j.jes.2022.07.026, 36801025

[ref186] Zhang S. Landuyt D. Verheyen K. De Frenne P. (2022). Tree species mixing can amplify microclimate offsets in young forest plantations. J. Appl. Ecol. 59, 1428–1439. doi: 10.1111/1365-2664.14158

[ref187] Zhang L. Mi X. Harrison R. D. Yang B. Man X. Ren H. . (2020). Resource heterogeneity, not resource quantity, plays an important role in determining tree species diversity in two species-rich forests. Front. Ecol. Evol. 8:224. doi: 10.3389/fevo.2020.00224

[ref188] Zhang S. Pan Y. G. Zheng L. Yang Y. Zheng X. Ai B. . (2019). Application of steam explosion in oil extraction of camellia seed (*Camellia oleifera* Abel.) and evaluation of its physicochemical properties, fatty acid, and antioxidant activities. Food Sci. Nutr. 7, 1004–1016. doi: 10.1002/fsn3.924, 30918643 PMC6418447

[ref189] Zhang N. Yang D. Wang D. Miao Y. Shao J. Zhou X. . (2015). Whole transcriptomic analysis of the plant-beneficial rhizobacterium Bacillus amyloliquefaciens SQR9 during enhanced biofilm formation regulated by maize root exudates. BMC Genomics 16:685. doi: 10.1186/s12864-015-1825-5 26346121 PMC4562157

[ref190] Zhang Q. Zhang Y. Wang Y. Lin S. Chen M. Cheng P. . (2023b). Effects of pruning on tea tree growth, tea quality, and rhizosphere soil microbial community. Microbiol. Spectrum 11:e0160123. doi: 10.1128/spectrum.01601-23, 37750694 PMC10655597

[ref191] Zheng F. Chen L. Gao J. Niu F. Duan X. Yin L. . (2018). Identification of autotoxic compounds from *Atractylodes macrocephala* Koidz and preliminary investigations of their influences on immune system. J. Plant Physiol. 230, 33–39. doi: 10.1016/j.jplph.2018.08.006 30144693

[ref192] Zheng F. Gu J. Lu D. Yang J. Shuai X. Li C. . (2024). Mixing with native broadleaf trees modified soil microbial communities of *Cunninghamia lanceolata* monocultures in South China. Front. Microbiol. 15:1372128. doi: 10.3389/fmicb.2024.1372128, 38505544 PMC10949948

[ref193] Zhou Y. Mu M. Yang M. Yang X. Zhang H. Guo D. . (2022). The rhizospheric bacterial diversity of *Fritillaria taipaiensis* under single planting pattern over five years. Sci. Rep. 12:22544. doi: 10.1038/s41598-022-26810-x, 36581656 PMC9800406

[ref194] Zhuang Y. Wang H. Tan F. Wu B. Liu L. Qin H. . (2024). Rhizosphere metabolic cross-talk fromplant-soil-microbe tapping into agricultural sustainability: current advance and perspectives. Plant Physiol. Biochem. 210:108619. doi: 10.1016/j.plaphy.2024.108619, 38604013

[ref195] Zinn Y. L. Resck D. V. da Silva J. E. (2002). Soil organic carbon as affected by afforestation with Eucalyptus and Pinus in the Cerrado region of Brazil. For. Ecol. Manag. 166, 285–294. doi: 10.1016/S0378-1127(01)00682-X

[ref196] Zitzelsberger C. Buchbauer G. (2015). Essential oils as "a cry for help". A review. Nat Prod Commun. 10, 1127–1138. 26197563

[ref197] Zuo Y. W. Liu Y. Y. Jiang Y. X. Li W. Q. Peng Y. Zhou S. M. . (2025). Improving soil properties and microbiomes by mixed Eucalyptus-Cupressus afforestation. Biology 14:1667. doi: 10.3390/biology14121667, 41463440 PMC12729669

