# Supplementary material for: Integrated study of rhizosphere microbiome and metabolome profiles across tropical plantations in the Western Ghat regions of Kerala and Tamil Nadu, India
Source: Front Microbiol. 2026 May 13;17:1840024. doi: 10.3389/fmicb.2026.1840024 (PMC13212104; doi:10.3389/fmicb.2026.1840024)
Supplement: Supplementary file 1 [file Supplementary_file_1.docx]

**Supplementary tables**

**Supplementary Table 2.** Soil physiochemical parameters and heavy metal concentrations detected across the samples

|  | CM1 | CM2 | CM3 | TM1 | TM2 | TM3 | RM1 | RM2 | RM3 | EXP1 | PXE1 | PM1 | EM1 | SXT1 | TXS1 | SXE1 | EXS1 |
| --- | --- | --- | --- | --- | --- | --- | --- | --- | --- | --- | --- | --- | --- | --- | --- | --- | --- |
| Plantation type | Mono-species | Mono-species | Mono-species | Mono-species | Mono-species | Mono-species | Mono-species | Mono-species | Mono-species | Mixed-species | Mixed-species | Mono-species | Mono-species | Mixed-species | Mixed-species | Mixed-species | Mixed-species |
| Plant genera | Coffee | Coffee | Coffee | Tea | Tea | Tea | Rubber | Rubber | Rubber | Eucaplyptus | Pine | Pine | Eucaplyptus | Silver oak | Tea | Silver oak | Eucaplyptus |
| Plant type | Shrubs | Shrubs | Shrubs | Shrubs | Shrubs | Shrubs | Trees | Trees | Trees | Trees | Trees | Trees | Trees | Trees | Shrubs | Trees | Trees |
| N  (Kg/ha) | 208 | 128.7 | 296 | 258.5 | 240.4 | 316.1 | 324.7 | 358.7 | 287.5 | 302 | 258 | 315 | 315 | 334 | 214 | 287 | 211 |
| P  (Kg/ha) | 57 | 86.5 | 55.9 | 33.4 | 38.72 | 52.12 | 26.35 | 34.5 | 17.19 | 20.7 | 13.2 | 18.5 | 15.2 | 586 | 3.74 | 9.99 | 2.74 |
| AN  (mg/kg) | 111 | 44.7 | 79.3 | 144.2 | 123.3 | 189.3 | 175.5 | 643 | 102.4 | 201.6 | 156.8 | 235 | 179 | 202 | 157 | 246 | 168 |
| NN  (mg/kg) | 2.1 | 8 | 8.7 | 7.3 | 6.8 | 13 | 3.45 | 2.71 | 44.73 | 8.27 | 9.47 | 12.7 | 10.1 | 8.27 | 5.28 | 7.891 | 8.547 |
| AP  (mg/kg) | 95 | 4141 | 1560 | 2671 | 459 | 831 | 616 | 1235 | 628 | 3593 | 1695 | 2074 | 10005 | 586 | 3178 | 2489 | 4704 |
| TOC  (%) | 0.57 | 1.68 | 0.95 | 1.506 | 1.37 | 1.87 | 1.33 | 0.078 | 0.12 | 7.27 | 7.21 | 1.34 | 1.81 | 0.41 | 0.06 | 0.97 | 0.37 |
| K  (Kg/ha) | 204.5 | 119.3 | 281 | 239.3 | 204.2 | 295 | 269 | 312 | 223 | 251 | 138 | 257 | 356 | 214 | 106 | 92.1 | 73.9 |
| Na  (Kg/ha) | 34.04 | 30.24 | 58.24 | 32.48 | 29.12 | 40.32 | 39.2 | 29.12 | 137 | 76.6 | 122 | 355 | 252 | 399 | 276 | 140 | 283 |
| Ca  (Meq/100 gm) | 599.3 | 577 | 586.5 | 987 | 756 | 583 | 560 | 584 | 786 | 1372 | 1 | 784 | 392 | 78.4 | 1 | 588 | 196 |
| Mg  (Meq/100gm) | 971 | 239.5 | 961 | 478 | 240 | 240 | 243 | 240 | 723 | 595 | 1 | 238 | 119 | 95.3 | 1 | 357 | 71.4 |
| Pb  (ppm) | 0.712 | 3.22 | 4.92 | 3.89 | 2.21 | 4.66 | 4.73 | 5.81 | 1.73 | 0.987 | 0.814 | 1.021 | 1.174 | 1.14 | 0.847 | 0.413 | 0.104 |
| Ni  (ppm) | 0.04 | 0.09 | 0.11 | 0.12 | 0.19 | 0.77 | 0.03 | 0.79 | 0.18 | 0.004 | 0.016 | 0.432 | 0.51 | 0.127 | 0.001 | 0.001 | 0.001 |
| Cu  (ppm) | 0.97 | 0.88 | 1.44 | 4.43 | 1.573 | 10.26 | 1.277 | 1.76 | 3.58 | 1.574 | 0.987 | 1.174 | 1.03 | 0.417 | 0.134 | 0.101 | 0.001 |
| Zn  (ppm) | 0.88 | 1.56 | 2.76 | 9.174 | 7.432 | 28.3 | 11.3 | 13.6 | 14.2 | 32.7 | 27.9 | 42.7 | 38.4 | 40.7 | 26.7 | 32.4 | 18.7 |
| Al  (ppm) | 15171 | 28249 | 46393 | 64367 | 93456 | 82498 | 45455 | 44022 | 93904 | 232347 | 179044 | 100106 | 41979 | 218471 | 225942 | 198815 | 170047 |
| Fe  (ppm) | 78.69 | 88.63 | 91.42 | 83.61 | 98.65 | 92 | 63.5 | 96.1 | 101.4 | 214 | 229 | 389 | 427 | 318 | 210 | 189 | 147 |
| Mn  (ppm) | 310.88 | 335.3 | 281.3 | 534.96 | 555.84 | 593.06 | 314.8 | 285.23 | 371.81 | 283.6 | 268.58 | 258.4 | 219.91 | 911.62 | 623.19 | 639.5 | 426.93 |
| Cr  (ppm) | 0.71 | 0.092 | 0.096 | 0.099 | 1.024 | 1.08 | 0.78 | 1.06 | 0.57 | 0.178 | 0.117 | 0.078 | 0.043 | 0.017 | 0.001 | 0.001 | 0.001 |
| Cd  (ppm) | 0.23 | 0.017 | 0.021 | 0.011 | 0.032 | 0.654 | 0.22 | 0.173 | 0.14 | 0.001 | 0.013 | 0.147 | 0.112 | 0.074 | 0.087 | 0.147 | 0.113 |
| Soil pH | 6.8 | 6.45 | 6.59 | 6.13 | 6.26 | 5.13 | 5.56 | 5.74 | 5.62 | 7.577 | 7.239 | 7.096 | 7.327 | 7.827 | 7.581 | 7.864 | 7.735 |

**Supplementary table 1**: The wavelengths used to measure the absorbance for the AAS analysis for different Heavy metals

| Sl. No. | Heavy metals | Wavelength | Reference |
| --- | --- | --- | --- |
| 1. | Al | 309.3 nm | Frankowski et al., 2011 |
| 2. | Pb | 283.3 nm | Tüzen, 2003 |
| 3. | Cr | 357.9 nm |  |
| 4. | Ni | 394.5 nm |  |
| 5. | Cd | 228.8 nm |  |
| 6. | Zn | 213.8 nm | https://agriculture. uk.gov.in /files/ Soil_ Testing_Method _by_Govt_of_India.pdf |

**Supplementary table 3**: Table depicting the sample group-wise mean soil pH values with standard deviation.

| Sample group | soil pH | S.D. |
| --- | --- | --- |
| Mono-species | 6.24 | 0.69 |
| Mixed-species | 7.64 | 0.23 |
| Coonoor | 7.53 | 0.28 |
| Kerala | 6.03 | 0.55 |
| Shrubs | 6.42 | 0.74 |
| Trees | 6.96 | 0.94 |
| Coffee | 6.61 | 0.18 |
| Tea | 6.28 | 0.62 |
| Rubber | 5.64 | 0.09 |
| Eucalyptus | 7.55 | 0.21 |
| Pine | 7.17 | 0.1 |
| Silver oak | 7.85 | 0.03 |

**Supplementary table 4**: List of BIS (Bureau of Indian Standards for heavy metal) standards and with the background levels of Indian soils limits (Rangasamy and Muniyandi, 2025) to analyse the potential contamination.

| **Sl. No.** | **Heavy metals** | **Background levels of Indian soils (mg/kg)** | **BIS (Bureau of Indian Standards for heavy metal) standards (mg/kg)** | **Samples exceeding Background levels of Indian soils (ppm)**  **1 ppm=1 mg/kg** |
| --- | --- | --- | --- | --- |
| 1. | Cd | 0.38 | 3 | TM3 |
| 2. | Pb | 13 | 250 | - |
| 3. | Cr | 114 | - | - |
| 4. | Cu | 56 | - | - |
| 5. | Ni | 27 | 75 | - |
| 6. | Mn | 209 | - | - |
| 7. | Fe | 32015 | - | - |
| 8. | Zn | 22 | 200 | TM3, PM1, EM1, PXE1, EXP1, SXT1, TXS1, SXE1 |
| 9. | Al | - | - | - |

**Supplementary table 5.** The chemical categories (classified based on Classyfire) and significance of the major metabolites detected across the samples.

| **Sl. No.** | **Compound Name** | **CAS#** | **Formula** | **Classification (Classyfire)** | **Significance (Pubchem - ChEBI and LOTUS)** |
| --- | --- | --- | --- | --- | --- |
| 1 | n-Propyl acetate | 109-60-4 | C_5_H_10_O_2_ | Carboxylic acid ester | Propyl acetate is a natural product found in *Zingiber mioga, Saussureainvolucrata*, and other organisms with data available. |
| 2 | Tetrachloroethylene | 127-18-4 | C_2_Cl_4_ | Vinyl chlorides | Tetrachloroethylene is a natural product found in *Gossypium hirsutum* with data available. |
| 3 | 1-Hexadecanol | 36653-82-4 | C_16_H_34_O | Long-chain fatty alcohols | 1-Hexadecanol is a natural product found in *Camellia sinensis, Angelica gigas*, and other organisms with data available. |
| 4 | Phthalic acid, di(2-propylpentyl) ester | 1000377-93-5 | C_24_H_38_O_4_ | benzoate ester | NA |
| 5 | Behenic alcohol | 661-19-8 | C_22_H_46_O | Fatty alcohols | Docosanol is a natural product found in *Mandragora autumnalis, Hibiscus cannabinus*, and other organisms with data available. |
| 6 | Dodecane, 1-iodo- | 4292-19-7 | C_12_H_25_I | Organoiodides | NA |
| 7 | 2-Pentadecanone, 6,10,14-trimethyl- | 502-69-2 | C_18_H_36_O | Sesquiterpenoids | 6,10,14-Trimethylpentadecan-2-one is a natural product found in *Leea guineensis, Pinelliaternata*, and other organisms with data available. |
| 8 | 1-Decanol, 2-hexyl- | 2425-77-6 | C_16_H_34_O | Fatty alcohols | NA |
| 9 | 2,4-Di-tert-butylphenol | 96-76-4 | C_14_H_22_O | Phenylpropanes | 2,4-Di-tert-butylphenol has been reported in *Streptomyces antioxidans, Bacillus subtilis*, and other organisms with data available. |
| 10 | Toluene | 108-88-3 | C_7_H_8_ | Aromatic hydrocarbon | Toluene has been reported in *Basella alba, Zingiber mioga*, and other organisms with data available. |
| 11 | Heneicosane | 629-94-7 | C_21_H_44_ | Alkanes | Heneicosane is a natural product found in *Vanilla madagascariensis, Magnolia officinalis*, and other organisms with data available. |
| 12 | Dodecane, 4,6-dimethyl- | 61141-72-8 | C_14_H_30_ | Branched alkanes | It has a role as a human metabolite. |
| 13 | Nonane, 2,2,4,4,6,8,8-heptamethyl- | 909554 | C_16_H_34_ | Branched alkanes | NA |
| 14 | Squalene | 111-02-4 | C_30_H_50_ | triterpene | Squalene is a triterpene consisting of 2,6,10,15,19,23-hexamethyltetracosane having six double bonds at the 2-, 6-, 10-, 14-, 18- and 22-positions with (all-E)-configuration. It has a role as a human metabolite, a plant metabolite, a *Saccharomyces cerevisiae*metabolite and a mouse metabolite. |
| 15 | Hexadecane, 2,6,10,14-tetramethyl- | 638-36-8 | C_20_H_42_ | Acyclic diterpenoids | Phytane is a natural product found in *Capsicum annuum, Cynomoriumsongaricum,* and other organisms with data available. |
| 16 | Cyclohexane, ethyl- | 1678-91-7 | C_8_H_16_ | Cycloalkanes | Ethylcyclohexane is a natural product found in *Garcinia mangostana* and *Cynara cardunculus* with data available. |
| 17 | Hentriacontane | 630-04-6 | C_31_H_64_ | Alkanes | Hentriacontane is a natural product found in *Euphorbia piscatoria, Vanilla madagascariensis*, and other organisms with data available. |
| 18 | Nonane, 5-methyl-5-propyl- | 17312-75-3 | C_13_H_28_ | Branched alkanes | NA |
| 19 | .alpha.-Phellandrene, dimer | 7350-11-0 | C_20_H_32_ | monoterpenoids | NA |
| 20 | Oxirane, 2-(1,1-dimethylethyl)-3-ethyl-, cis- | 36099-44-2 | C_8_H_16_O | epoxides | NA |
| 21 | .alpha.-Phellandrene | 99-83-2 | C_10_H_16_ | Menthane monoterpenoids | alpha-Phellandrene is a natural product found in *Artemisia thuscula, Espeletiaweddellii*, and other organisms with data available. |
| 22 | benzoxazole, 2-(chloromethyl)- | 1000404-53-7 | C_8_H_6_ClNO | aliphatic chlorides | NA |
| 23 | 4,8,12,16-Tetramethylheptadecan-4-olide | 96168-15-9 | C_21_H_40_O_2_ | NA | NA |
| 24 | Hexanedioic acid, bis(2-ethylhexyl) ester | 103-23-1 | C_22_H_42_O_4_ | carboxylic diester | Bis(2-ethylhexyl) adipate has been reported in *Panax ginseng*with data available. |
| 25 | n-Tetracosanol-1 | 506-51-4 | C_24_H_50_O | Fatty alcohols | 1-Tetracosanol is a natural product found in *Castanopsis lanceifolia, Artemisia igniaria*, and other organisms with data available. |
| 26 | Hexadecane | 544-76-3 | C_16_H_34_ | Alkanes | Hexadecane is a natural product found in *Camellia sinensis, Vanilla madagascariensis*, and other organisms with data available. |
| 27 | Undecane, 3,8-dimethyl- | 17301-30-3 | C_13_H_28_ | Branched alkanes | NA |
| 28 | Butane, 1-propoxy- | 3073-92-5 | C_7_H_16_O | Dialkyl ethers | NA |
| 29 | Azulene, 1,2,3,3a,4,5,6,7-octahydro-1,4-dimethyl-7- (1.alpha.,3a.beta.,4.alpha.,7.beta.)]- | 22567-17-5 | C_15_H_24_ | Sesquiterpenoids | gamma-Gurjunene is a natural product found in *Calypogeiamuelleriana, Tetradenia riparia*, and other organisms with data available. |
| 30 | Eucalyptol | 470-82-6 | C_10_H_18_O | Monoterpenoid (Oxane) | Eucalyptol is a natural product found in *Curcuma xanthorrhiza, Baeckea frutescens*, and other organisms with data available. |
| 31 | (1S,2E,6E,10R)-3,7,11,11- | 24703-35-3 | C_15_H_24_ | Bicyclogermacrane and isolepidozane sesquiterpenoids | Bicyclogermacrene is a natural product found in *Callilepislaureola, Humulus lupulus*, and other organisms with data available. |
| 32 | Friedelan-3-one | 559-74-0 | C_30_H_50_O | pentacyclic triterpenoid | Friedelin has been reported in *Erythrophleumfordii, Phellinus pomaceus*, and other organisms with data available. |
| 33 | Octadecane | 593-45-3 | C_18_H_38_ | straight-chain alkane | Octadecane has been reported in *Camellia sinensis, Vanilla madagascariensis*, and other organisms with data available. |
| 34 | Hexadecane, 2,6,11,15-tetramethyl- | 504-44-9 | C_20_H_42_ | Terpenes | isoprenoid isomer of phytane found in methane-seep sediments and some Palaeozoic crude oils |
| 35 | Phenol, 2,4-bis(1,1-dimethylethyl)-, phosphite (3:1) | 31570-04-4 | C_42_H_63_O_3_P | alkylbenzene | NA |
| 36 | Eicosane, 1-iodo- | 1000406-31-8 | C_20_H_41_I | Organoiodides | NA |
| 37 | Sulfurous acid, 2-ethylhexyl hexyl ester | 1000309-20-2 | C_14_H_30_O_3_S | Organooxygen compounds | NA |
| 38 | Triacontane | 638-68-6 | C_30_H_62_ | Alkanes | Triacontane is a natural product found in *Vanilla madagascariensis, Echinacea angustifolia*, and other organisms with data available. |
| 39 | Octadecane, 1-iodo- | 629-93-6 | C_18_H_37_I | Organoiodides | NA |
| 40 | 7,9-Di-tert-butyl-1-oxaspiro(4,5)deca-6,9-diene-2,8- | 82304-66-3 | C_17_H_24_O_3_ | Gamma butyrolactones | 7,9-Di-tert-butyl-1-oxaspiro[4.5]deca-6,9-diene-2,8-dione is a natural product found in *Mangifera indica* with data available. |
| 41 | 1-Octadecanol | 112-92-5 | C_18_H_38_O | Long-chain fatty alcohols | Stearyl alcohol is a natural product found in *Camellia sinensis, Apis,* and other organisms with data available. |
| 42 | Docosanal | 57402-36-5 | C_22_H_44_O | Fatty aldehyde | NA |
| 43 | Decane, 3,8-dimethyl- | 17312-55-9 | C_12_H_26_ | Branched alkanes | NA |
| 44 | 1-Nonadecene | 18435-45-5 | C_19_H_38_ | Unsaturated aliphatic hydrocarbons | 1-Nonadecene is a natural product found in *Streptomyces, Ammodaucusleucotrichus*, and other organisms with data available. |
| 45 | Cyclotetradecane | 295-17-0 | C_14_H_28_ | Cycloalkanes | Cyclotetradecane is a natural product found in *Vitis vinifera* with data available. |
| 46 | Isopropyl acetate | 108-21-4 | C_5_H_10_O_2_ | Carboxylic acid esters | Isopropyl acetate is a natural product found in *Nicotiana tabacum* and *Solanum lycopersicum* with data available. |
| 47 | Acetic acid | 64-19-7 | C_2_H_4_O_2_ | Carboxylic acids | NA |
| 48 | (-)-Globulol | 489-41-8 | C_15_H_26_O | Guaiane sesquiterpenoid | (-)-Globulol is a natural product found in Salvia coccinea, *Humulus lupulus*, and other organisms with data available (Pubcem).*Eucalyptus albens* (whitebox*), Eucalyptus astringens* (brown mallet) (LOTUS - the natural products occurrence database) |
| 49 | Aromandendrene | 489-39-4 | C_15_H_24_ | 5,10-cycloaromadendrane sesquiterpenoids | Aromadendrene is a natural product found in *Vitex negundo, Guatteriablepharophylla,* and other organisms with data available. |
| 50 | 1H-Cyclopropa[a]naphthalene, 1a,2,3,5,6,7,7a,7b- (1a.alpha.,7.alpha.,7a.alpha.,7b.alpha.)]- | 17334-55-3 | C_15_H_24_ | Aristolane sesquiterpenoids | NA |
| 51 | o-Cymene | 527-84-4 | C_10_H_14_ | Cumenes | O-Cymene is a natural product found in *Cymbopogon martinii, Helichrysum taenari*, and other organisms with data available. |
| 52 | (1aR,4S,4aR,7R,7aS,7bS)-1,1,4,7-Tetramethyldecahydro-1H-cyclopropa[e]azulen-4-ol | 88728-58-9 | C_15_H_26_O | 5,10-cycloaromadendrane sesquiterpenoids | Epiglobulol is a natural product found in *Ligusticum striatum, Valeriana officinalis*, and other organisms with data available. |
| 53 | Bicyclo[3.1.0]hex-2-ene, 4-methyl-1-(1-methylethyl)- | 28634-89-1 | C_10_H_16_ | Bicyclic monoterpenoid | beta-Thujene is a natural product found in *Pinus longaeva, Elsholtziafruticosa*, and other organisms with data available. |
| 54 | Tris(2,4-di-tert-butylphenyl) phosphate | 95906-11-9 | C_42_H_63_O_4_P | aryl phosphate | Natural product found in *Vitex negundo* (huang ping) |
| 55 | A'-Neogammacer-22(29)-ene | 1615-91-4 | C_30_H_50_ | triterpene | Diploptene has been reported in *Goniophlebiumniponicum, Pyrrosiapetiolosa*, and other organisms with data available. |
| 56 | 2-Furancarboxylic acid, hydrazide | 3326-71-4 | C_5_H_6_N_2_O_2_ | Furans | NA |
| 57 | Benzene, 1,3-dimethyl- | 108-38-3 | C_8_H_10_ | m-Xylenes | m-Xylene is a natural product found in *Zingiber mioga, Helianthus tuberosus*, and other organisms with data available. |
| 58 | 3,4-Hexanedione | 4437-51-8 | C_6_H_10_O_2_ | alpha-diketone | NA |
| 59 | 3-Ethyl-2,6,10-trimethylundecane | 1000432-25-9 | C_16_H_34_ | sesquiterpenoid | NA |
| 60 | Silane, diethylheptyloxyoctadecyloxy- | 1000363-96-0 | C_29_H_62_O_2_Si | NA | NA |
| 61 | Phthalic acid, hept-4-yl isobutyl ester | 1000356-78-3 | C_19_H_28_O_4_ | Phthalic acids | NA |
| 62 | Octacosanol | 557-61-9 | C_28_H_58_O | Fatty alcohols | 1-Octacosanol is a natural product found in *Acanthus ilicifolius, Euphorbia piscatoria*, and other organisms with data available. |
| 63 | 3-Ethyl-3-methylheptane | 17302-01-1 | C_10_H_22_ | Branched alkanes | NA |
| 64 | Carbonic acid, 2-dimethylaminoethyl ethyl ester | 1000331-33-7 | C_7_H_15_NO_3_ | Carbonic acid diesters | NA |
| 65 | Methyl propionate | 554-12-1 | C_4_H_8_O_2_ | Methyl esters | Methyl propionate is a natural product found in *Durio zibethinus, Actinidia chinensis*, and other organisms with data available. |

**Supplementary table 6.** Alpha diversity indices (Observed, Chao1, Simpson, and Shannon) across the rhizosphere soil samples**.**

| **Sl. No.** | **Plantation_type** | **Plant_genera** | **Plant_type** | **Sampling location** | **Observed** | **Chao1** | **Simpson** | **Shannon** |
| --- | --- | --- | --- | --- | --- | --- | --- | --- |
| 1 | Mono-species | Pine | Trees | Coonoor | 89 | 97.25 | 0.867992 | 2.664024 |
| 2 | Mixed-species | Pine | Trees | Coonoor | 142 | 147 | 0.910857 | 3.187136 |
| 3 | Mono-species | Eucalyptus | Trees | Coonoor | 146 | 148 | 0.963508 | 3.888166 |
| 4 | Mixed-species | Eucalyptus | Trees | Coonoor | 86 | 90.58333 | 0.883541 | 2.624097 |
| 5 | Mixed-species | Eucalyptus | Trees | Coonoor | 145 | 146.4286 | 0.942324 | 3.675564 |
| 6 | Mixed-species | Silver_oak | Trees | Coonoor | 150 | 150.75 | 0.938474 | 3.579303 |
| 7 | Mixed-species | Silver_oak | Trees | Coonoor | 147 | 147 | 0.954992 | 3.801399 |
| 8 | Mixed-species | Tea | Shrubs | Coonoor | 150 | 151 | 0.964555 | 3.945369 |
| 9 | Mono-species | Coffee | Shrubs | Kerala | 228 | 228.6 | 0.964378 | 3.995805 |
| 10 | Mono-species | Coffee | Shrubs | Kerala | 231 | 231.25 | 0.963717 | 4.100368 |
| 11 | Mono-species | Coffee | Shrubs | Kerala | 219 | 230.1429 | 0.967549 | 4.049697 |
| 12 | Mono-species | Rubber | Trees | Kerala | 185 | 218.0556 | 0.947409 | 3.719377 |
| 13 | Mono-species | Rubber | Trees | Kerala | 230 | 230.6 | 0.960456 | 4.002358 |
| 14 | Mono-species | Rubber | Trees | Kerala | 225 | 226 | 0.973528 | 4.245358 |
| 15 | Mono-species | Tea | Shrubs | Kerala | 208 | 223.4 | 0.953684 | 3.81778 |
| 16 | Mono-species | Tea | Shrubs | Kerala | 209 | 215.3158 | 0.955983 | 3.797159 |
| 17 | Mono-species | Tea | Shrubs | Kerala | 4 | 4 | 0.749996 | 1.386287 |

**Supplementary table 7.** Pearson correlation between soil physiochemical parameters, metabolites, and bacterial taxa. Correlations withPearson correlation coefficient(r) greater than 0.7 (0.7 to 1) and less than -0.7 (-0.7 to -1) are given in the table

| **Node name (1)** | **Node name (2)** | **Pearson correlation value (r)** | **p-value** |
| --- | --- | --- | --- |
| Phosphorous | 1-Decanol, 2-hexyl- | 0.79301 | 0.00014615 |
| Available Nitrogen | *Nocardia* | 0.81561 | 0.000065877 |
| Available Nitrogen | *Acidiferrimicrobium australe* | 0.70086 | 0.001724 |
| Available Phosphorus | 3-Ethyl-3-methylheptane | 0.71271 | 0.001323 |
| Available Phosphorus | *Streptomyces* | 0.73723 | 0.0007334 |
| Available Phosphorus | *Luteitalea pratensis* | 0.79554 | 0.00013434 |
| Available Phosphorus | *Actinobacteria bacterium* IMCC26256 | 0.79172 | 0.00015252 |
| Available Phosphorus | *Hyphomicrobium* | 0.7577 | 0.00042609 |
| Available Phosphorus | *Rhodoplanes* sp. Z2-YC6860 | 0.72337 | 0.0010313 |
| Available Phosphorus | *Haliangiumochraceum* | 0.7585 | 0.00041668 |
| Available Phosphorus | *Mesorhizobium* | 0.70905 | 0.0014378 |
| Available Phosphorus | *Chryseolinea soli* | 0.76984 | 0.00030112 |
| Available Phosphorus | *Legionella* | 0.78271 | 0.0002037 |
| Available Phosphorus | *Bacillus simplex* | 0.74363 | 0.00062208 |
| Total Organic Carbon | Behenic alcohol | 0.94263 | 1.52E-08 |
| Total Organic Carbon | n-Tetracosanol-1 | 0.85134 | 1.46E-05 |
| Total Organic Carbon | gamma-Gurjunene | 0.79152 | 0.00015351 |
| Total Organic Carbon | Eucalyptol | 0.71706 | 0.0011967 |
| Total Organic Carbon | Docosanal | 0.89137 | 3.58E-06 |
| Total Organic Carbon | (-)-Globulol | 0.86329 | 8.0852E-06 |
| Total Organic Carbon | Aromandendrene | 0.72945 | 0.00089017 |
| Total Organic Carbon | Epiglobulol | 0.84574 | 1.90E-05 |
| Total Organic Carbon | beta-Thujene | 0.93459 | 3.98E-08 |
| Total Organic Carbon | *Candidatus Xiphinematobacter*sp. Idaho Grape | 0.88142 | 2.9393E-06 |
| Total Organic Carbon | *Actinomaduraamylolytica* | 0.76436 | 0.00035308 |
| Sodium | Zinc | 0.73151 | 0.00084614 |
| Sodium | Iron | 0.79431 | 0.00013997 |
| Sodium | Oxirane, 2-(1,1-dimethylethyl)-3-ethyl-, cis- | 0.7199 | 0.0011197 |
| Sodium | *Gemmatimonas phototrophica* | 0.7003 | 0.001745 |
| Sodium | *Candidatus Koribacterversatilis* | 0.79591 | 0.00013269 |
| Sodium | *Sorangiumcellulosum* | 0.74175 | 0.00065321 |
| Sodium | *Paraburkholderia* | 0.83665 | 2.83E-05 |
| Sodium | *Bacillus* | 0.74989 | 0.00052733 |
| Lead | 3-Ethyl-2,6,10-trimethylundecane | 0.72202 | 0.001065 |
| Lead | Octadecane | 0.71904 | 0.0011425 |
| Lead | Toluene | 0.74272 | 0.00063707 |
| Lead | 2,4-Di-tert-butylphenol | 0.73556 | 0.00076485 |
| Lead | Tetrachloroethylene | 0.75332 | 0.00048065 |
| Zinc | *Acidisarcina polymorpha* | 0.72647 | 0.00095733 |
| Zinc | *Phenylobacterium*sp. HYN0004 | 0.70481 | 0.0015806 |
| Zinc | *Acidibrevibacteriumfodinaquatile* | 0.76088 | 0.00038977 |
| Zinc | *Candidatus Koribacterversatilis* | 0.78689 | 0.00017839 |
| Zinc | *Gemmataobscuriglobus* | 0.72053 | 0.0011033 |
| Zinc | *Candidatus Solibacterusitatus* | 0.73117 | 0.0008534 |
| Zinc | *Paludisphaera borealis* | 0.75561 | 0.00045148 |
| Zinc | Acetic acid | 0.75403 | 0.00047138 |
| Zinc | Isopropyl acetate | 0.77746 | 0.00023964 |
| Zinc | Oxirane, 2-(1,1-dimethylethyl)-3-ethyl-, cis- | 0.733 | 0.00081542 |
| Zinc | Iron | 0.85477 | 1.24E-05 |
| Aluminium | Oxirane, 2-(1,1-dimethylethyl)-3-ethyl-, cis- | 0.7658 | 0.00033876 |
| Aluminium | *Candidatus Solibacterusitatus* | 0.7355 | 0.00076602 |
| Aluminium | *Gemmataobscuriglobus* | 0.7329 | 0.00081758 |
| Aluminium | Candidatus Koribacterversatilis | 0.7823 | 0.00020632 |
| Aluminium | *Phenylobacterium* sp. HYN0004 | 0.73629 | 0.00075103 |
| Iron | Isopropyl acetate | 0.86641 | 6.87E-06 |
| Iron | Acetic acid | 0.82475 | 4.63E-05 |
| Iron | *Paludisphaera borealis* | 0.71601 | 0.0012263 |
| Iron | *Gemmataobscuriglobus* | 0.70514 | 0.0015689 |
| Iron | *Candidatus Koribacterversatilis* | 0.74851 | 0.00054707 |
| Iron | *Sorangiumcellulosum* | 0.75556 | 0.000452 |
| Iron | *Acidibrevibacteriumfodinaquatile* | 0.74712 | 0.00056771 |
| Iron | *Rhodoplanes sp.* Z2-YC6860 | 0.72643 | 0.00095813 |
| Iron | *Kribbellaflavida* | 0.71648 | 0.001213 |
| Iron | *Bacillus simplex* | 0.71565 | 0.0012365 |
| Chromium | Toluene | 0.70693 | 0.0015078 |
| Cadmium | *Serratia* | 0.77909 | 0.00022796 |
| Cadmium | *Phocaeicola* | 0.81324 | 7.20E-05 |
| Cadmium | *Lactococcus* | 0.87212 | 5.03E-06 |
| Cadmium | *Streptococcus* | 0.87464 | 4.37E-06 |
| Cadmium | *Morganella morganii* | 0.87184 | 5.11E-06 |
| Cadmium | *Lactobacillus* | 0.87937 | 3.32E-06 |
| Cadmium | *Butyricimonasvirosa* | 0.87852 | 3.49E-06 |
| Cadmium | *Dialister* | 0.87593 | 4.0582E-06 |
| Soil pH | Oxirane, 2-(1,1-dimethylethyl)-3-ethyl-, cis- | 0.87403 | 4.52E-06 |
| Soil pH | *Candidatus Solibacterusitatus* | 0.8479 | 1.72E-05 |
| Soil pH | *Gemmataobscuriglobus* | 0.84714 | 1.78E-05 |
| Soil pH | *Gemmatimonas phototrophica* | 0.75982 | 0.00040158 |
| Soil pH | *Candidatus Koribacterversatilis* | 0.74347 | 0.00062479 |
| Soil pH | *Sorangiumcellulosum* | 0.71864 | 0.0011534 |
| Soil pH | *Hypericibacter terrae* | 0.73327 | 0.00081007 |
| Soil DHA | Tetrachloroethylene | 0.78739 | 0.00017558 |
| Soil DHA | Toluene | 0.79113 | 0.00015547 |
| Soil DHA | Squalene | 0.75223 | 0.00049501 |
| Soil DHA | benzoxazole, 2-(chloromethyl)- | 0.8729 | 4.82E-06 |
| Soil DHA | Silane, diethylheptyloxyoctadecyloxy- | 0.76649 | 0.00033206 |
| oxirane, 2-(1,1-dimethylethyl)-3-ethyl-, cis-, | *Candidatus Solibacterusitatus* | 0.95347 | 3.26E-09 |
| Tetrachloroethylene | *Candidatus Solibacterusitatus* | -0.94495 | 1.12E-08 |
| Toluene | *Candidatus Solibacterusitatus* | -0.94969 | 5.80E-09 |
| Toluene | oxirane, 2-(1,1-dimethylethyl)-3-ethyl-, cis-, | -0.95946 | 1.18E-09 |
| Tetrachloroethylene | oxirane, 2-(1,1-dimethylethyl)-3-ethyl-, cis-, | -0.95462 | 2.72E-09 |
| Friedelan-3-one | *Reyranella soli* | 0.81451 | 6.87E-05 |
| Hexadecane, 2,6,11,15-tetramethyl- | *Gemmatimonas* | 0.70243 | 0.0016656 |
| Hexadecane, 2,6,11,15-tetramethyl- | *Ktedonobacter* | 0.7008 | 0.0017263 |
| Hexadecane, 2,6,11,15-tetramethyl- | *Conexibacter* | 0.7538 | 0.00047443 |
| Hexadecane, 2,6,11,15-tetramethyl- | *Reyranella* | 0.70249 | 0.0016636 |
| Hexadecane, 2,6,11,15-tetramethyl- | *Anaeromyxobacterdehalogenans* | 0.76791 | 0.00031862 |
| Hexadecane, 2,6,11,15-tetramethyl- | *Bradyrhizobium* | 0.8209 | 5.38E-05 |
| 3-Ethyl-3-methylheptane | *Luteitalea pratensis* | 0.79854 | 0.00012139 |
| 3-Ethyl-3-methylheptane | *Actinobacteria bacterium* IMCC26256 | 0.78156 | 0.00021116 |
| 3-Ethyl-3-methylheptane | *Hyphomicrobium* | 0.73652 | 0.00074659 |
| 3-Ethyl-3-methylheptane | *Haliangiumochraceum* | 0.7796 | 0.00022442 |
| 3-Ethyl-3-methylheptane | *Chryseolinea soli* | 0.83981 | 2.47E-05 |
| 3-Ethyl-3-methylheptane | *Bacillus simplex* | 0.82207 | 0.00005144 |
| Hexadecane, 2,6,10,14-tetramethyl- | *Chthoniobacter flavus* | 0.76602 | 0.00033663 |
| Hexadecane, 2,6,10,14-tetramethyl- | *Tepidisphaera mucosa* | 0.76169 | 0.00038103 |
| Hexadecane, 2,6,10,14-tetramethyl- | *Gaiella occulta* | 0.71774 | 0.0011779 |
| Hexadecane, 2,6,10,14-tetramethyl- | *Brevitalea* | 0.76054 | 0.00039355 |
| Hexadecane, 2,6,10,14-tetramethyl- | *Pedomicrobium* | 0.71335 | 0.0013038 |
| Hexadecane, 2,6,10,14-tetramethyl- | *Limnoglobus roseus* | 0.74309 | 0.00063099 |
| Hexadecane, 2,6,10,14-tetramethyl- | *Limisphaerangatamarikiensis* | 0.73078 | 0.00086154 |
| Hexadecane, 2,6,10,14-tetramethyl- | *Usitatibacter* | 0.74664 | 0.00057494 |
| Hexadecane, 2,6,10,14-tetramethyl- | *Reyranella soli* | 0.72992 | 0.00088011 |
| Hexanedioic acid, bis(2-ethylhexyl) ester | *Chthoniobacter flavus* | 0.7874 | 0.00017548 |
| Hexanedioic acid, bis(2-ethylhexyl) ester | *Tepidisphaera mucosa* | 0.74144 | 0.00065855 |
| Hexanedioic acid, bis(2-ethylhexyl) ester | *Gaiella occulta* | 0.71918 | 0.0011388 |
| Hexanedioic acid, bis(2-ethylhexyl) ester | *Brevitalea* | 0.71827 | 0.0011634 |
| Hexanedioic acid, bis(2-ethylhexyl) ester | *Limnoglobus roseus* | 0.72071 | 0.0010985 |
| Hexanedioic acid, bis(2-ethylhexyl) ester | *Zavarzinellaformosa* | 0.70727 | 0.0014964 |
| Hexanedioic acid, bis(2-ethylhexyl) ester | *Limisphaerangatamarikiensis* | 0.7108 | 0.0013817 |
| Hexanedioic acid, bis(2-ethylhexyl) ester | *Usitatibacter* | 0.83762 | 2.72E-05 |
| Hexanedioic acid, bis(2-ethylhexyl) ester | *Bradyrhizobium* | 0.70464 | 0.0015866 |
| Tetrachloroethylene | 2,4-Di-tert-butylphenol | 0.74733 | 0.00056454 |
| Tetrachloroethylene | Toluene | 0.99326 | 1.87E-15 |
| Tetrachloroethylene | *Chthoniobacter flavus* | 0.72946 | 0.00089002 |
| Tetrachloroethylene | *Gemmata* | 0.7034 | 0.0016304 |
| Tetrachloroethylene | *Paludibaculumfermentans* | 0.7821 | 0.00020764 |
| Tetrachloroethylene | *Gaiella occulta* | 0.71259 | 0.0013268 |
| Tetrachloroethylene | *Limnoglobus roseus* | 0.723 | 0.0010403 |
| Tetrachloroethylene | *Zavarzinellaformosa* | 0.72572 | 0.00097474 |
| Tetrachloroethylene | *Jatrophihabitans* | 0.73111 | 0.00085453 |
| Tetrachloroethylene | *Nocardioides* | 0.73069 | 0.0008635 |
| Tetrachloroethylene | *Paraconexibacteralgicola* | 0.77449 | 0.00026226 |
| Tetrachloroethylene | *Conexibacter* | 0.79584 | 0.00013297 |
| Tetrachloroethylene | *Reyranella* | 0.79294 | 0.00014649 |
| Toluene | *Paludibaculumfermentans* | 0.73429 | 0.00078977 |
| Toluene | *Paraconexibacteralgicola* | 0.73512 | 0.00077341 |
| Toluene | *Conexibacter* | 0.75958 | 0.00040428 |
| Toluene | *Reyranella* | 0.75022 | 0.00052257 |
| Toluene | *Usitatibacter* | 0.74224 | 0.00064496 |
| Toluene | *Rhabdothermincola* | 0.77696 | 0.00024329 |
| Toluene | *Bradyrhizobium* | 0.76806 | 0.00031729 |
| Eucalyptol | *Paludisphaera borealis* | 0.90067 | 8.24E-07 |
| Eucalyptol | *Candidatus Xiphinematobacter*sp. Idaho Grape | 0.94284 | 1.48E-08 |
| Eucalyptol | *Conexibacterwoesei* | 0.81931 | 5.73E-05 |
| Eucalyptol | *Mycobacterium* | 0.87114 | 5.31E-06 |
| Eucalyptol | *Actinomaduraamylolytica* | 0.87356 | 4.64E-06 |
| Eucalyptol | *Acidibrevibacteriumfodinaquatile* | 0.85553 | 1.20E-05 |
| gamma-Gurjunene | *Paludisphaera borealis* | 0.89579 | 1.16E-06 |
| gamma-Gurjunene | *Candidatus Xiphinematobacter*sp. Idaho Grape | 0.97335 | 5.29E-11 |
| gamma-Gurjunene | *Conexibacterwoesei* | 0.90497 | 5.99E-07 |
| gamma-Gurjunene | *Mycobacterium* | 0.92393 | 1.19E-07 |
| gamma-Gurjunene | *Actinomaduraamylolytica* | 0.94834 | 7.05E-09 |
| gamma-Gurjunene | *Acidibrevibacteriumfodinaquatile* | 0.80954 | 8.24E-05 |
| (-)-Globulol | *Paludisphaera borealis* | 0.84371 | 2.08E-05 |
| (-)-Globulol | *Candidatus Xiphinematobacter*sp. Idaho Grape | 0.96901 | 1.62E-10 |
| (-)-Globulol | *Conexibacterwoesei* | 0.77815 | 0.00023463 |
| (-)-Globulol | *Mycobacterium* | 0.81223 | 7.47E-05 |
| (-)-Globulol | *Actinomaduraamylolytica* | 0.83903 | 2.56E-05 |
| (-)-Globulol | *Acidibrevibacteriumfodinaquatile* | 0.80011 | 0.00011501 |
| (-)-Globulol | *Phenylobacterium*sp. HYN0004 | 0.70303 | 0.0016438 |
| Epiglobulol | *Paludisphaera borealis* | 0.83682 | 2.81E-05 |
| Epiglobulol | *Candidatus Xiphinematobacter*sp. Idaho Grape | 0.95542 | 2.38E-09 |
| Epiglobulol | *Conexibacterwoesei* | 0.75421 | 0.00046909 |
| Epiglobulol | *Mycobacterium* | 0.78815 | 0.00017131 |
| Epiglobulol | *Actinomaduraamylolytica* | 0.81319 | 7.21E-05 |
| Epiglobulol | *Acidibrevibacteriumfodinaquatile* | 0.79407 | 0.00014109 |
| Epiglobulol | *Phenylobacterium*sp. HYN0004 | 0.70472 | 0.0015837 |
| Ferruginol | *Paludisphaera* | 0.8475 | 0.007883 |
| Ferruginol | *Candidatus Xiphinematobacter* | 0.84327 | 0.0085286 |
| Ferruginol | *Acidobacterium* | 0.96178 | 0.00013558 |
| Ferruginol | *Acidibrevibacterium* | 0.81619 | 0.013465 |
| Bicyclo[2.2.1]heptan-2-ol, 1,7,7-trimethyl-, (1S-endo)- | *Paludisphaera* | 0.85283 | 0.0071146 |
| Bicyclo[2.2.1]heptan-2-ol, 1,7,7-trimethyl-, (1S-endo)- | *Candidatus Xiphinematobacter* | 0.98763 | 4.69E-06 |
| Bicyclo[2.2.1]heptan-2-ol, 1,7,7-trimethyl-, (1S-endo)- | *Acidobacterium* | 0.90558 | 0.0019584 |
| Bicyclo[2.2.1]heptan-2-ol, 1,7,7-trimethyl-, (1S-endo)- | *Actinomaduraamylolytica* | 0.84469 | 0.0083082 |
| Bicyclo[2.2.1]heptan-2-ol, 1,7,7-trimethyl-, (1S-endo)- | *Acidibrevibacterium* | 0.76628 | 0.026583 |

Supplementary table 8: Pearson’s correlation coefficients and respective p-values of correlation analysis between soil DHA and metabolites/bacteria/compounds/elements

|  | **Metabolite/Bacteria/compound/element** | **Pearson correlation value (r)** | **p-value** |
| --- | --- | --- | --- |
| Soil DHA | Tetrachloroethylene | 0.78 | 0.00017 |
| Soil DHA | Toluene | 0.79 | 0.00016 |
| Soil DHA | Squalene | 0.75 | 0.0005 |
| Soil DHA | benzoxazole, 2-(chloromethyl)- | 0.87 | 4.82E-06 |
| Soil DHA | Silane, diethyl heptyloxy-octadecyloxy- | 0.76 | 0.0003 |
| Soil DHA | Sodium | -0.76 | 0.0004 |
| Soil DHA | Iron | -0.71 | 0.0015 |
| Soil DHA | Oxirane, 2-(1,1-dimethylethyl)-3-ethyl-, cis- | -0.8 | 4.52E-06 |
| Soil DHA | *Candidatus Solibacterusitatus* | -0.78 | 0.0002 |
| Soil DHA | *Gemmatimonas phototrophica* | -0.75 | 0.0005 |
| Soil DHA | *Sorangiumcellulosum* | -0.74 | 0.0006 |
| Soil DHA | *Rhodoplanes* sp. Z2-YC6860 | -0.76 | 0.0004 |
| Soil DHA | *Kribbellaflavida* | -0.73 | 0.0009 |

**Supplementary table 9.** Toxic chemicals found exclusively in TM3 samples

| **Compound Name** | **Component RT** | **CAS#** | **Formula** | **Component Area** | **Match Factor** | **Pollutant type** |
| --- | --- | --- | --- | --- | --- | --- |
| Benzene, propoxy- | 3.6143 | 622-85-5 | C_9_H_12_O | 727985.3 | 71.4 | Benzene compound |
| Ethane, 1,2-dibromo- | 31.6546 | 106-93-4 | C_2_H_4_Br_2_ | 451590.1 | 51.2 | Brominated compound |
| Benzenepropanamide, N-[7- dioxo-2-oxa-6,9-diazabicyclo[10.2.2]hexadeca- [3R-[3R*,4R*(S*),7R*(S*]]- | 32.9361 | 53797-29-8 | C_36_H_42_N_4_O_6_ | 177432.7 | 51.4 | Benzene compound |
| 2,4-Pentanedione, ion (1-), lithium | 4.6803 | 18115-70-3 | C_5_H_7_LiO_2_ | 167871 | 69.9 | Lithium compound |
| Silane, diethyldecyloxypentyloxy- | 36.6944 | 1000363-03-7 | C_19_H_42_O_2_Si | 160483.6 | 56 | Silane compound |
| benzoic acid, 4-(2-bromoethyl)-, 4-formylphenyl | 37.3769 | 1000398-06-5 | C_16_H_13_BrO_3_ | 156662 | 68.5 | Brominated compound |
| l-Norvaline, N-(2-chloroethoxycarbonyl)-, heptyl | 29.4343 | 1000328-72-6 | C_15_H_28_ClNO_4_ | 142384.3 | 57.9 | Chlorinated compound |
| 1,3-Benzenediol, o-octanoyl- | 30.3341 | 1000330-67-8 | C_14_H_20_O_3_ | 111248.7 | 52.9 | Benzene compound |
| 6-Bromohexanoic acid, 3-methylphenyl ester | 12.7932 | 1000307-61-5 | C_13_H_17_BrO_2_ | 108351.6 | 63 | Brominated compound |
| Thiophene-2-acetic acid, 2,2,3,3,4,4,4- | 32.9215 | 1000330-95-9 | C_10_H_7_F_7_O_2_S | 103575.8 | 51 | Fluorinated compound |
| 3-Fluorobenzoic acid, 2-tetrahydrofurylmethyl ester | 24.1161 | 1000279-04-4 | C_12_H_13_FO_3_ | 73886.3 | 61.6 | Fluorinated compound |

**Supplementary table 10**. The table depicting the dispersion phi, slope, deviance, G-value and p-values of the GLM analyses of soil parameters and bacterial diversity.

| Sl. No. | Parameter 1 | Parameter 2 | Dispersion phi | Slope | Deviance | G-value | p-value(slope=0) |
| --- | --- | --- | --- | --- | --- | --- | --- |
| 1 | N | P | 19037 (estimated) | 0.48617  (Std. err.: 0.58961) | 2.8555E05 | 0.67989 | 0.40962 |
| 2 | AN | NN | 90.409 (estimated) | -0.017594  (Std. err.: 0.018395) | 1356.1 | 0.91477 | 0.33885 |
| 3 | AP | TOC | 4.9507 (estimated) | 0.00011422  (Std. err. : 0.00023184) | 74.26 | 0.24271 | 0.62226 |
| 4 | Na | K | 16435 (estimated) | -0.28274  (Std. err.: 0.38854) | 2.4652E05 | 0.52955 | 0.4668 |
| 5 | Mg | Ca | 67234 (estimated) | 0.48796  (Std. err: 0.18322) | 1.0085E06 | 7.0932 | 0.0077378 |
| 6 | Pb | Ni | 0.05535 (estimated) | 0.069814  (Std. err.: 0.03157) | 0.83025 | 4.8901 | 0.027011 |
| 7 | Zn | Cu | 212.98 (estimated) | (-0.19371  Std. err.:  1.4887) | 3194.7 | 0.016931 | 0.89647 |
| 8 | Al | Fe | 11785 (estimated) | (0.00055214 Std. err.: 0.00035642) | 1.7678E05 | 2.3997 | 0.12136 |
| 9 | Cd | Cr | 0.016813 (estimated) | (0.21415 Std. err.: 0.077814) | 0.25219 | 7.5736 | 0.0059231 |
| 10 | Mn | Al | 28480 (estimated) | 0.0012409 (Std. err.: 0.00055408) | 4.272E05 | 5.0157 | 0.025119 |
| 11 | Shannon diversity index | Soil pH | 0.55203 (estimated) | 0.10019  (Std. err.: 0.21003) | 8.2804 | 0.22757 | 0.63333 |
| 12 | Shannon diversity index | Soil DHA | 0.54146 (estimated) | -0.70398  (Std. err.: 0.97171) | 8.1219 | 0.52486 | 0.46878 |
| 13 | Shannon diversity index | N | 0.50955 (estimated) | -0.0037323  (Std. err.: 0.0030504) | 7.6433 | 1.497 | 0.22114 |
| 14 | Shannon diversity index | P | 0.55572 (estimated) | 0.00048509 (Std. err.: 0.0013645) | 8.3358 | 0.12639 | 0.7222 |
| 15 | Shannon diversity index | TOC | 0.44864 (estimated) | -0.14905  (Std. err.: 0.077106) | 6.7296 | 3.7368 | 0.053226 |
| 16 | Shannon diversity index | NN | 0.55988 (estimated) | 0.0023466  (Std. err.: 0.019726) | 8.3981 | 0.014151 | 0.90531 |
| 17 | Shannon diversity index | AN | 0.55744 (estimated) | -0.00040825  (Std. err.: 0.0014444) | 8.3615 | 0.079886 | 0.77745 |
| 18 | Shannon diversity index | AP | 0.55553 (estimated) | 2.8167E-05 (Std. err.: 7.7663E-05) | 8.333 | 0.13154 | 0.71684 |
| 19 | Shannon diversity index | K | 0.53724 (estimated) | -0.0017864 (Std. err.: 0.0022215) | 8.0586 | 0.64669 | 0.4213 |
| 20 | Shannon diversity index | Na | 0.56039 (estimated) | -3.338E-05  (Std. err.: 0.0014818) | 8.4058 | 0.00050746 | 0.98203 |
| 21 | Shannon diversity index | Ca | 0.53525 (estimated) | -0.00043407 (Std. err.: 0.00051695) | 8.0287 | 0.70507 | 0.40108 |
| 22 | Shannon diversity index | Mg | 0.54184 (estimated) | 0.00043293 (Std. err.: 0.00060397) | 8.1277 | 0.51382 | 0.47349 |
| 23 | Shannon diversity index | Pb | 0.55985 (estimated) | -0.012183  (Std. err.: 0.10041) | 8.3978 | 0.014723 | 0.90342 |
| 24 | Shannon diversity index | Ni | 0.47461 (estimated) | -1.0812  (Std. err.: 0.65659) | 7.1192 | 2.7114 | 0.099631 |
| 25 | Shannon diversity index | Cd | 0.34314 (estimated) | -2.9303  (Std. err.: 0.95086) | 5.1472 | 9.4972 | 0.0020579 |
| 26 | Shannon diversity index | Cu | 0.33381 (estimated) | -0.18807  (Std. err.: 0.058937) | 5.0071 | 10.182 | 0.0014179 |
| 27 | Shannon diversity index | Cr | 0.53935 (estimated) | -0.33722  (Std. err.: 0.44073) | 8.0903 | 0.58542 | 0.4442 |
| 28 | Shannon diversity index | Zn | 0.42638 (estimated) | -0.025071  (Std. err.: 0.011546) | 6.3957 | 4.715 | 0.0299 |
| 29 | Shannon diversity index | Fe | 0.54542 (estimated) | -0.0010471 (Std. err.: 0.0016309) | 8.1813 | 0.41218 | 0.52086 |
| 30 | Shannon diversity index | Mn | 0.55873 (estimated) | -0.00020972 (Std. err.: 0.00099003) | 8.381 | 0.044871 | 0.83224 |
| 31 | Shannon diversity index | Al | 0.53245 (estimated) | -2.126E-06 (Std. err.: 2.3957E-06) | 7.9868 | 0.78747 | 0.37487 |

**Supplementary figures**

**
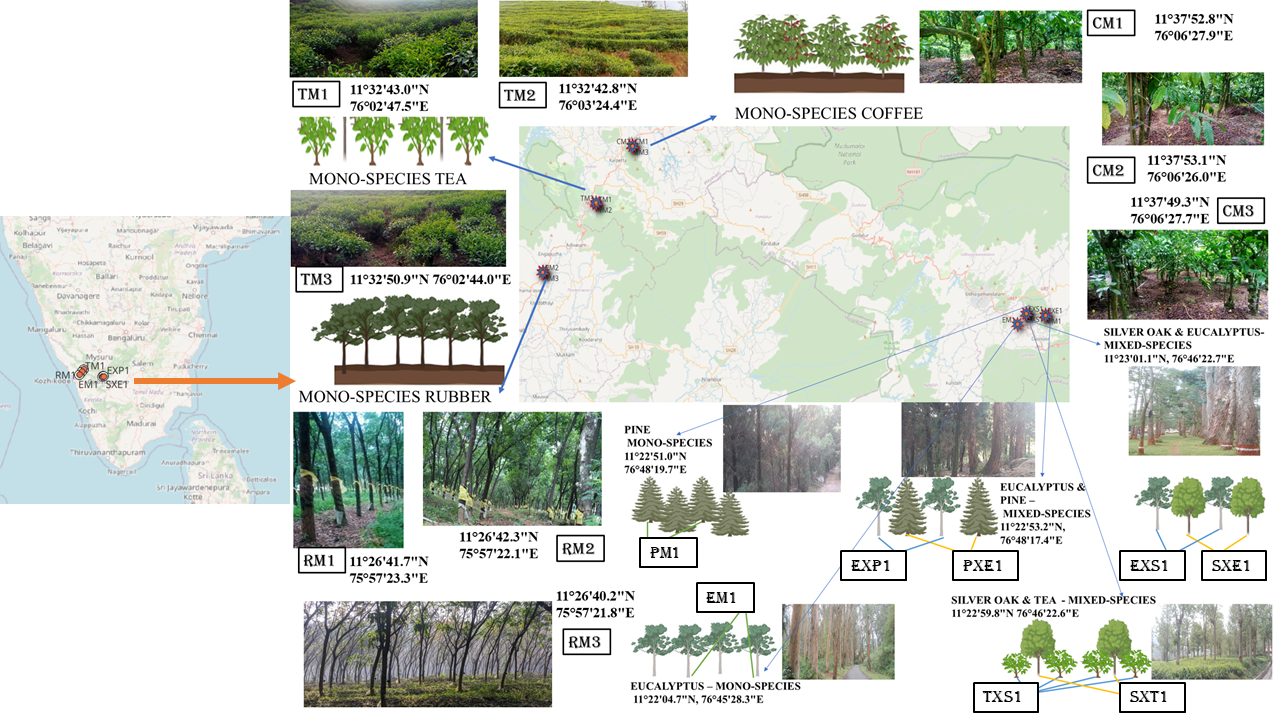
**

**Supplementary Figure 1**. Sample collection sites across the Western Ghats regions of Wayanad (Kerala) and Coonoor (Tamil Nadu)

**
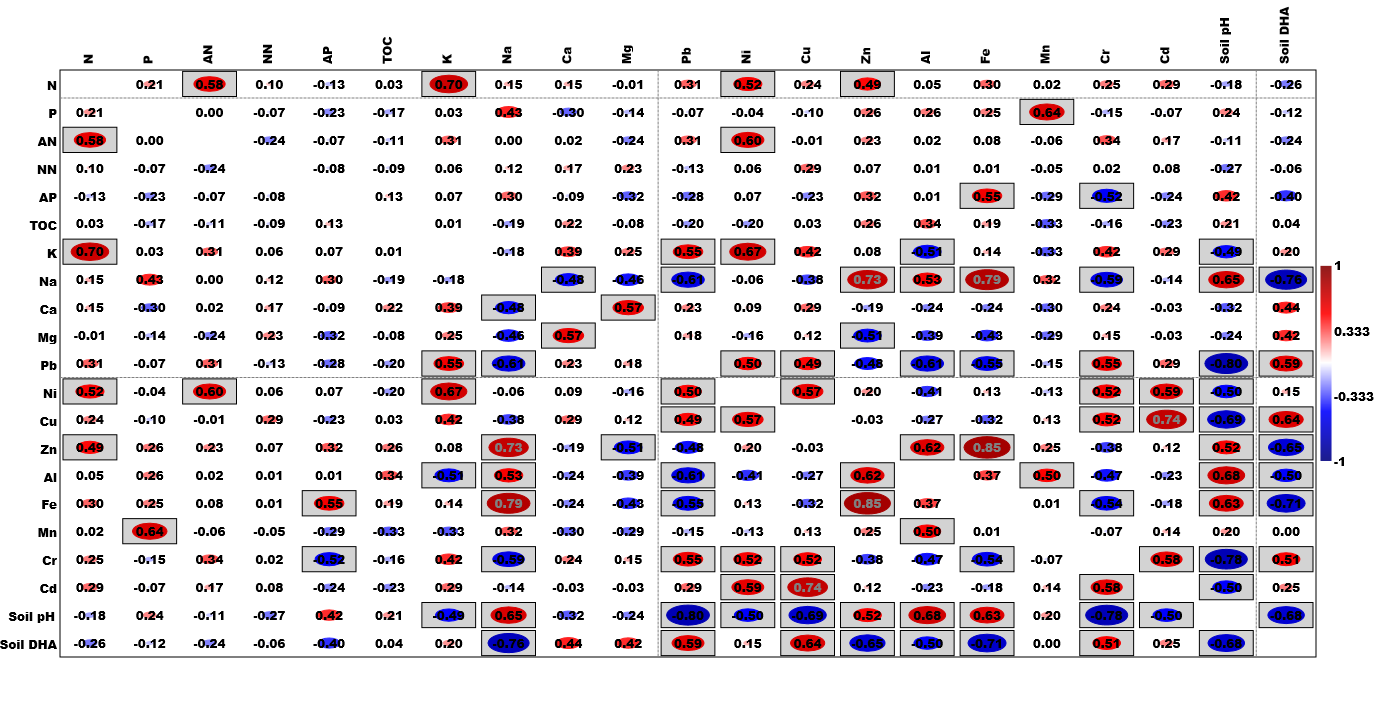
**

**Supplementary Figure 2**. Pearson correlation plot of physicochemical parameters, heavy metal concentrations and soil DHA, generated using PAST 4.17 software. All the significant correlations (p-value < 0.05) are highlighted with square boxes.


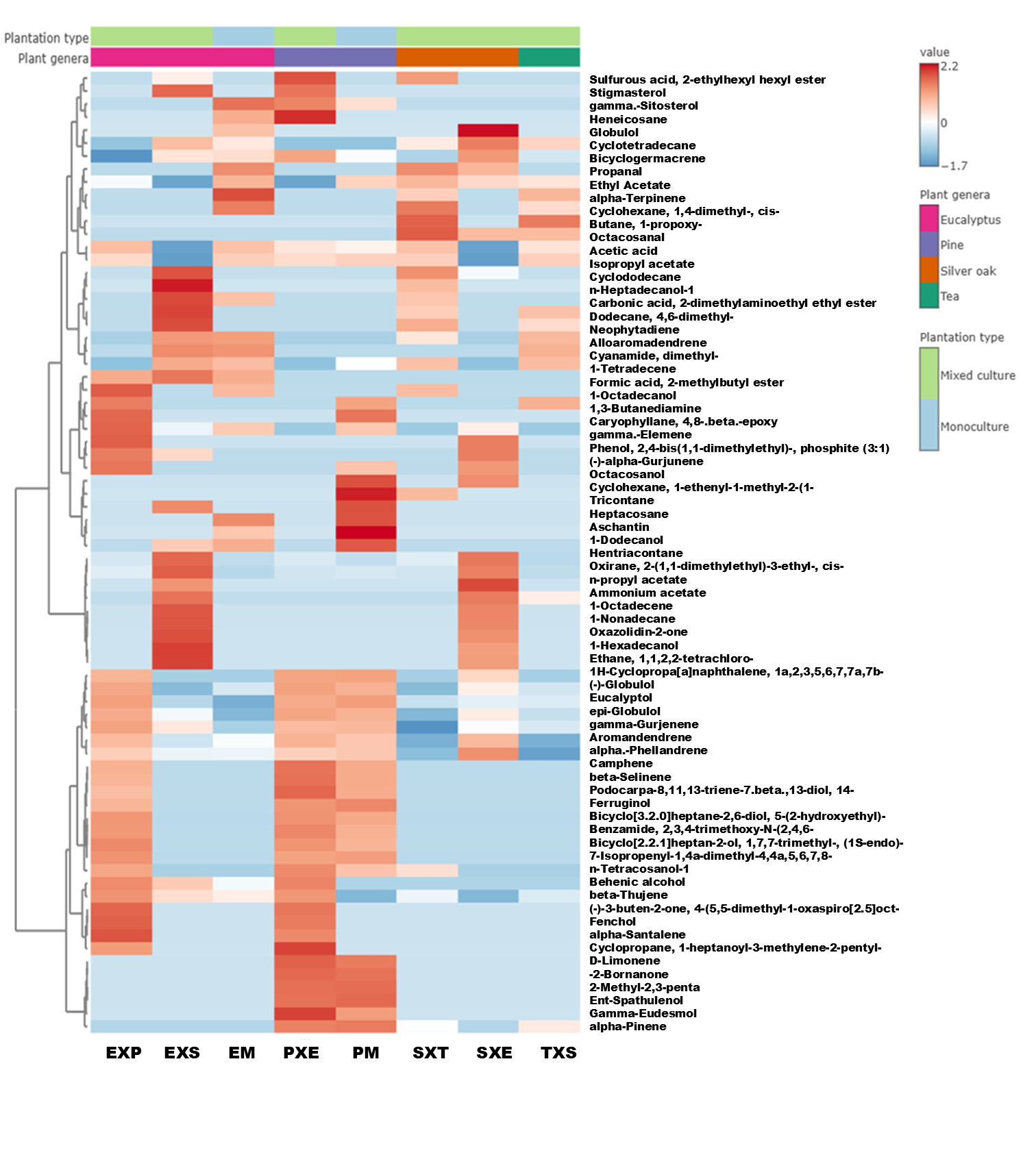


**Supplementary Figure3.** Heatmap of the 74 metabolites detected across the rhizosphere samples from Coonoor, generated using MetaboAnalyst 6.0 and using Euclidean distance measure and Ward’s clustering Algorithm. The scale shows the Z-score measures; the red-coloured boxes represent higher values (above the average), and blue represents lower values (below the average).

**
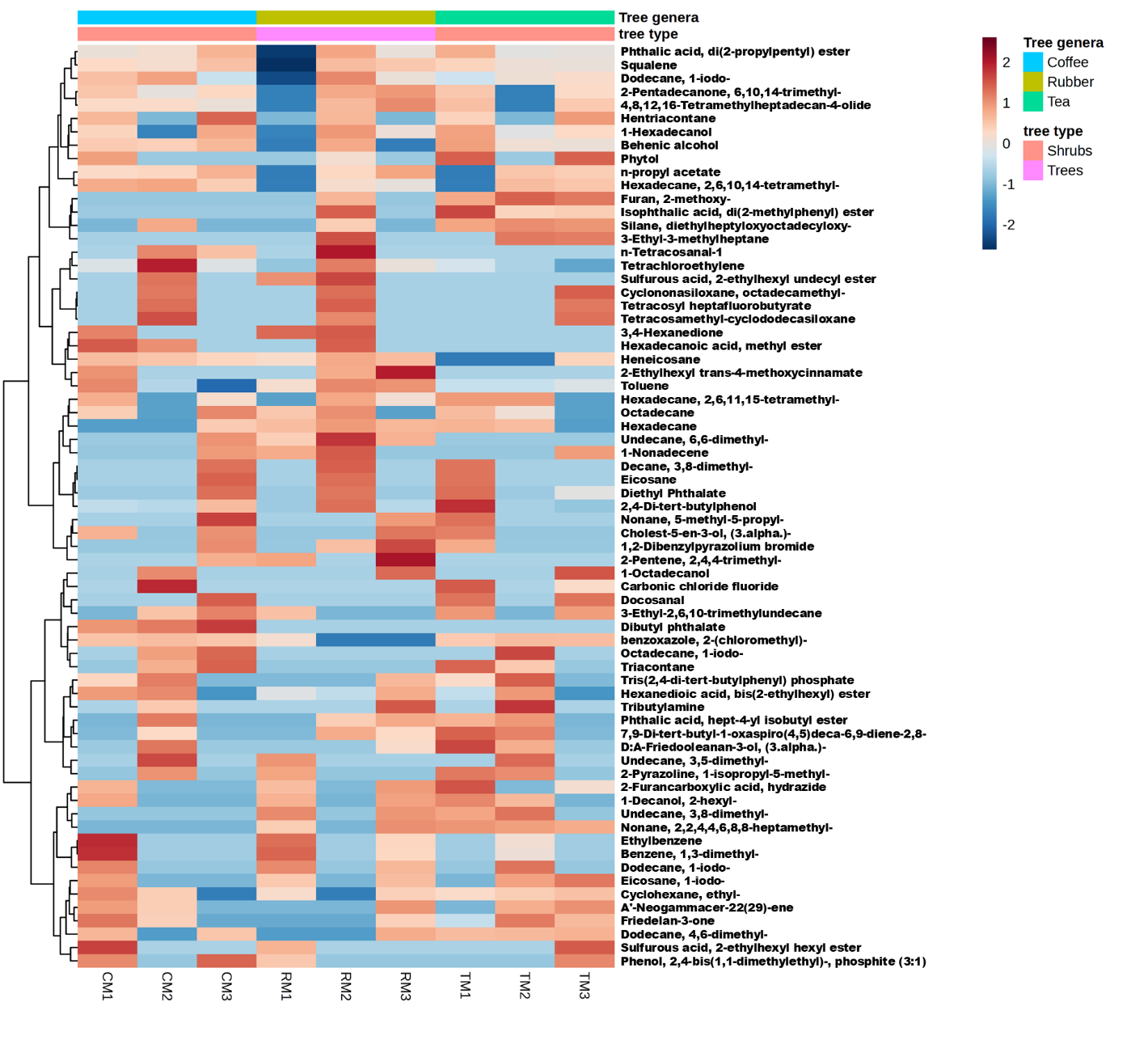
**

**Supplementary Figure 4.** Heatmap of the metabolites detected across the rhizosphere samples from Kerala, generated using MetaboAnalyst 6.0 and using Euclidean distance measure and Ward’s clustering Algorithm. The scale shows the Z-score measures; the red-coloured boxes represent higher values (above the average), and blue represents lower values (below the average).

**
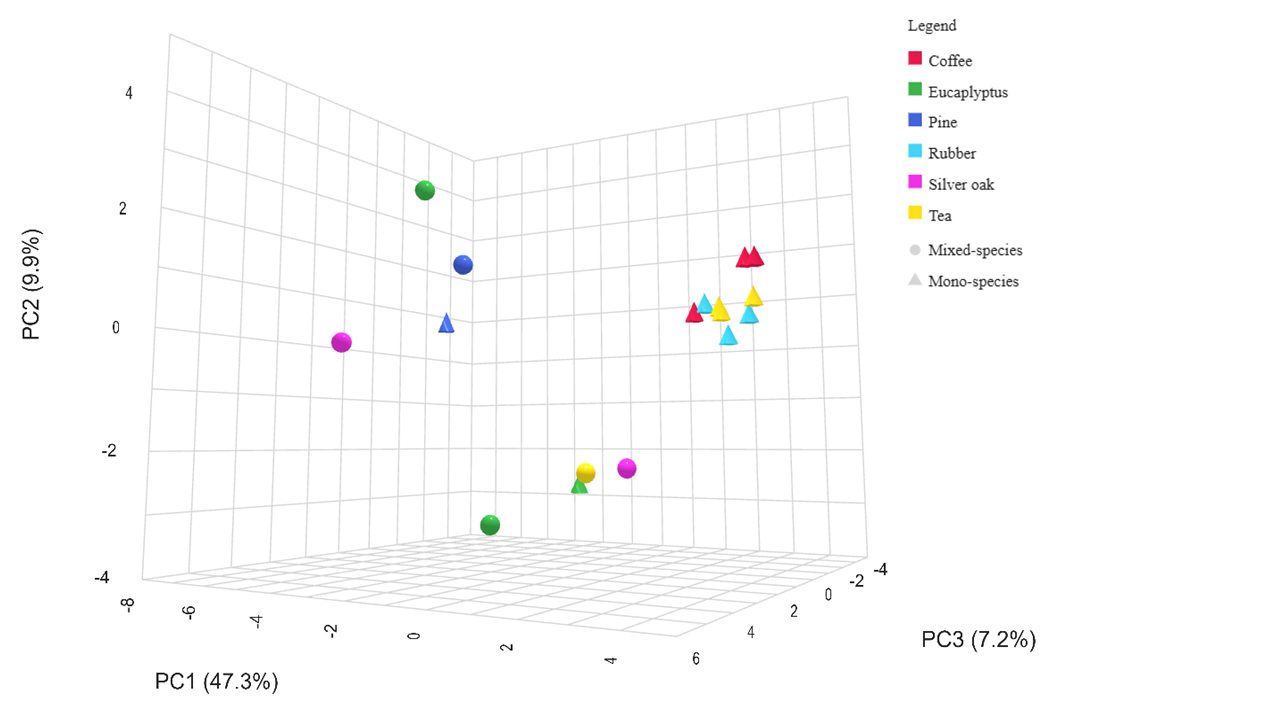
**

**
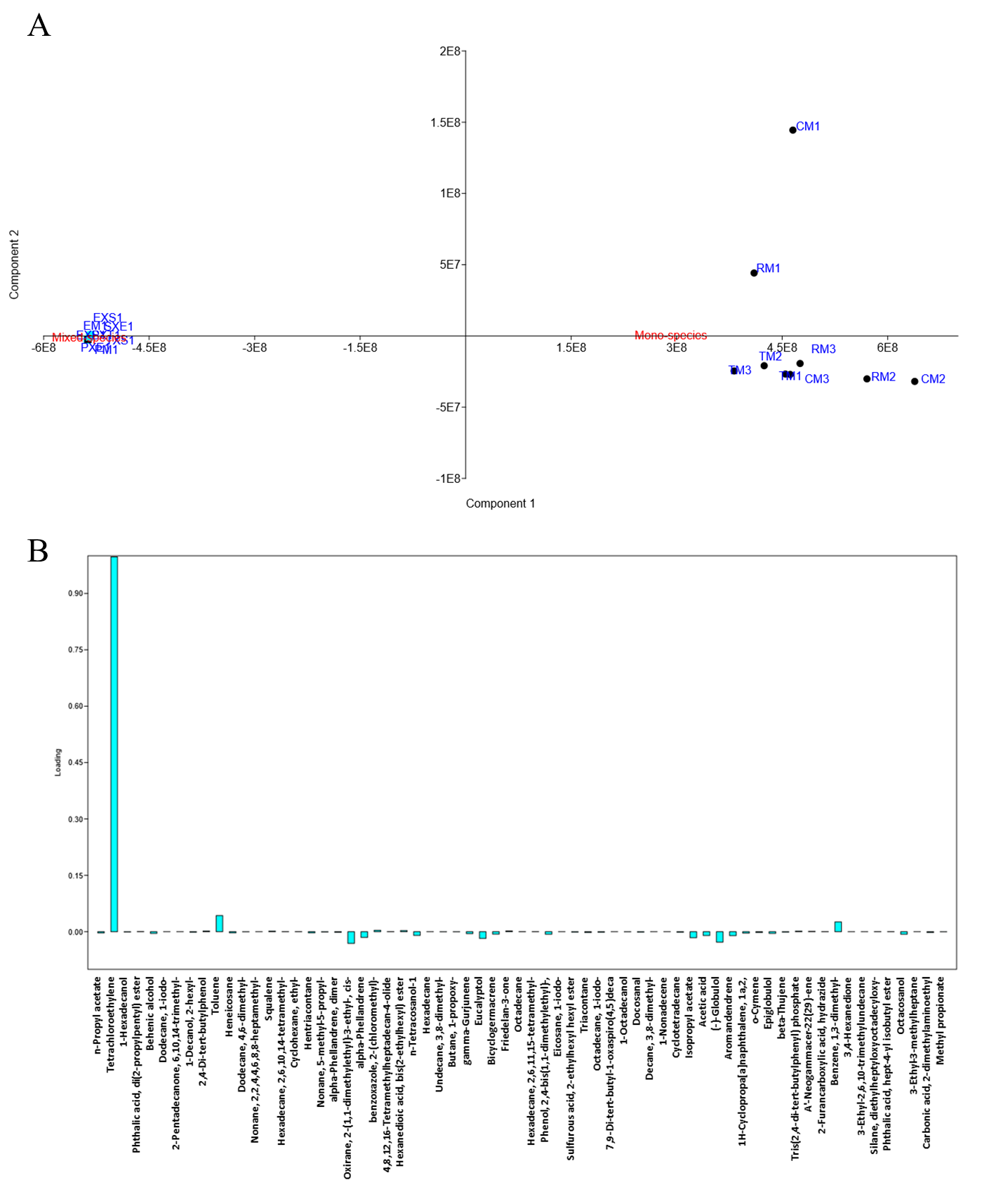
**

**Supplementary Figure 5.** Principal Component Analysis (PCA) of plant metabolites across the 17 rhizosphere samples (generated using MetaboAnalyst 6.0), depicting the top 3 principal components explaining the variance in the dataset irrespective of the class labels (plant/plantation types) provided. Principal Component Analysis (PCA) using PAST v4.17 software. (A) PCA plot of the rhizosphere samples based on the metabolome profiles. (B) The PCA loading plot showing the key metabolites driving separation.

**
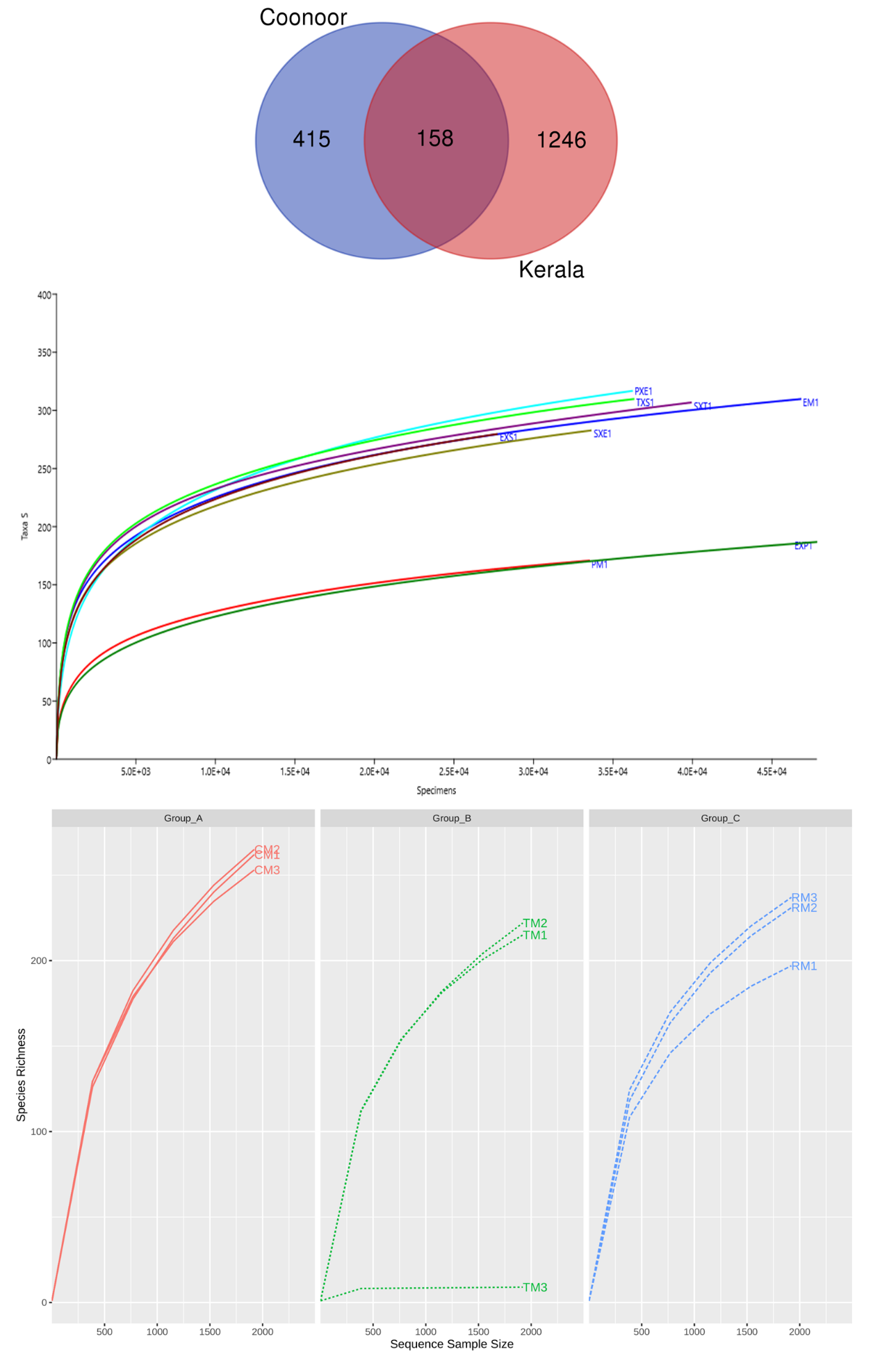
**

**Supplementary Figure6**. Venn diagram showing the OTUs detected and the rarefaction curves showing the sequencing depth. (A) Venn diagram (B) Rarefaction curve of Coonoor samples (C) Rarefaction curve of Coonoor samples

**
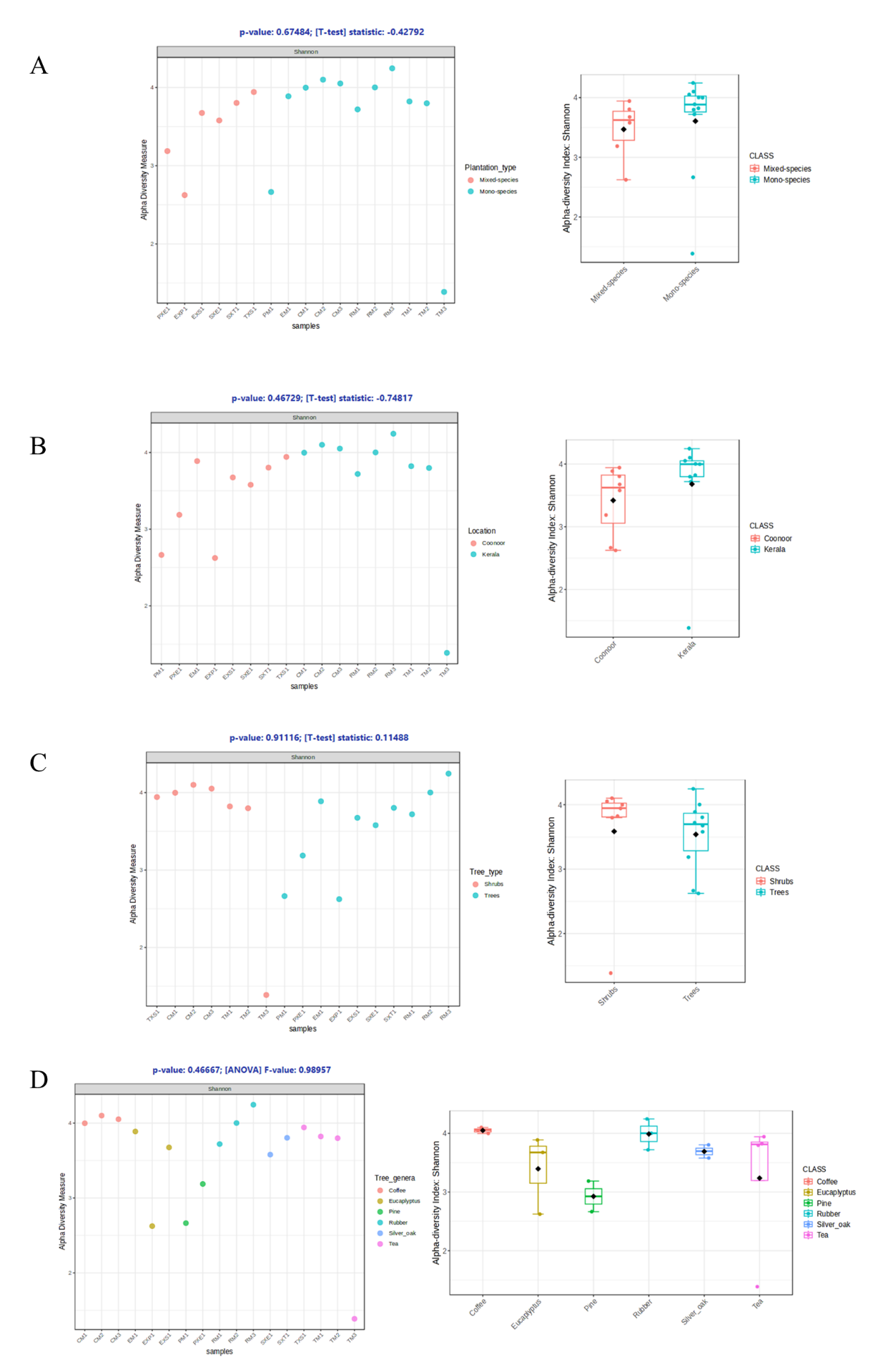
**

**Supplementary Figure 7**. Alpha diversity (Shannon) plots at the species level, grouped based on (A) plantation types, (B) sampling location, (C) plant types and (D) plant genera, calculated using Welch T-test/ANOVA, constructed using MicrobiomeAnalyst 2.0.

**
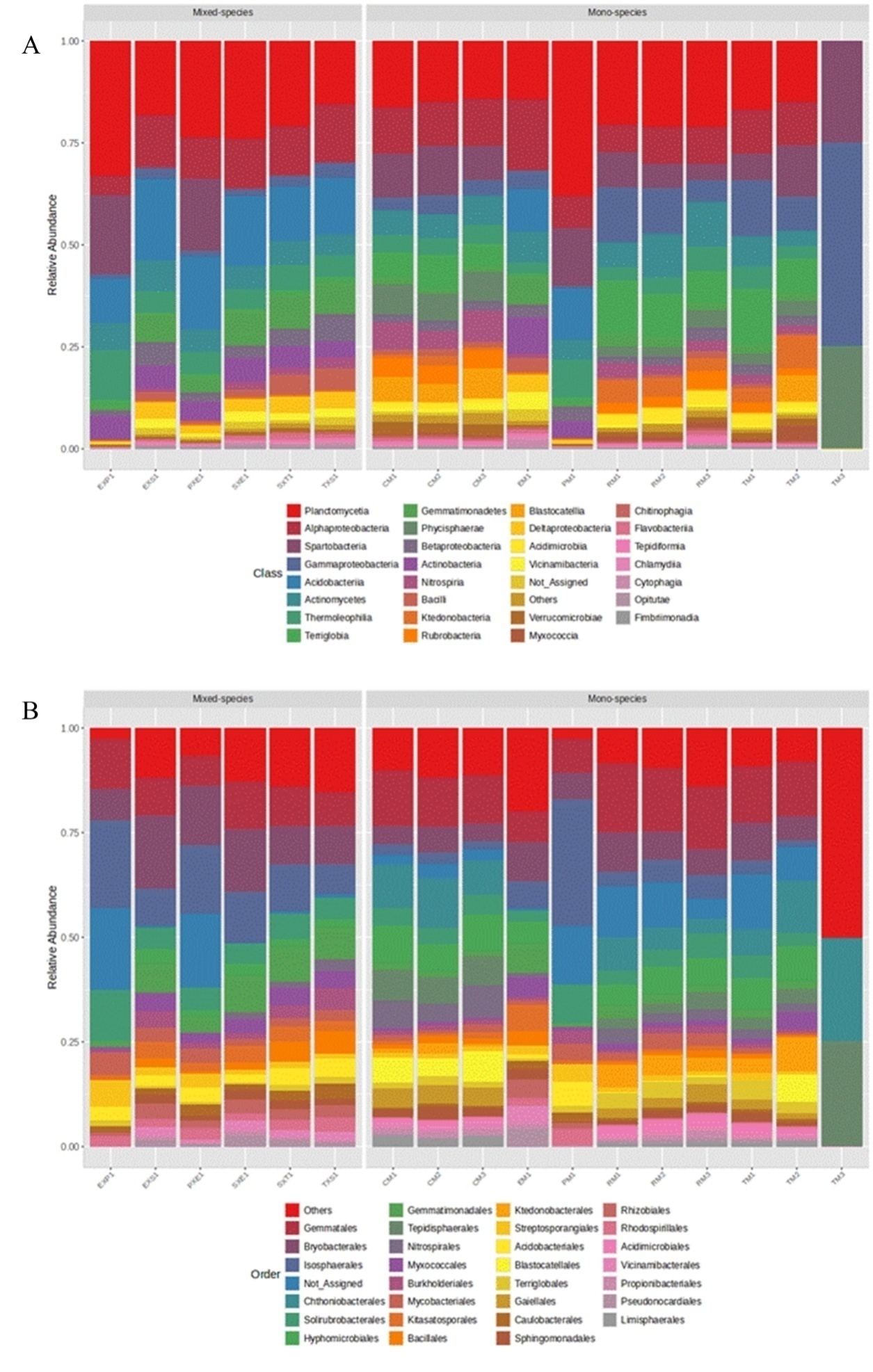
Supplementary Figure 8**. Stacked bar plots depicting the (A) class-level and (B) order-level relative abundances of various bacterial taxa across the samples, constructed using MicrobiomeAnalyst 2.0.

**
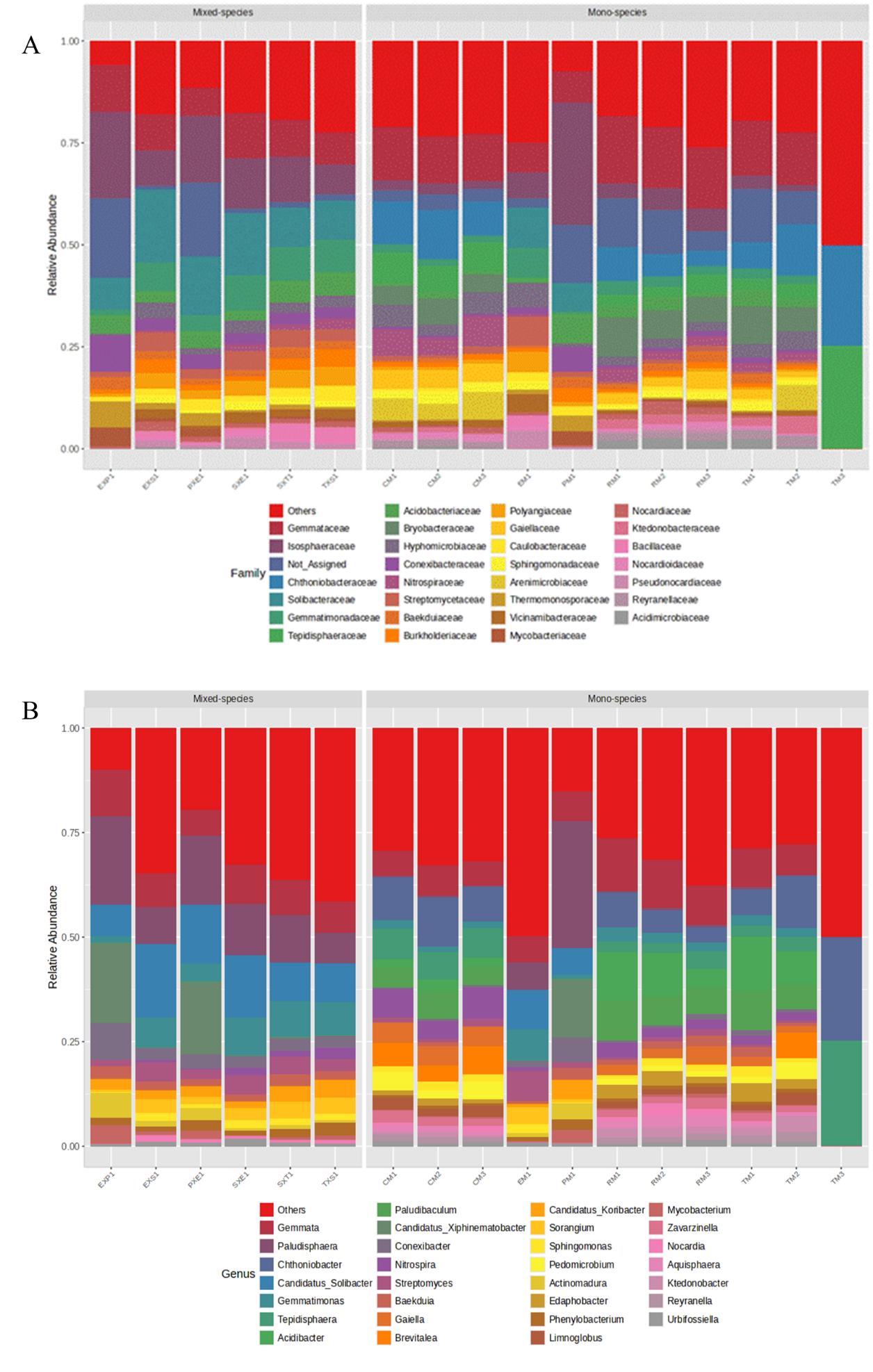
Supplementary Figure 9**. Stacked bar plots depicting the (A) family-level and (B) genus-level relative abundances of various bacterial taxa across the samples, constructed using MicrobiomeAnalyst 2.0.


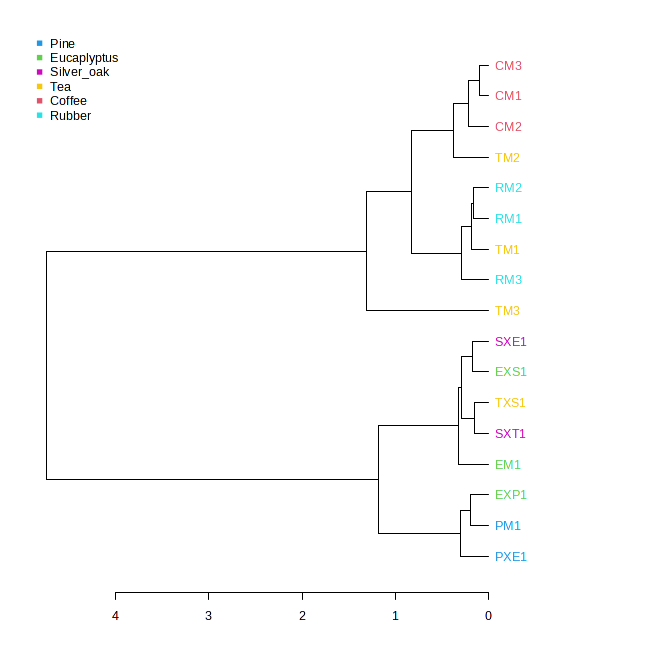
**Supplementary Figure 10.** Cluster dendrograms depicting the variations between the samples at the species level, constructed using MicrobiomeAnalyst 2.0 (Distance measure- Bray-Curtis Index, Clustering algorithm– Ward and experimental factor- Plant genera). The colour code is given based on the plant genera.

**
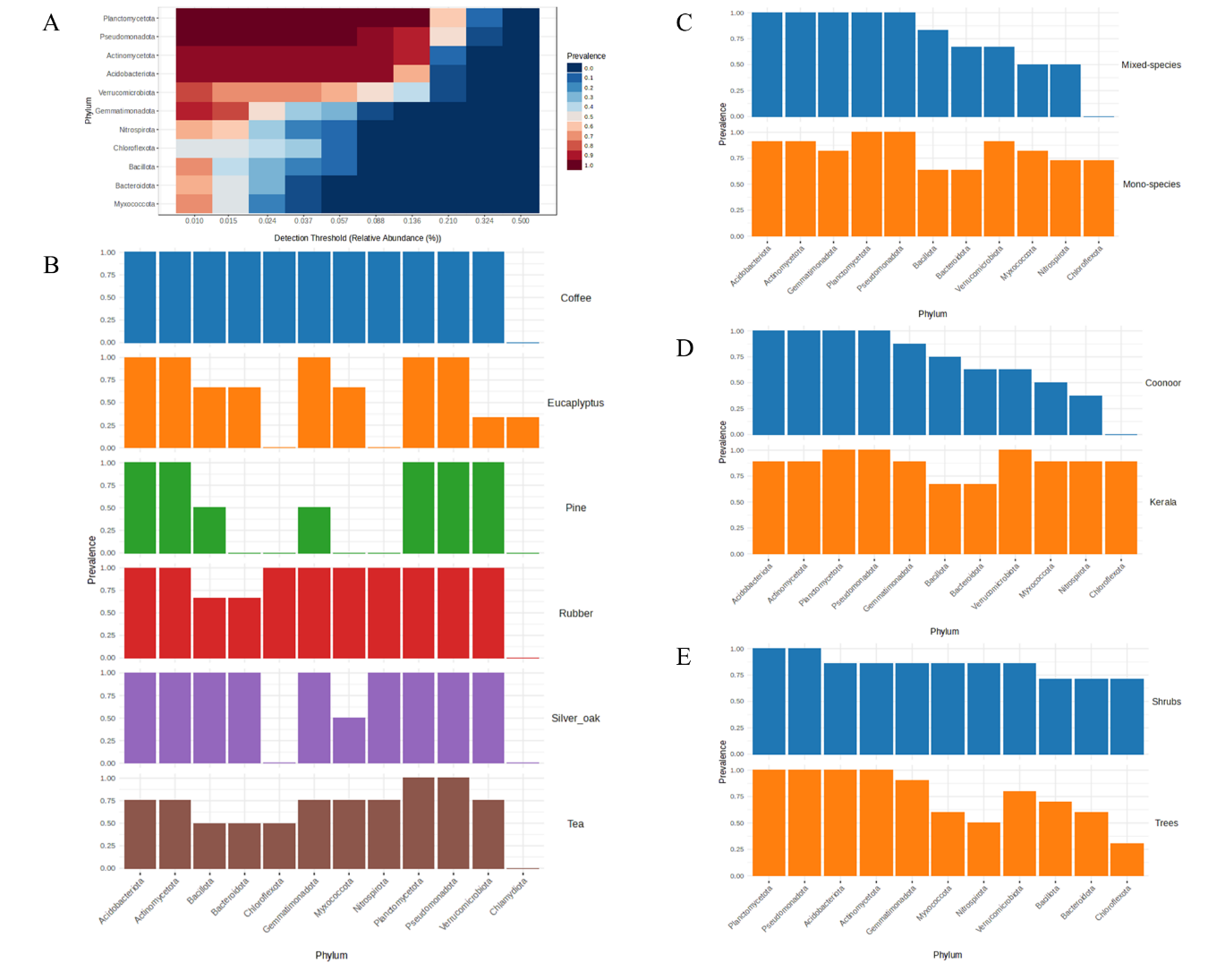
**

**Supplementary Figure11.** The phylum-level core bacteriome of the rhizophere soil samples, obtained by applying the parameters of sample prevalence percentage (≥20%) and relative abundance (≥0.01%) (constructed using MicrobiomeAnalyst 2.0). (A) Core bacteriome heatmap. Bar plots depicting core bacteriome variations across the sample groups based on (B) plant genera, (C) plantation types, (D) sampling location, and (E) plant types.


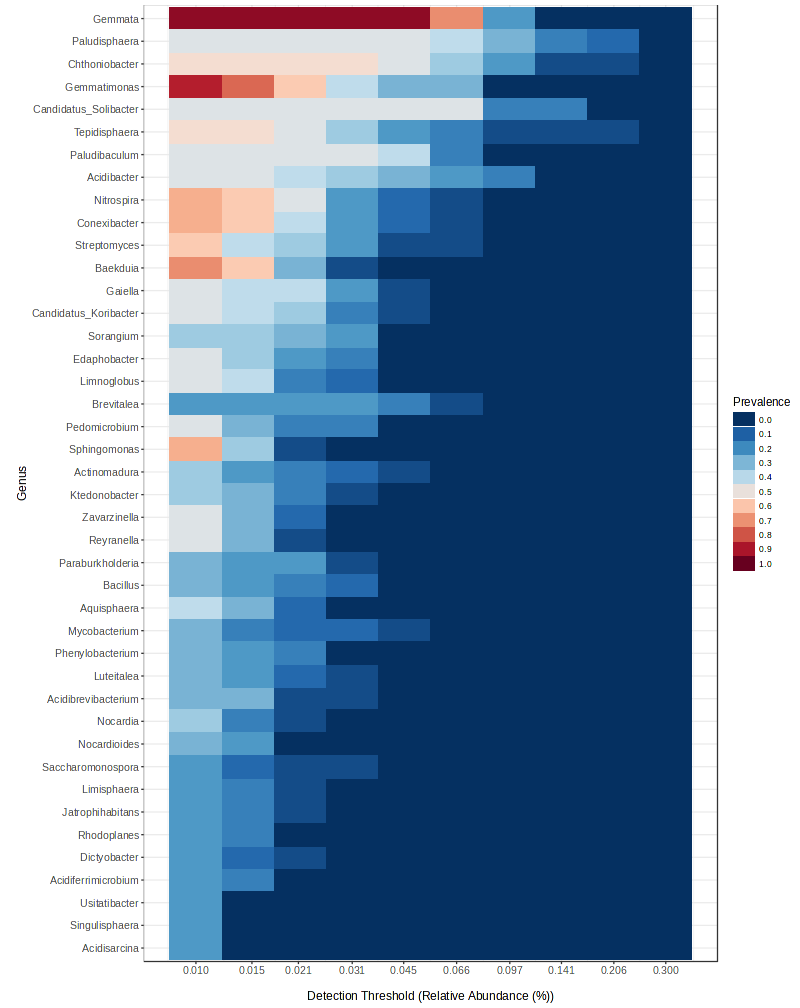


**Supplementary Figure 12.** Core bacteriome heatmap at the genus-level was obtained by applying the parameters of sample prevalence percentage (≥20%) and relative abundance (≥0.01%) (constructed using MicrobiomeAnalyst 2.0).

**
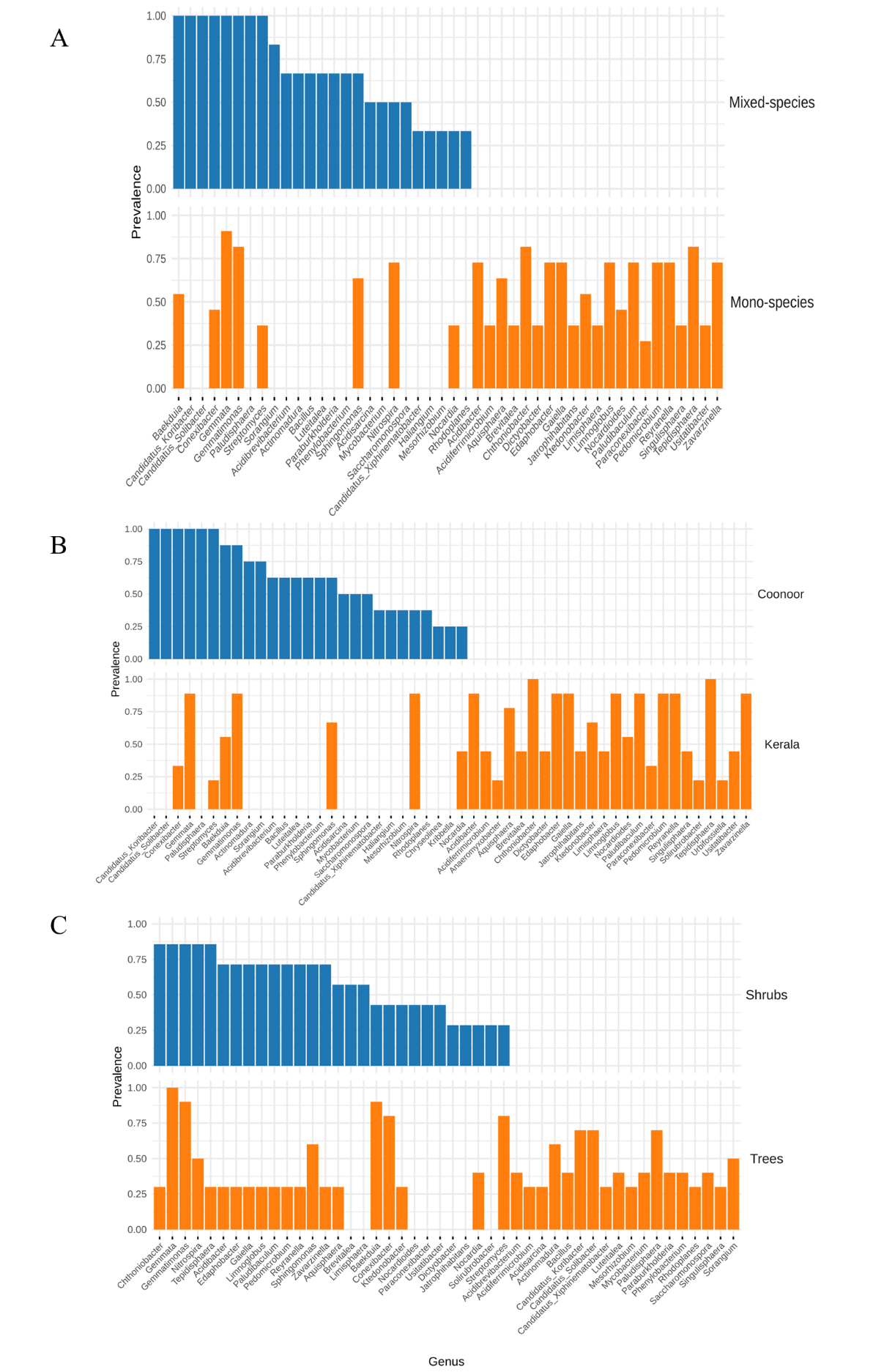
Supplementary Figure 13.** Bar plots depicting the genus-level core bacteriome variations across the sample groups based on (A) plantation types, (B) sampling location, and (C) plant types;obtained by applying the parameters of sample prevalence percentage (≥20%) and relative abundance (≥0.01%) (constructed using MicrobiomeAnalyst 2.0).

**
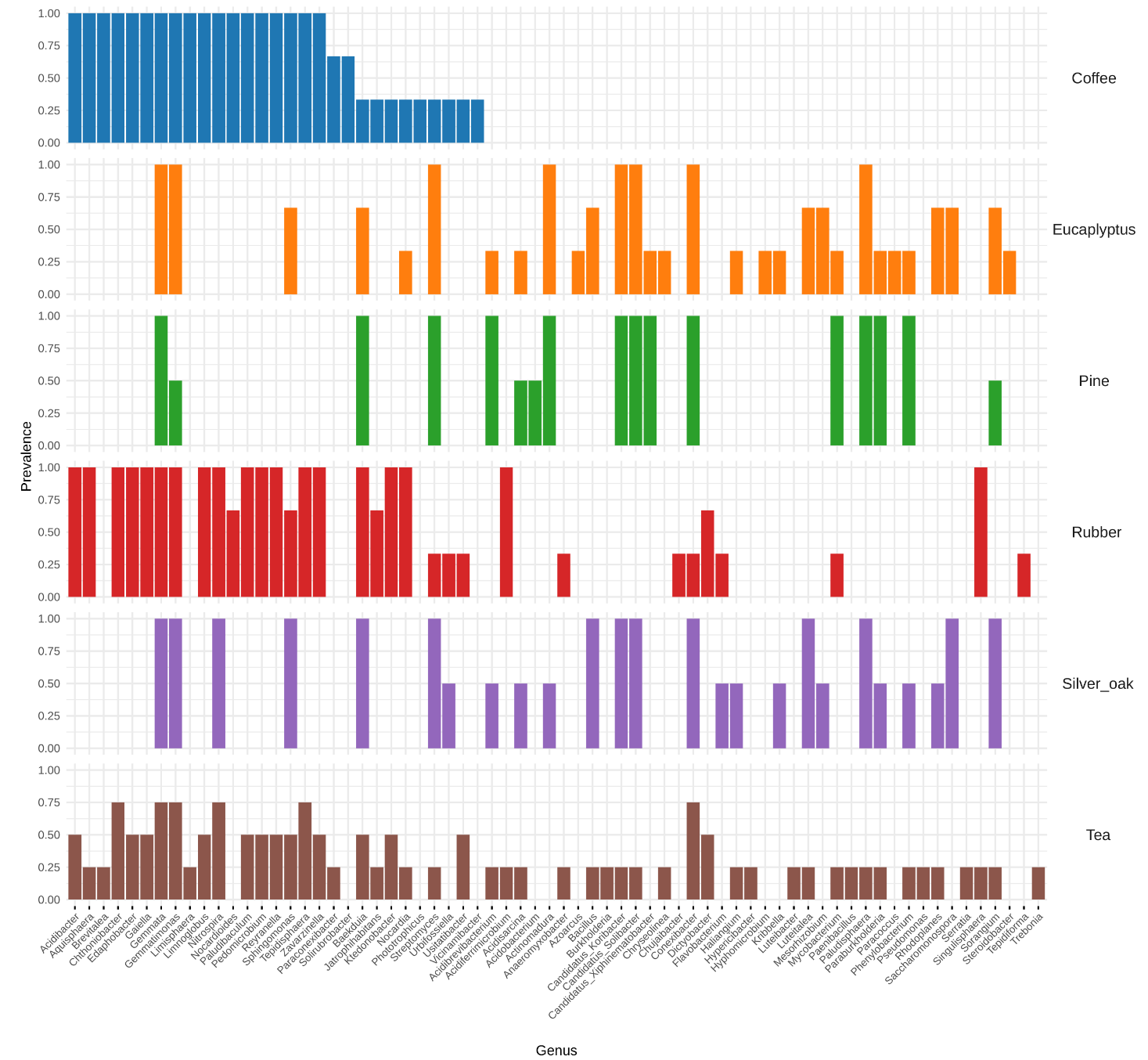
Supplementary Figure 14.** Bar plots depicting the genus-level core bacteriome variations across the sample groups based on ‘plant genera’;obtained by applying the parameters of sample prevalence percentage (≥20%) and relative abundance (≥0.01%) (constructed using MicrobiomeAnalyst 2.0).

**
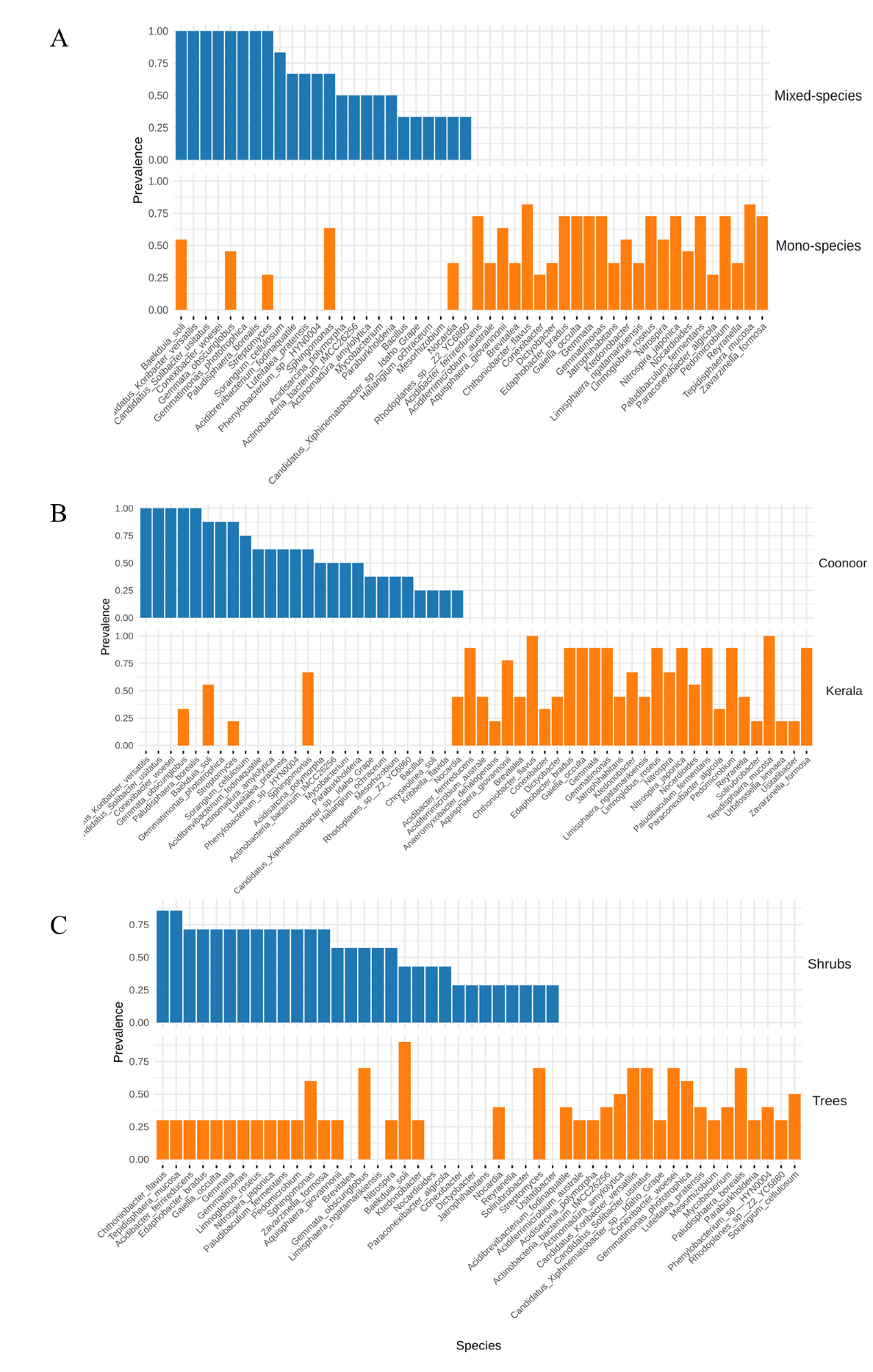
****Supplementary Figure 15.** Bar plots depicting the species-level core bacteriome variations across the sample groups based on (A) plantation types, (B) sampling location, and (C) plant types;obtained by applying the parameters of sample prevalence percentage (≥20%) and relative abundance (≥0.01%) (constructed using MicrobiomeAnalyst 2.0).

**
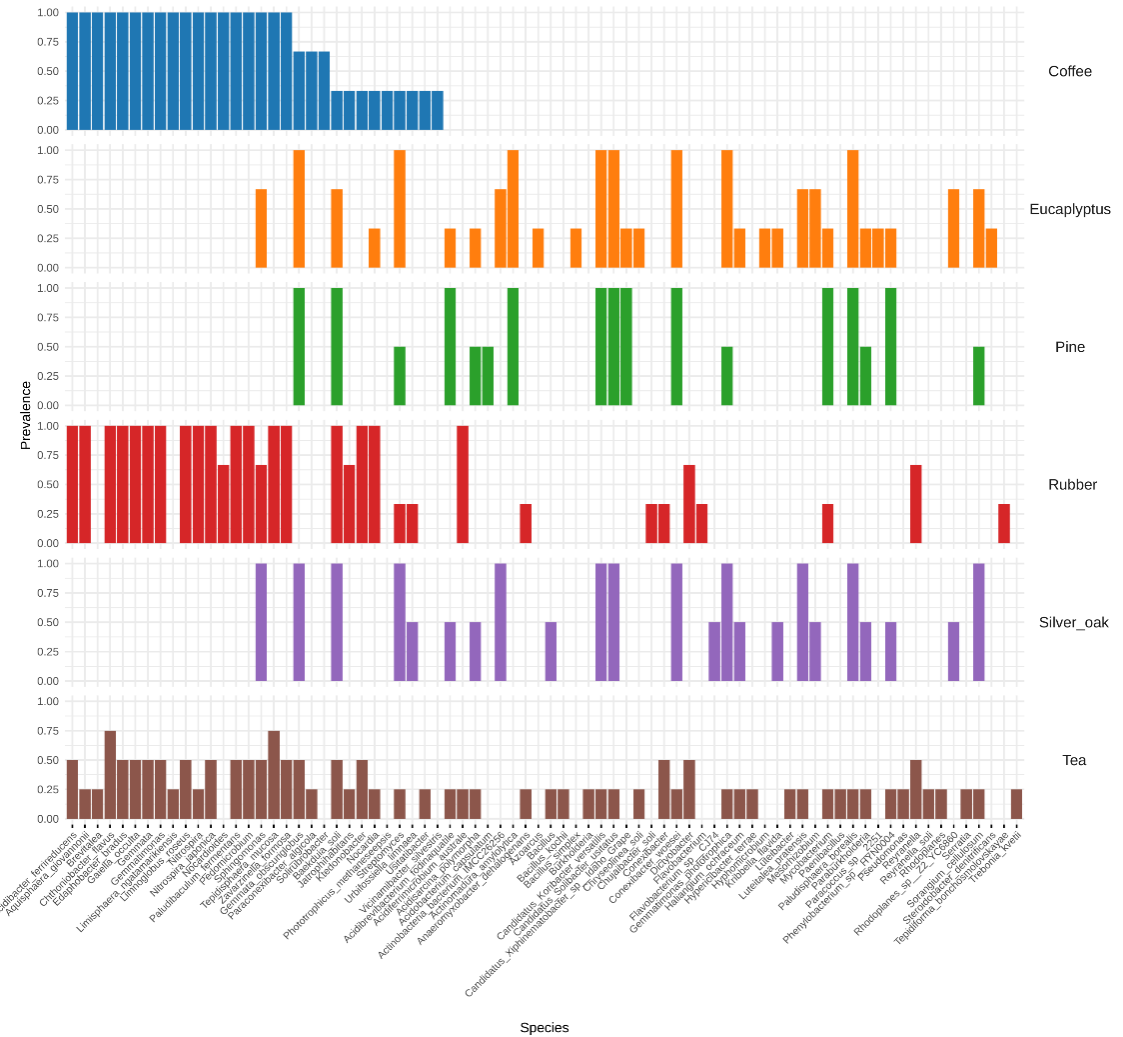
**

**Supplementary Figure 16.** Bar plots depicting the species-level core bacteriome variations across the sample groups based on ‘plant genera’;obtained by applying the parameters of sample prevalence percentage (≥20%) and relative abundance (≥0.01%) (constructed using MicrobiomeAnalyst 2.0).

**
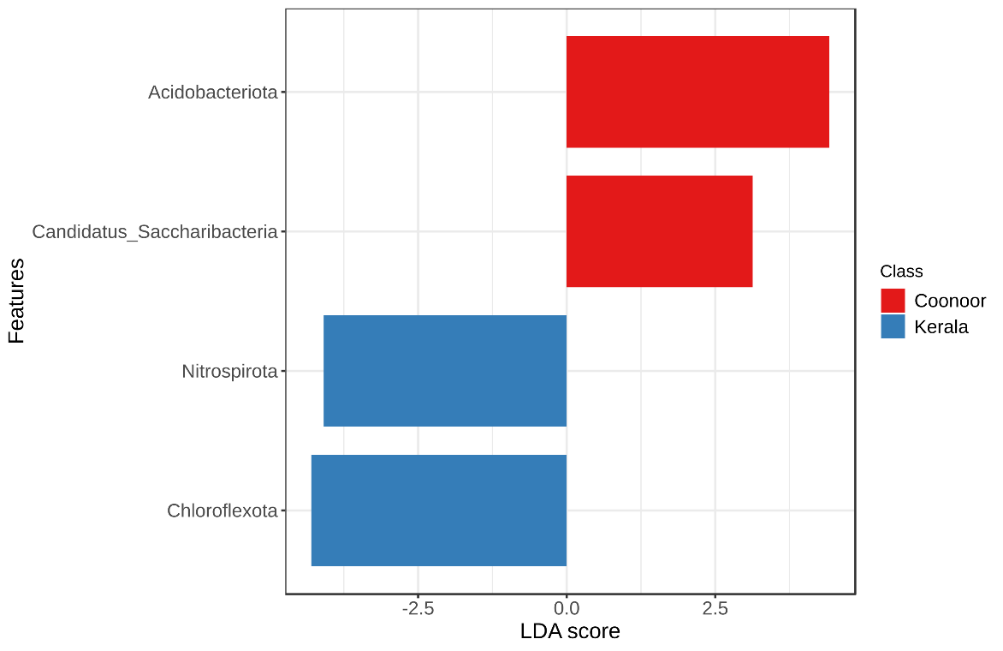
**

**Supplementary Figure 17**. LEfSe analysis of major bacterial phyla across the sampling locations, depicting the differentially abundant phyla using LDA scores > 3 and FDR-adjusted p-value < 0.05.


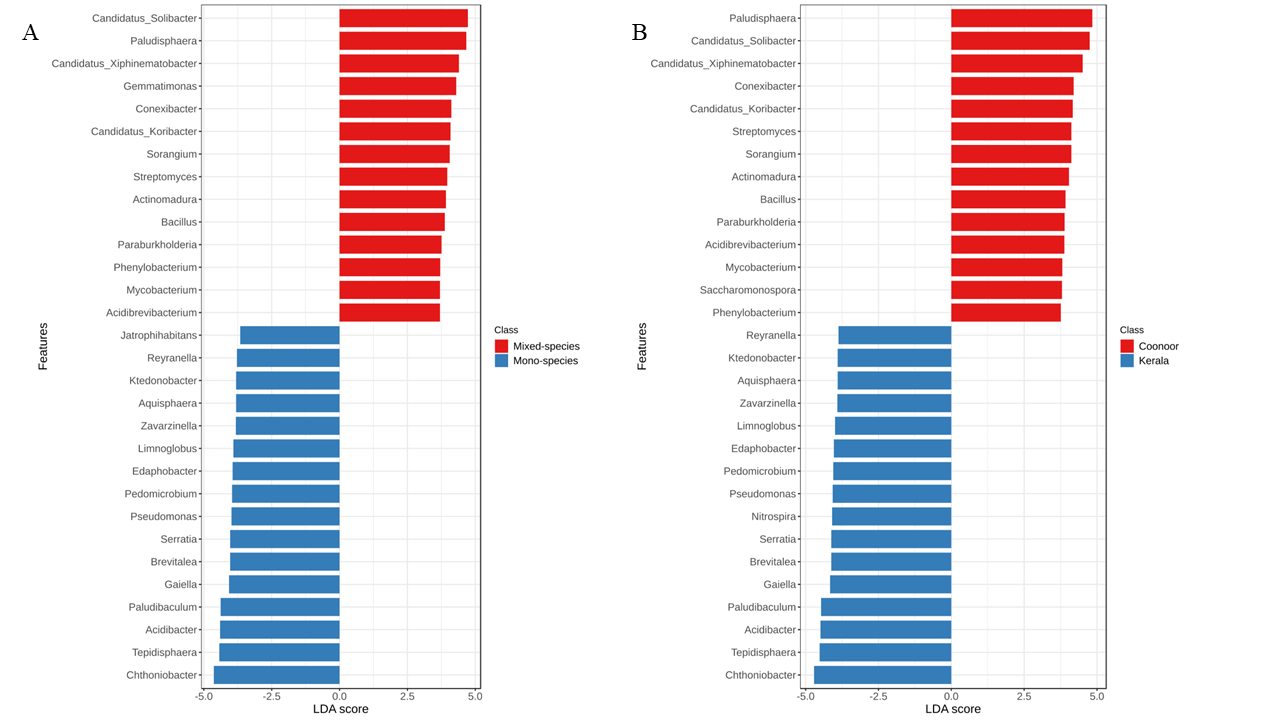


**Supplementary Figure 18**. LEfSe analysis major bacterial genera across the sample groups, depicting the differentially abundant taxa using LDA scores > 3 and FDR-adjusted p-value < 0.05. (A) Plantation type (B) Sampling location

**
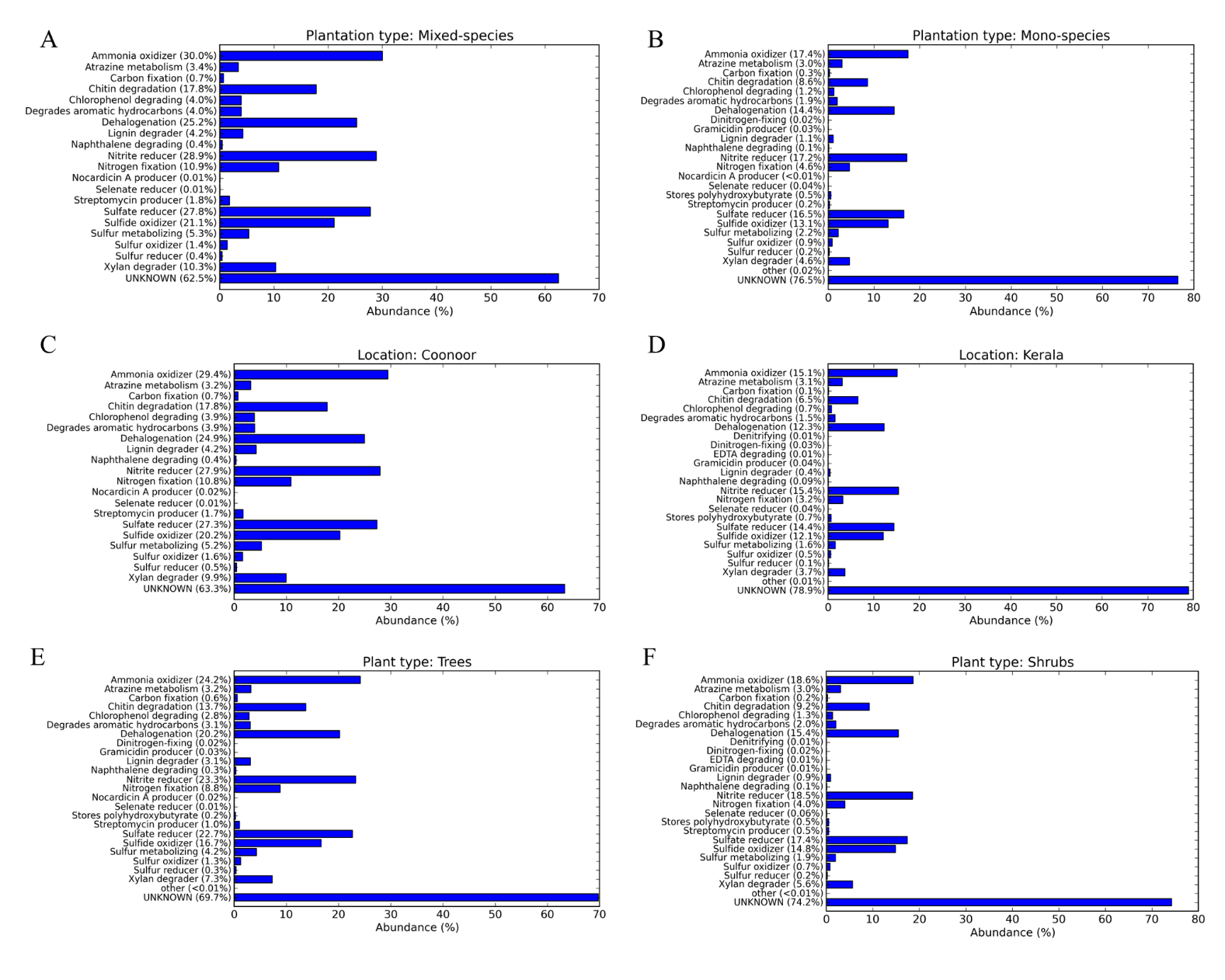
**

**Supplementary Figure 19**. Bar plots depicting the metabolic composition of the rhizosphere metagenomes determined using the taxonomy for phenotype mapping via the MetagenAssist web server, which is based on the plantation type, sample location, and plant type (A) Mixed-species (B) Mono-species (C) Coonoor (D) Kerala (E) Trees (F) Shrubs

**
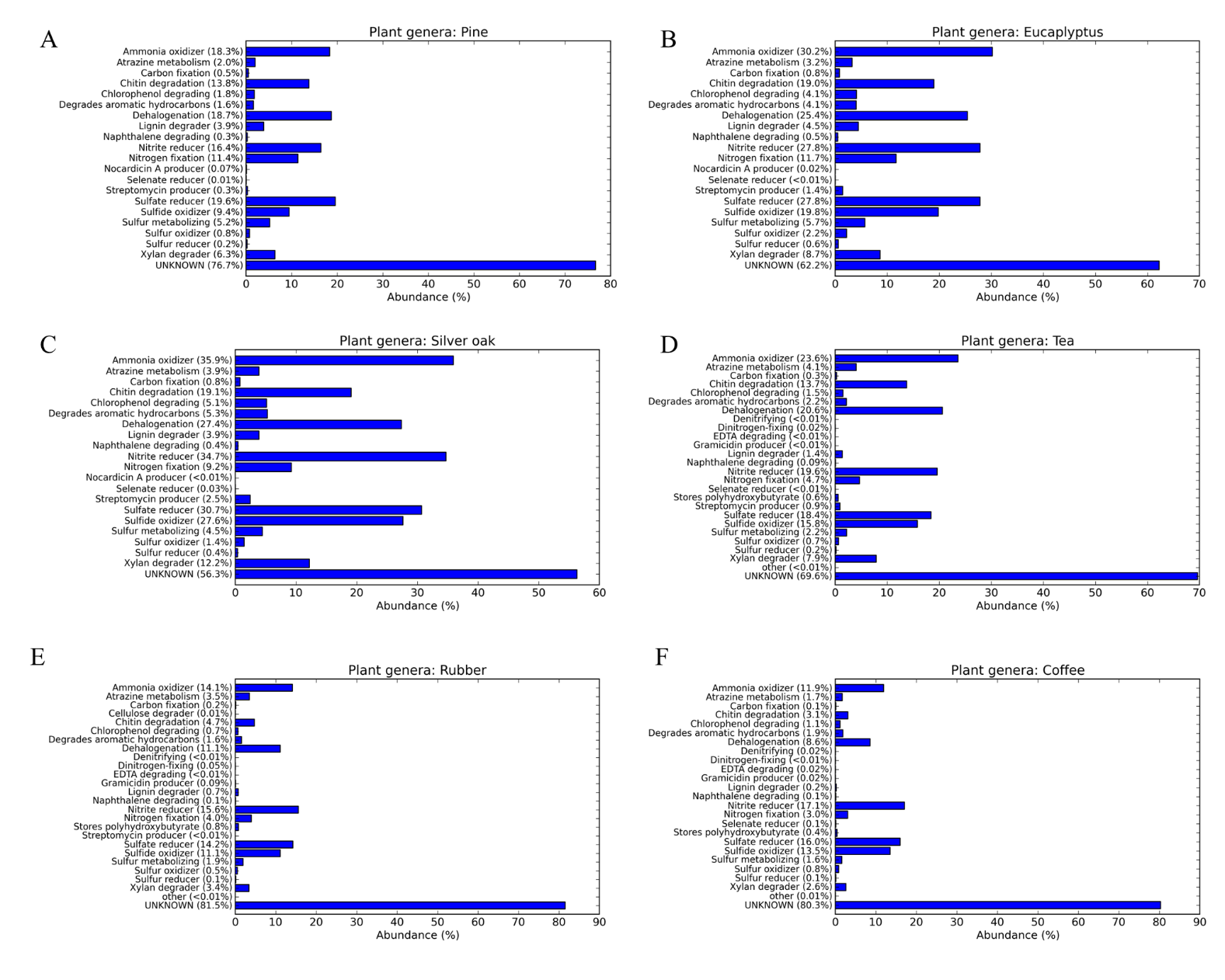
**

**Supplementary Figure 20**. Bar plots depicting the metabolic composition of the rhizosphere metagenomes based on the plant genera determined using the taxonomy for phenotype mapping via the MetagenAssist web server. (A) Pine (B) Eucalyptus (C) Silver oak (D) Tea (E) Rubber (F) Coffee


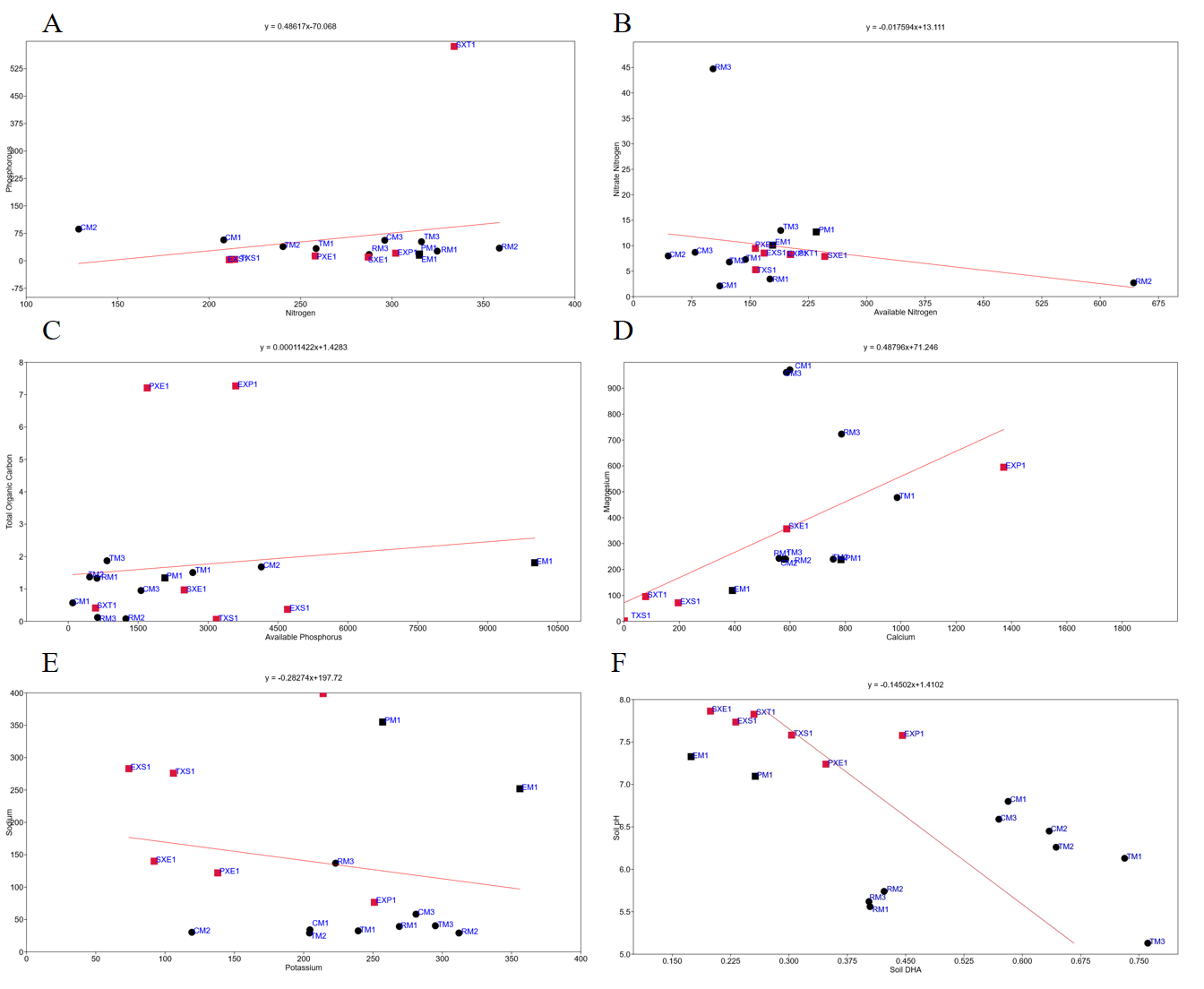


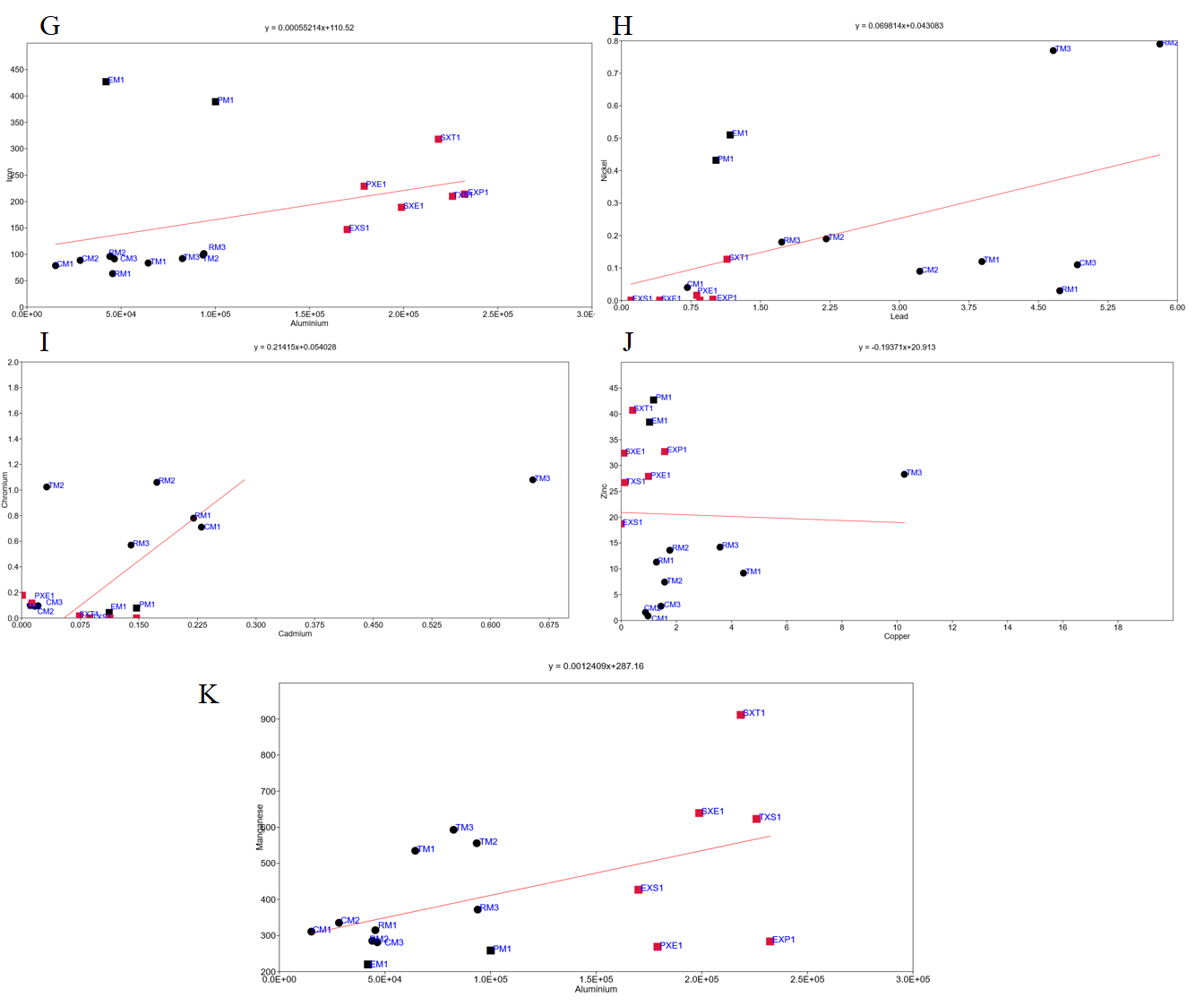


**Supplementary Figure 21**. GLM analysis of soil physicochemical parameters and heavy metal concentrations across the samples. (A) N vs P (B) NNvs AN (C) TOC vs AP(D) Mg vs Ca (E) Na vs K (F) Soil DHA vs soil pH (G) Al vs Fe (H) Pb vs Ni (I) Cd vs Cr (J) Zn vs Cu (K) Mn vs Al


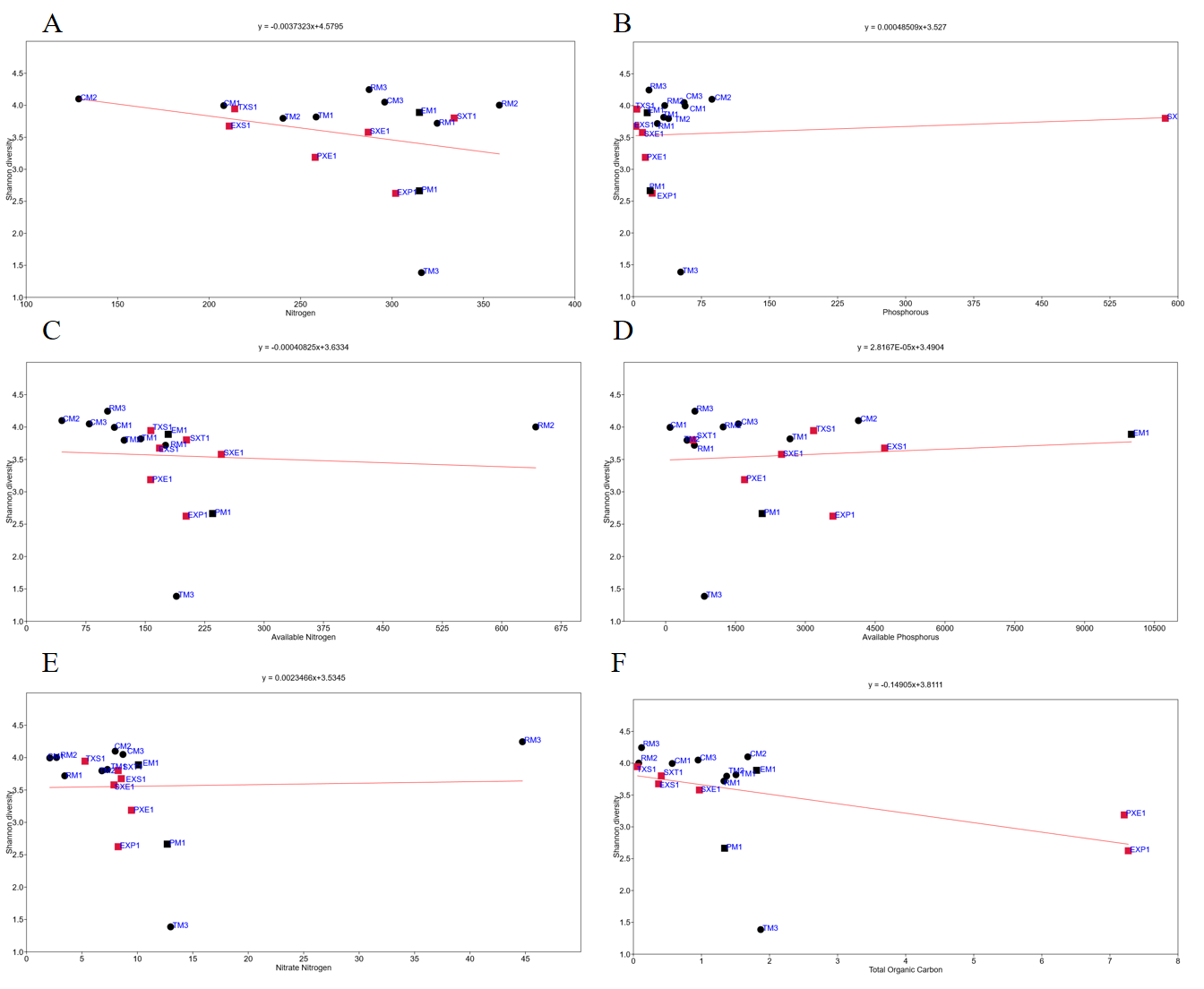


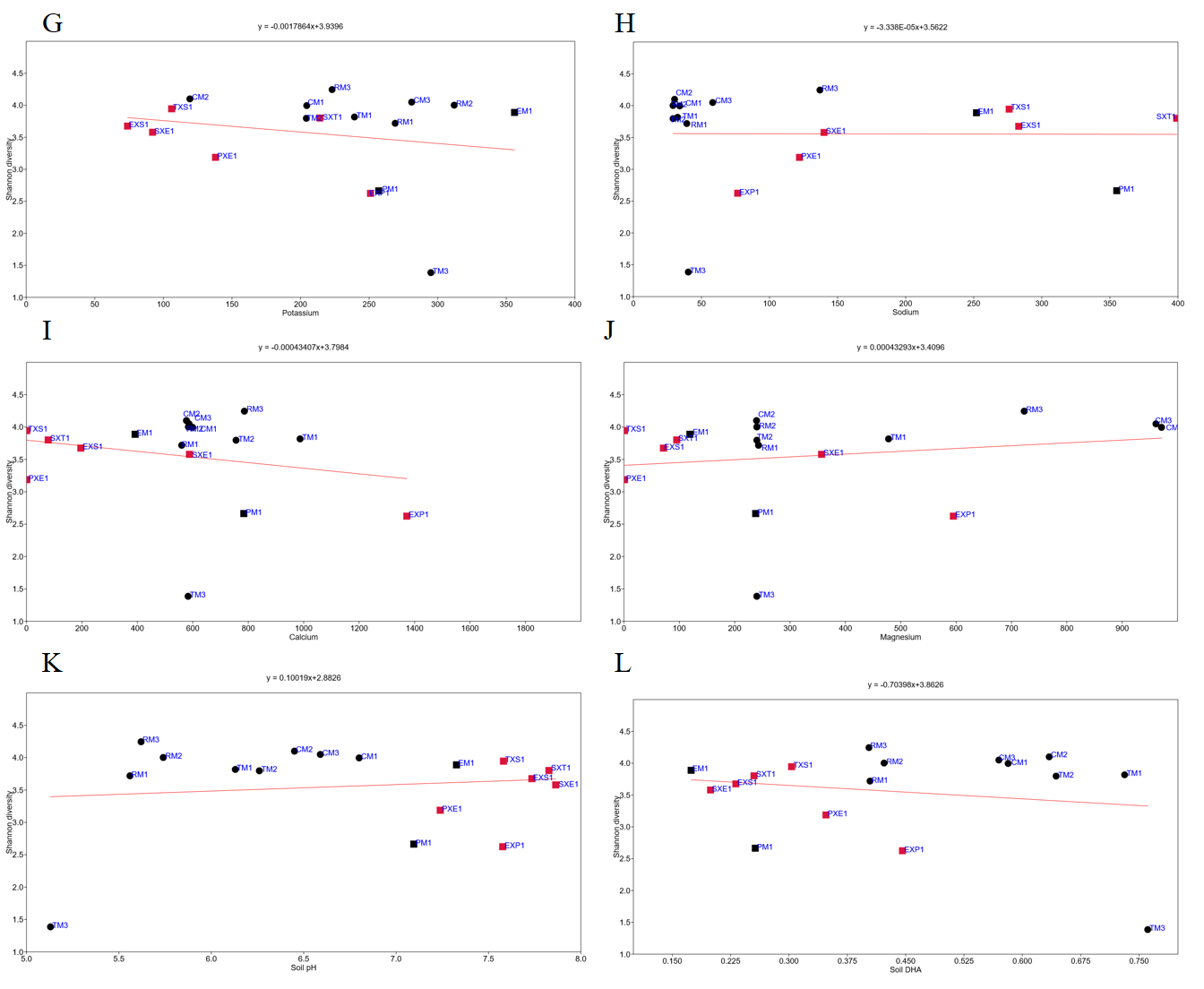


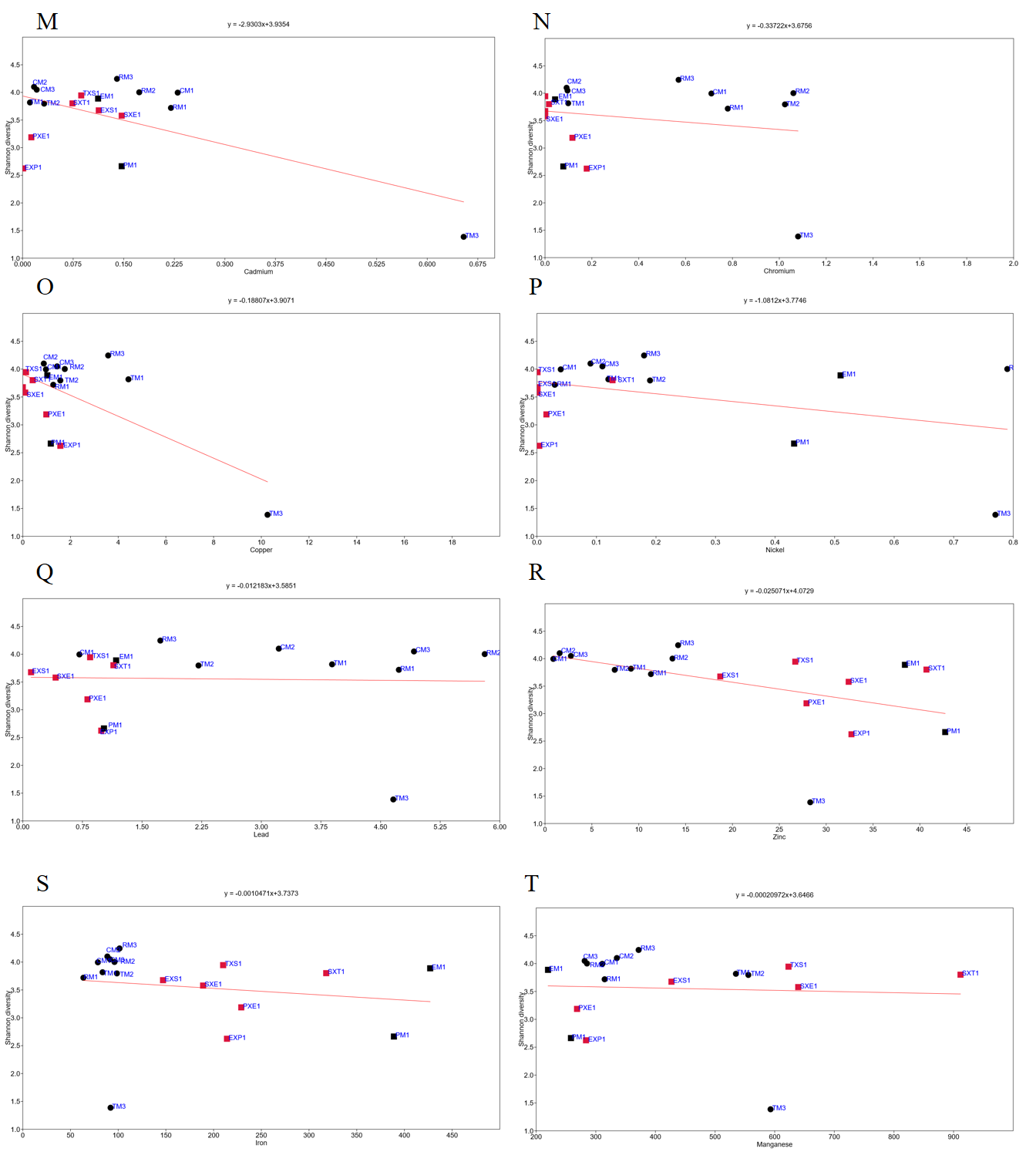


**Supplementary figure 22**. GLM analysis revealing the relationship of soil parameters with the Shannon diversity index across the samples (A) N (B) P (C) AN (D) AP (E) NN (F) TOC (G)Na (H) K (I) Ca (J) Mg (K) Soil pH (L) Soil DHA (M) Cd (N) Cr (O) Cu (P) Ni (Q) Pb (R) Zn (S) Fe (T)Mn
